# Optical-cavity-driven photogenerated charge separation revealed by spatiotemporal imaging

**DOI:** 10.1093/nsr/nwag327

**Published:** 2026-05-29

**Authors:** Chenwei Ni, Thomas Dittrich, Jianbo Tang, Guanhua Zhang, Qian Li, Meng Liu, Fengke Sun, Bing Huang, Yixian Cao, Wenming Tian, Kaifeng Wu, Fengtao Fan, Ruotian Chen, Can Li

**Affiliations:** State Key Laboratory of Catalysis, Dalian National Laboratory for Clean Energy, Dalian Institute of Chemical Physics, Chinese Academy of Sciences, Dalian 116023, China; University of Chinese Academy of Sciences, Beijing 100049, China; Helmholtz-Center Berlin for Materials and Energy GmbH, Berlin D-12489, Germany; State Key Laboratory of Chemical Reaction Dynamics, Dalian Institute of Chemical Physics, Chinese Academy of Sciences, Dalian 116023, China; State Key Laboratory of Chemical Reaction Dynamics, Dalian Institute of Chemical Physics, Chinese Academy of Sciences, Dalian 116023, China; State Key Laboratory of Catalysis, Dalian National Laboratory for Clean Energy, Dalian Institute of Chemical Physics, Chinese Academy of Sciences, Dalian 116023, China; University of Chinese Academy of Sciences, Beijing 100049, China; State Key Laboratory of Chemical Reaction Dynamics, Dalian Institute of Chemical Physics, Chinese Academy of Sciences, Dalian 116023, China; State Key Laboratory of Chemical Reaction Dynamics, Dalian Institute of Chemical Physics, Chinese Academy of Sciences, Dalian 116023, China; State Key Laboratory of Catalysis, Dalian National Laboratory for Clean Energy, Dalian Institute of Chemical Physics, Chinese Academy of Sciences, Dalian 116023, China; University of Chinese Academy of Sciences, Beijing 100049, China; State Key Laboratory of Catalysis, Dalian National Laboratory for Clean Energy, Dalian Institute of Chemical Physics, Chinese Academy of Sciences, Dalian 116023, China; University of Chinese Academy of Sciences, Beijing 100049, China; State Key Laboratory of Chemical Reaction Dynamics, Dalian Institute of Chemical Physics, Chinese Academy of Sciences, Dalian 116023, China; State Key Laboratory of Chemical Reaction Dynamics, Dalian Institute of Chemical Physics, Chinese Academy of Sciences, Dalian 116023, China; State Key Laboratory of Catalysis, Dalian National Laboratory for Clean Energy, Dalian Institute of Chemical Physics, Chinese Academy of Sciences, Dalian 116023, China; State Key Laboratory of Catalysis, Dalian National Laboratory for Clean Energy, Dalian Institute of Chemical Physics, Chinese Academy of Sciences, Dalian 116023, China; State Key Laboratory of Catalysis, Dalian National Laboratory for Clean Energy, Dalian Institute of Chemical Physics, Chinese Academy of Sciences, Dalian 116023, China; University of Chinese Academy of Sciences, Beijing 100049, China

**Keywords:** charge separation, optical cavity, surface photovoltage, spatiotemporal imaging, carrier diffusion dynamics

## Abstract

Photogenerated charge separation across micro- to nanometer scales is essential for photoelectric and photocatalytic conversion. However, identifying microstructures that sustain efficient charge separation and elucidating the underlying mechanisms remain challenging. Here, by combining surface photovoltage microscopy with optical imaging, we show that optically resonant cavity structures generate highly non-uniform light-field distributions that subsequently drive efficient charge separation through asymmetric electron and hole diffusivities. Spatiotemporal imaging of carrier dynamics from femtoseconds to seconds reveals that this charge separation originates from the combined contributions of ultrafast hot-electron diffusion (∼3 ps) and long-lived trap-limited transport (∼5 ms). Leveraging these effects, we demonstrate control over both the magnitude and direction of charge separation via optical structure engineering. These findings deepen the fundamental understanding of diffusion-driven charge separation in semiconductors and establish optical-architecture engineering as a viable approach for manipulating this process, providing a blueprint for advancing solar energy conversion and optoelectronic technologies.

## Teaser

Spatiotemporal imaging reveals how optical cavities generate uneven light fields that drive efficient charge separation via asymmetric carrier diffusion, offering a strategy for advancing solar and optoelectronic technologies.

## INTRODUCTION

Photogenerated charge separation at semiconductor surfaces and interfaces across micro- to nanometer length scales underpins a wide range of emerging photoelectric technologies [1–3], including the conversion of light into electrical [4,5] and chemical [6,7] energy. Fundamentally, charge separation arises from asymmetric electron and hole transport driven by gradients in electric potential or chemical potential (carrier concentration) [8]. To promote the separation of electrons and holes toward distant surface sites for electrical extraction or catalytic reactions, numerous micro- and nanostructural strategies have been developed, including engineering anisotropic crystal facets [9,10], constructing heterogeneous or heterophase junctions [11,12] and designing Z-scheme architectures [13,14]. These established approaches primarily exploit built-in electric potential gradients to drive electrons and holes in opposite directions, thereby enhancing photoelectric conversion efficiency.

As an alternative and more general mechanism, charge separation driven by carrier diffusion has been widely observed in diverse semiconductor systems [15–19], providing a complementary pathway that operates independently of electric potential gradients. In this mechanism, charge separation originates from differences in electron and hole mobilities as they diffuse down concentration gradients created by spatially non-uniform light absorption, as described by the Dember effect [20,21]. Despite its fundamental significance, however, few strategies have been proposed to deliberately harness or amplify diffusion-driven charge separation, leaving its practical potential largely untapped.

Photonic structures offer a promising route to address this challenge by enabling controlled confinement and redistribution of light at the micro- and nanoscale [22–24], thereby providing a means to manipulate diffusion-driven charge separation. To date, however, most photonic designs have focused on enhancing light-harvesting efficiency, with little attention paid to how optical confinement shapes the ensuing charge-separation behavior [25,26]. Moreover, the spatiotemporal evolution of charge separation in these structures remains poorly understood, and key mechanistic insights are still lacking.

Here, as a proof-of-concept study, we combine surface photovoltage microscopy (SPVM) with optical imaging to demonstrate that optical-cavity structures can effectively enable diffusive charge separation at semiconductor surfaces. Direct spatiotemporal tracking from femtoseconds to seconds at the nanoscale reveals that this diffusive charge separation originates from ultrafast hot-electron diffusion within ∼3 ps and long-lived trap-limited transport at ∼5 ms. By tuning cavity sizes to engineer local light-field distributions, we gain control over charge-separation behavior. These findings advance the fundamental understanding of diffusion-driven charge separation and provide a practical strategy for manipulating charge separation in optoelectronic devices and solar energy conversion via photonic structure engineering.

## RESULTS AND DISCUSSION

### Mapping cavity-induced charge separation

To trigger carrier diffusion through non-uniform light absorption, we explored optical-cavity structures that can locally confine light and enhance absorption via resonant recirculation [27–29]. These cavity architectures were fabricated on Cu_2_O particles, a semiconductor widely employed in photoelectric conversion [30–32]. By regulating crystal growth between branching and faceted modes (Fig. S1), the cavity size could be systematically tuned on individual Cu_2_O particles (Fig. S2).

**Figure 1.**
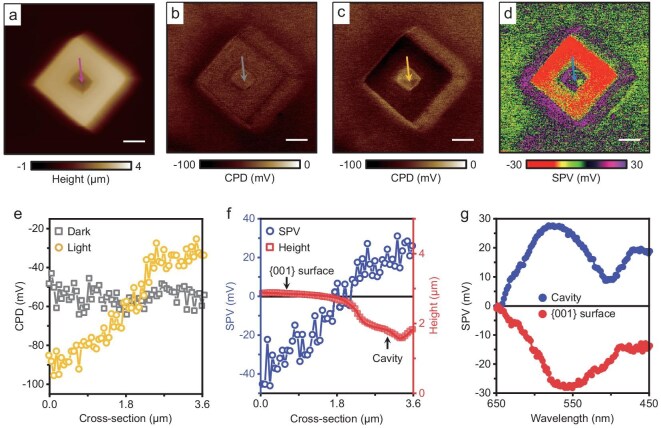
Charge separation between cavity and {001} surface regions. (a–d) AFM image (a), KPFM images in the dark (b) and under 450-nm light illumination (c), and the corresponding SPVM image (d) of a microcavity-structured Cu_2_O particle. The SPVM image in panel (d) is obtained by subtracting the dark KPFM image in panel (b) from the illuminated KPFM image in panel (c). Scale bars, 2 μm. (e) Line profiles of the contact potential difference extracted along the arrow in panels (b) and (c). (f) Line profiles of SPV and height values extracted along the arrow in panels (d) and (a), respectively. (g) SPV spectra acquired at the cavity and {001} surface regions. CPD: contact potential difference.

Figure 1a shows an atomic force microscopy (AFM) image of a representative cubic Cu_2_O particle with a microcavity centered on its {001} facet. Kelvin probe force microscopy (KPFM) [33] measurements under dark conditions reveal a uniform surface potential across the {001} surface and the cavity region (Fig. 1b and e), indicating the absence of lateral built-in electric fields. Finer-scale measurements further confirm no built-in potential differences at the cavity edges or between lateral facets and the {001} surface (Fig. S3). Upon illumination, the surface potential increases within the cavity and decreases on the surrounding {001} surface (Fig. 1c), generating a lateral potential difference of ∼60 mV (Fig. 1e). These photo-induced potential changes provide direct evidence of lateral charge separation between the cavity and {001} surface regions.

We used SPVM to map the spatial distribution of photogenerated carriers, where positive and negative surface photovoltage (SPV) signals correspond to holes and electrons, respectively [34,35]. The SPVM image shows that holes and electrons are efficiently directed to the cavity and {001} surface regions, respectively (Fig. 1d and Fig. S4). In contrast, particles without cavities exhibit no such charge separation (Fig. S5), indicating that the cavity architecture is essential for this behavior (Fig. 1f). Spatially resolved SPV spectroscopy (Fig. 1g) confirms the persistence of this separation under above-band gap excitation (*E*_g_ ∼ 620 nm). The magnitude of charge separation depends on both the excitation wavelength and light polarization (Fig. S6), and the SPV signal within the cavity saturates more rapidly with increasing light intensity (Fig. S7). These observations support that cavity-induced optical effects drive the charge separation. High-resolution scanning transmission electron microscopy combined with electron energy loss spectroscopy and spatially resolved X-ray spectroscopy reveals no discernible differences in electronic structure or defect states between the cavity and {001} surface regions (Figs S8–S10), consistent with uniform potential distributions. These results rule out built-in electric fields and defect trapping effects [36,37] as the dominant origin of the lateral charge separation. Taken together, we reasonably attribute the charge separation to cavity-induced asymmetry in light absorption and the ensuing diffusive transport of photogenerated carriers.

### Mapping light-field distribution

**Figure 2.**
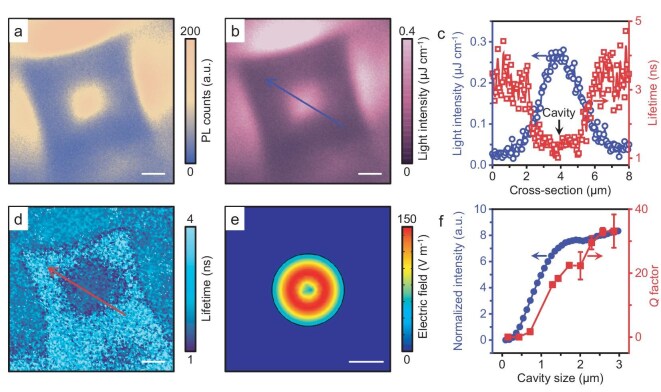
Light-field imaging and simulations. (a, b) PL image (a) and reconstructed light-intensity map (b) of a cavity-structured Cu_2_O particle coated with CdS QDs. The light-intensity map in panel (b) was derived from panel (a) using the calibrated relationship between PL intensity and local light intensity (Fig. S15). Scale bars, 2 μm. (c) Line profiles of light intensity (blue) and PL lifetime (red) extracted along the arrows in panels (b) and (d), respectively. (d) PL lifetime image mapped by time-resolved and PL-scanned confocal microscopy. The lifetime at each pixel was determined from an exponential fit of the PL decay. Scale bar, 2 μm. (e) Simulated optical field distribution in the *x*–*y* plane showing cavity-induced local electric-field enhancement via whispering-gallery-mode resonance. Scale bar, 2 μm. (f) Simulated electric-field enhancement and cavity *Q* factor as a function of cavity size. The electric-field intensity was normalized to the intensity at the {001} surface. *Q* factors were obtained from whispering-gallery-mode resonances at each cavity size (Fig. S19). Error bars represent variation in *Q* values across different resonance frequencies.

To experimentally validate the cavity-induced non-uniform light-field distribution underlying the charge separation, we employed CdS quantum dots (QDs, Fig. S11) as fluorescent probes [38] and mapped their photoluminescence (PL) intensity to reflect the local light-field strength. Pristine Cu_2_O exhibits no detectable PL, as its emission is quenched at room temperature due to dominant non-radiative recombination [39,40], whereas CdS QDs deposited on Cu_2_O display a pronounced emission peak at ∼450 nm, corresponding to the band-edge emission of CdS [41] (Fig. S12a). Energy-dispersive X-ray spectroscopy in scanning electron microscopy imaging confirms the uniform distribution of CdS QDs across the Cu_2_O particles (Fig. S13), ensuring the reliability of this labeling approach. Figure 2a shows a representative PL image of a microcavity-structured Cu_2_O particle decorated with CdS QDs, collected at 450 nm, revealing a pronounced enhancement of PL intensity within the cavity compared with the {001} surface. Spatially resolved PL spectra further confirm this enhancement (Fig. S12b), indicating that the emission peak intensity in the cavity is ∼9.5 times higher than that on the {001} surface. A similar cavity-induced PL enhancement is also observed when collecting emission at longer wavelengths (Fig. S14), confirming the robustness and generality of the observed spatial contrast. To quantify the light-field enhancement underlying this PL contrast, we calibrated the relationship between PL intensity and excitation intensity (Fig. S15) and converted the PL maps into a spatially resolved local light-field intensity distribution (Fig. 2b). Quantitative analysis reveals that the local light intensity inside the cavity is enhanced by a factor of 8–10 compared with that outside the cavity (Fig. 2c, blue points).

To verify that this enhancement originates from cavity-induced optical resonance, we performed time-resolved PL imaging to examine modifications of the radiative lifetime, as expected from the Purcell effect [42,43]. The PL lifetime map shows a pronounced reduction within the cavity relative to the flat {001} surface (Fig. 2d), decreasing from ∼3.4 ns to ∼1.5 ns (Fig. 2c, red points). Control measurements show that the PL lifetime on the {001} surface remains essentially independent of excitation intensity over the measured range (Fig. S16), ruling out locally enhanced excitation intensity as the origin of the reduced lifetime. The observed lifetime reduction corresponds to a Purcell factor of ∼2.3. Based on this value and the simulated effective mode volume (Fig. S17), the microcavity quality factor (*Q*) is estimated to be in the order of several tens (Supplementary Note 1). The *Q* characterizes the extent of multiple light–matter interactions within the cavity, thereby increasing the probability of light absorption relative to single-pass reflection losses. This *Q* value is consistent with the experimentally observed ∼8-fold enhancement of local light absorption, supporting an optical-resonance origin of the enhancement.

To further corroborate these experimental observations, we simulated the electromagnetic field distribution in three-dimensional Cu_2_O cavity structures (Fig. S18). Eigenmode analysis [44] (Fig. S19) indicates that the microcavities enable optical resonances via whispering-gallery modes [27,45,46], where the electromagnetic fields are primarily confined within the lateral (*x*–*y*) plane and are therefore only weakly affected by the cavity depth (*z* direction), as further supported by depth-dependent simulations (Fig. S20). The *x*–*y* plane projection of the simulated electromagnetic field, integrated along the *z* direction, thus reproduces the experimentally observed spatial light-field distribution (Fig. 2e). Quantitative simulations across varying cavity sizes (Figs S19 and S21) and different cavity shapes (Fig. S22) indicate that the field-intensity enhancement depends only on cavity shape but increases with increasing cavity size. Simulations performed under the experimental conditions (cavity size ∼2 μm, excitation wavelength 450 nm) yield a whispering-gallery resonance quality factor of ∼25 (Fig. 2f), in good agreement with the experimental determination. Furthermore, simulations across a range of cavity sizes show strong consistency with the experimental results (Fig. S23), thereby establishing cavity-induced optical resonance as the physical origin of the observed non-uniform light absorption.

### Mapping diffusive charge-separation dynamics

**Figure 3.**
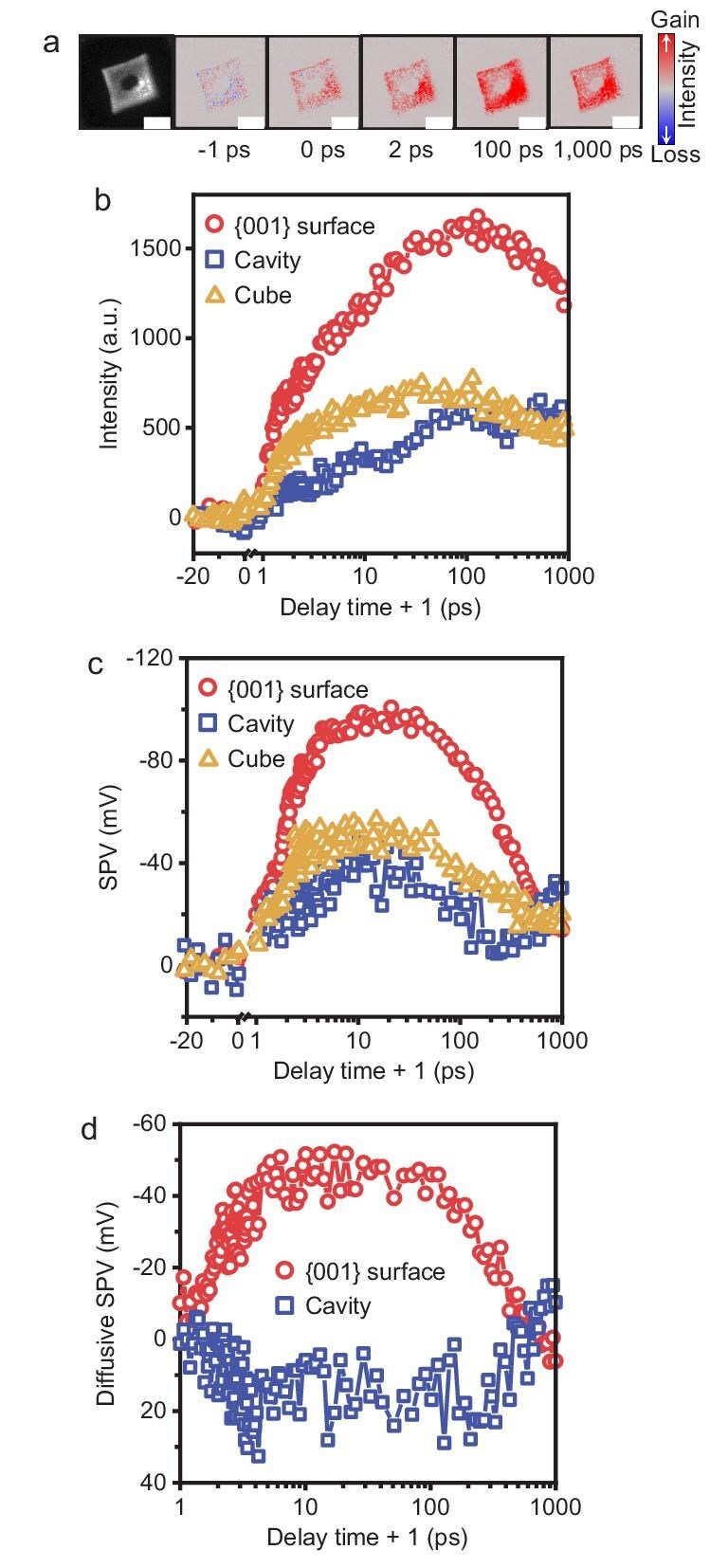
Ultrafast charge-transfer dynamics. (a) PEEM image and a series of TR-PEEM images of a cavity-structured Cu_2_O particle at selected pump–probe delay times. TR-PEEM images were obtained by subtracting the averaged image at negative delay times. Scale bars, 5 μm. (b) TR-PEEM signals extracted from the cavity and {001} surface regions in panel (a), as well as from cubic Cu_2_O (Fig. S26a). (c) Time-resolved SPV signals derived from peak shifts in photoelectron spectroscopy at different delay times for the cavity and {001} surface regions of cavity-structured Cu_2_O (Fig. S25) and for cubic Cu_2_O (Fig. S26b). (d) Diffusive SPV signals plotted as a function of delay time for the cavity and {001} surface regions of cavity-structured Cu_2_O. These signals were obtained by subtracting the SPV response of cubic Cu_2_O, which reflects charge separation solely in the p-type surface space-charge region.

To directly visualize how non-uniform light absorption drives sequential diffusive charge separation, we tracked the dynamic evolution of cavity-induced charge separation using spatiotemporally resolved SPV techniques [6,35]. Time-resolved photoemission electron microscopy (TR-PEEM) was first employed to probe charge-separation dynamics on femtosecond-to-nanosecond timescales [47,48]. In the TR-PEEM measurements, 2.4-eV femtosecond laser pulses were used for photoexcitation, and time-delayed 4.8-eV pulses were used to emit photogenerated electrons into vacuum, which were subsequently imaged by photoemission electron microscopy (PEEM). TR-PEEM images acquired at different pump–probe delays (Fig. 3a and Fig. S24) directly map the transfer of photogenerated electrons to the flat {001} surface that occurs on picosecond timescales. Figure 3b extracts and compares the charge-transfer dynamics of the cavity and {001} surface regions. For quantitative analysis, the corresponding spatiotemporally resolved SPV signals (Fig. 3c) were derived from peak shifts in time-resolved photoelectron spectroscopy (Fig. S25), with additional measurements performed on cubic Cu_2_O surfaces for comparison (Fig. S26).

Immediately after photoexcitation, the TR-PEEM signals rapidly increase within several picoseconds (Fig. 3b). Coinciding with this, negative SPV signals appear (Fig. 3c), corresponding to electron transfer to the surface. Over the subsequent ∼100 ps, electron transfer continues to increase the TR-PEEM intensity, albeit at a much slower rate. In contrast, the negative SPV signals remain nearly constant over tens of picoseconds. Because SPV depends on both charge density and electron energy, this apparent discrepancy reflects the reduction of electron energy despite the continued accumulation of electrons at the surface. The reduced electron transfer rate supports this interpretation, as energy relaxation significantly decreases electron mobility, consistent with previous observations in Cu_2_O [6] and other semiconductors [17,18].

Both cavity-structured and cubic Cu_2_O exhibit positive TR-PEEM signals and negative SPV signals owing to the p-type surface space charge region, which drives electrons to the surface. To remove this vertical bulk-to-surface contribution, we subtracted the SPV signals measured on cubic Cu_2_O, which exhibit only vertical electron transfer. The resulting SPV signals (Fig. 3d) are thus solely associated with lateral diffusive charge separation between the cavity and the {001} surface regions. Notably, these signals emerge simultaneously in the two regions, becoming negative on the {001} surface and positive within the cavity, directly evidencing carrier diffusion between them. This ultrafast diffusion occurs within ∼3 ps, comparable to the timescale of hot-electron relaxation via electron–phonon interactions [49]. We therefore modeled the dynamics using a modified diffusion equation (Supplementary Note 2), in which the diffusivity decays exponentially with hot-electron energy relaxation [50]. The fit yields a diffusivity of 1500 cm^2^ s^−1^ and a relaxation time constant of 1.5 ps (Fig. S27a). These parameters correspond to an electron transfer velocity of 6.3 × 10^5^ m s^−1^, consistent with hot-electron transport in Cu_2_O [6], indicating that the ultrafast diffusion-induced SPV signals arise from the diffusion of hot electrons that are not fully thermalized. At longer delay times, the diffusion-induced SPV signals decay over hundreds of picoseconds. Since the photoelectron intensity remains nearly constant on this timescale, we attribute this slower process to hole diffusion. Fitting this regime yields a diffusivity of 3.3 cm^2^ s^−1^ (Fig. S27b), consistent with the reported hole mobility of Cu_2_O [51]. Strikingly, the pronounced asymmetry between electron and hole diffusivities (1500 cm^2^ s^−1^ vs 3.3 cm^2^ s^−1^) underlies the steady-state lateral diffusive charge separation observed in these cavity-structured particles (see the quantitative relationship between steady-state diffusive SPV and diffusivity asymmetry in Supplementary Note 3).

**Figure 4.**
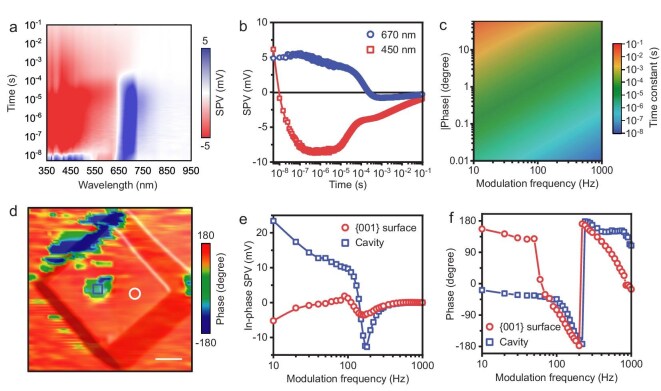
Trap-limited charge transfer. (a) Transient SPV spectrum of microcavity-structured Cu_2_O particles. (b) Representative SPV transients under super-band gap (450 nm) and sub-band gap (670 nm) excitations, extracted from panel (a). (c) Relationship between the transient SPV time constant and the phase under chopped-light modulation at different frequencies. (d) Phase image of modulated SPV signals measured at 20 Hz for a microcavity-structured Cu_2_O particle. Scale bar, 2 μm. (e, f) In-phase SPV (e) and corresponding phase (f) plotted as a function of modulation frequency for the cavity and {001} surface regions indicated in panel (d).

Next, we examined charge dynamics on nanosecond-to-millisecond timescales. Under super-band gap excitation, the transient photovoltage (TPV) spectrum of cavity-structured Cu_2_O (Fig. 4a) exhibits an initial positive SPV transient before 10^−8^ s, followed by negative SPV signals from 10^−8^ to 10^−1^ s (Fig. 4b, red line). Positive SPV transients were also observed under sub-band gap excitation (Fig. 4b, blue line), attributed to electron trapping in near-surface defect states [36]. The sub-band gap SPV response in the 650–730 nm range corresponds to the energy levels of oxygen vacancies in Cu_2_O [52,53], which are likely responsible for these electron trap states. Such sub-nanosecond electron trapping in Cu_2_O has been previously reported [54] and provides a consistent explanation for the gradual energy relaxation on tens-of-picoseconds timescales observed in the TR-PEEM measurements above. These trapped electrons persist up to 10^−5^–10^−4^ s, after which de-trapping occurs, allowing electron transfer to the surface and decay of the positive SPV signal. The dominant negative SPV signals under super-band gap excitation correspond to bulk-to-surface charge separation in the p-type space charge region. However, lateral charge separation between the cavity and {001} surface regions cannot be resolved by TPV due to ensemble averaging [55].

To capture lateral charge separation on nanosecond-to-millisecond timescales, we extended the time resolution of SPVM, typically limited to ∼1 ms [35], by employing modulated light illumination and extracting the phase of the modulated SPV signals via a lock-in amplifier (schematically shown in Fig. S28). Numerical analysis establishes a quantitative relationship between the SPV phase and the characteristic time constant under modulated illumination (see Supplementary Note 4 for derivations). As shown in Fig. 4c, this approach enables time-resolved detection with a precision down to tens of nanoseconds under a 1-kHz modulation frequency. Figure 4d maps the SPV phase for a microcavity-structured Cu_2_O particle under 20-Hz chopped 450-nm illumination. A phase contrast of ∼180° between the cavity and {001} surface is clearly resolved, indicating direct lateral charge separation between the two regions.

To decipher the dynamical evolution of charge separation between the two regions, we measured the modulated SPV signals at different modulation frequencies (Fig. 4e and f). At modulation frequencies above 200 Hz, SPV signals from both regions are nearly aligned and negative, consistent with the ensemble-averaged negative SPV observed in TPV measurements on timescales of 10^−8^ to 10^−5^ s. At lower modulation frequencies, the in-phase SPV signals evolve toward positive and negative values for the cavity and {001} surface regions, respectively, while their phase lags maintain an approximately constant difference of ∼180°, directly visualizing dynamic lateral charge separation. The transition between these two regimes occurs between 100 and 200 Hz, corresponding to a derived time constant of 1.1 × 10^−4^ s, in line with the electron de-trapping process identified in TPV. Phase lags in the 10–100 Hz range correspond to a time constant of ∼5 × 10^−3^ s, indicating the characteristic timescale of lateral diffusive charge separation following de-trapping.

Together, these spatiotemporal imaging results provide a comprehensive picture of cavity-induced charge separation. Upon photoexcitation, the microcavity-induced whispering-gallery optical resonances locally enhance light absorption by roughly an order of magnitude, generating a significantly higher density of photocarriers within the cavity regions. A fraction of the non-thermalized hot electrons exhibits diffusivities of two to three orders of magnitude higher than those of holes, enabling efficient electron diffusion toward the {001} surface regions within ∼3 ps. The remaining electrons undergo thermalization and become trapped on subsequent tens-of-picoseconds timescales. These trapped electrons remain inactive until de-trapping occurs on tens-of-microseconds timescales, after which they resume diffusion toward the {001} surface regions with a characteristic time constant of ∼5 ms. Collectively, these results identify the origin of the diffusive charge separation as the combined effects of ultrafast hot-electron diffusion and trap-limited transport at much longer timescales.

### Manipulating charge separation via optical control

Optical-effect-induced charge separation establishes a foundation for manipulating photogenerated charge separation via cavity-structure engineering. Simulations (Fig. 2f) show that the light-field asymmetry between the cavity and the {001} surface can be tuned by adjusting the cavity size. For cavities larger than the excitation wavelength, whispering-gallery resonances enhance the intracavity light intensity, which increases with cavity size, thereby strengthening diffusive charge separation and enabling control over its magnitude. Conversely, for cavities smaller than the wavelength, the optical mode transitions into a zero-mode waveguide regime [56,57], where the intracavity field is exponentially attenuated along the subwavelength cavity. This reverses the light-field asymmetry between the cavity and the {001} surface, providing a mechanism to control the direction of charge separation.

**Figure 5.**
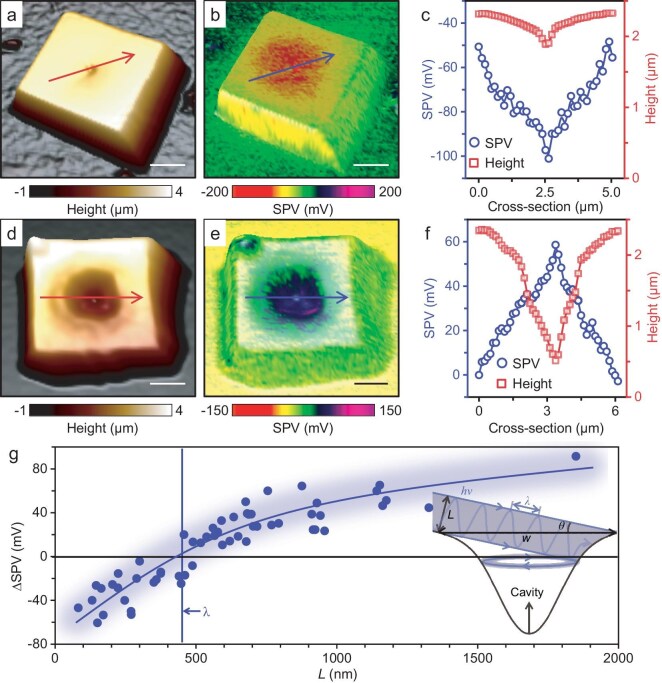
Manipulation of cavity-induced charge separation by cavity size engineering. (a, b) AFM (a) and SPVM (b) images of a Cu_2_O particle with a small cavity size. Scale bars, 2 μm. (c) Line profiles of SPV and height extracted along the arrow in panels (b) and (a), respectively. (d, e) AFM (d) and SPVM (e) images of a Cu_2_O particle with a large cavity size. Scale bars, 2 μm. (f) Line profiles of SPV and height extracted along the arrow in panels (e) and (d), respectively. (g) Difference in SPV between the cavity and {001} surface regions (ΔSPV) as a function of effective cavity size (*L*). Inset is schematic of the geometry of light incidence into the cavity during SPVM measurements. Given this geometry, the effective cavity size is defined as *L* = *w* · sin*θ*, where *w* is the physical cavity width and θ is the glancing angle of the incident light. λ and *hν* denote the wavelength and photon energy of the incident light, respectively. SPV data were collected from 57 particles with different cavity sizes under 450-nm illumination.

Following this principle, we systematically varied cavity size and mapped the resulting charge separation using SPVM. In small cavities (Fig. 5a–c and Fig. S29), photogenerated electrons preferentially accumulate within the cavity. In contrast, large cavities favor hole accumulation (Fig. 5d–f and Fig. S30), demonstrating reversible control over the direction of charge separation. Figure 5g plots the dependence of cavity-induced charge separation on cavity size across tens of particles. Given the geometry of light incidence in SPVM measurements (inset of Fig. 5g), the effective cavity size is defined as *L* = *w* · sin*θ*, where *w* is the cavity width and *θ* is the glancing angle of the incident light. The data reveal that the direction of charge separation depends on *L* relative to the excitation wavelength (λ): for *L* > *λ*, electrons preferentially transfer from the cavity to the {001} surface, whereas for *L* < λ, charge separation occurs in the opposite direction. Moreover, the magnitude of charge separation increases with *L* for *L* > λ and decreases with *L* for *L* < λ. This behavior is consistent with the underlying physical mechanism, whereby light intensity within the cavity is enhanced by whispering-gallery resonances for *L* > λ and attenuated by the zero-mode waveguide effect for *L* < λ. These results demonstrate that optical-structure engineering allows precise and simultaneous control of both the magnitude and direction of photogenerated charge separation at micro- and nanoscales.

Finally, to demonstrate the functional relevance of this effect for energy conversion, we examined the cavity-induced charge separation in photocatalytic reactions. Surface potential and photovoltage measurements in solution [58,59] show that the charge separation between the cavity and {001} surface regions persists and is further enhanced under photocatalytic conditions, owing to increased surface band bending in solution (Fig. S31). Single-particle photocatalytic activity measurements using scanning electrochemical microscopy [60] reveal that lateral separation of electrons and holes can drive spatially separated redox reactions (Fig. S32). Consistently, cavity-structured Cu_2_O powders exhibit severalfold higher photocatalytic hydrogen evolution than cubic counterparts (Fig. S33), demonstrating that optically engineered charge separation directly translates into enhanced photocatalytic energy conversion.

## CONCLUSION

This work provides direct imaging evidence that optical-cavity structures generate non-uniform light-field distributions, which in turn induces efficient diffusive charge separation. More importantly, by directly visualizing this process at the nanoscale across an exceptionally wide temporal range—from femtoseconds to seconds—using TR-PEEM and modulated SPVM, we gain mechanistic insights in exquisite detail. These measurements reveal that the observed charge separation originates from ultrafast hot-electron diffusion within ∼3 ps and trap-limited electron diffusion on ∼5 ms timescales, thereby deepening the fundamental understanding of complex diffusion-driven charge-separation phenomena in semiconductors. Building on this mechanistic insight, we further demonstrate that tuning optical effects enables control over both the magnitude and direction of charge separation, offering a route to optimize photoelectric conversion through diverse photonic architectures. Notably, in the current optical structures, the enhancement factor is only in the order of tens, yet it already produces pronounced charge separation. With optical enhancement potentially increased by several orders of magnitude in more sophisticated architectures [22,24,61,62], this strategy holds substantial potential for advancing optoelectronic devices and solar energy conversion technologies. To realize this potential, future cavity design should optimize cavity size to support optical resonance (e.g. matching integer multiples of the wavelength) and tailor cavity geometry to minimize photon dispersion while sustaining well-defined modes. Material selection should balance a sufficiently large real part of the refractive index, enabling strong light-field modulation and spatial inhomogeneity, with an appropriate imaginary part to ensure efficient light absorption without excessive optical loss. Translation into viable technologies further requires improving fabrication consistency via controlled synthesis and advanced lithography, together with surface passivation to ensure structural stability under operating conditions.

## Supplementary Material

nwag327_Supplemental_File
