# Supplementary material for: Optical-cavity-driven photogenerated charge separation revealed by spatiotemporal imaging
Source: Natl Sci Rev. 2026 May 29;13(15):nwag327. doi: 10.1093/nsr/nwag327 (PMC13459972; doi:10.1093/nsr/nwag327)
Supplement: Supplementary file 1 [file nwag327_supplemental_file.pdf]

Supplementary Information for

Optical-cavity-driven photogenerated charge separation revealed by spatiotemporal imaging

Chenwei Ni[1,2], Thomas Dittrich[3], Jianbo Tang[4], Guanhua Zhang[4], Qian Li[1,2], Meng Liu[4], Fengke Sun[4], Bing Huang[1,2], Yixian Cao[1,2], Wenming Tian[4], Kaifeng Wu[4], Fengtao Fan[1], Ruotian Chen[1,*] & Can Li[1,2,*]

[1]State Key Laboratory of Catalysis, Dalian National Laboratory for Clean Energy, Dalian Institute of Chemical Physics, Chinese Academy of Sciences; Zhongshan Road 457, Dalian 116023, China.

[2]University of Chinese Academy of Sciences; 19A Yuquan Road, Beijing 100049, China.

[3]Helmholtz-Center Berlin for Materials and Energy GmbH; Kekulestr. 5, D-12489 Berlin, Germany.

[4]State Key Laboratory of Chemical Reaction Dynamics, Dalian Institute of Chemical Physics, Chinese Academy of Sciences; Zhongshan Road 457, Dalian 116023, China.

*Corresponding author. Email: rtchen@dicp.ac.cn (R.C.); canli@dicp.ac.cn (C.L.)

Methods

Electrochemical growth of Cu_2O particles. Isolated Cu_2O single crystals were prepared on fluorine-doped tin oxide (FTO) conducting substrates via electrodeposition, following a method similar to that reported previously [1,2]. All Cu_2O particles were galvanostatically electrodeposited from an aqueous solution of 0.02 M $Cu(NO_3)_2$ (Aladdin), with the pH adjusted to 5.0 prior to deposition, at a temperature of 60 °C. The deposition current density varied from 0.04 to 0.1 mA cm^{-2}, leading to a systematic morphological evolution from branched to faceted architectures, as shown in Supplementary Fig. 1. Cavity-structured Cu_2O particles were obtained within a current density range of 0.06–0.09 mA cm^{-2}, where the cavity size decreases with increasing current density (Supplementary Fig. 1). At a current density of 0.1 mA cm^{-2}, well-defined cubic Cu_2O particles were formed for comparison analysis.

Synthesis of CdS quantum dots (QDs). CdS QDs were synthesized in noncoordinating solvents following a previously reported procedure [3]. In a typical synthesis, CdO (0.077 g, Sigma-Aldrich), oleic acid (7.5 mL, Sigma-Aldrich), and 1-octadecene (ODE, 15 mL, Sigma-Aldrich) were loaded into a reaction flask, degassed under vacuum at 90 °C for 10 min, and subsequently heated to 270 °C under a N_2 atmosphere until the CdO was completely dissolved. A sulfur precursor solution (3 mL, 0.1 M sulfur in ODE) was then rapidly injected into the hot mixture. After 30 s of reaction, the growth was quenched by injecting 7 mL of room-temperature ODE and removing the heating mantle. The resulting CdS QDs were purified by centrifugation

with ethanol and finally dispersed in hexane for further use.

Synthesis of Cu_2O powders. To evaluate the photocatalytic H_2 evolution activity in a quantitatively measurable manner, single-crystal Cu_2O powders were synthesized via a hydrothermal method. In a typical procedure, a 50 mL aqueous solution containing 0.1 M $Cu(NO_3)_2$ (Aladdin), 0.1 M ethylenediaminetetraacetic acid (EDTA, Sinopharm), and 0.7 g NaOH (Sinopharm) was prepared, with the pH adjusted to 10, and then transferred into a 100 mL Teflon-lined stainless-steel autoclave. The autoclave was heated at 160 °C for 24 h to obtain Cu_2O powders consisting of both cavity-structured and cubic particles (Supplementary Fig. 33a). Increasing the reaction temperature to 180 °C for 24 h yielded exclusively cubic Cu_2O particles (Supplementary Fig. 33b).

Atomic force microscopy (AFM). All scanning probe measurements were conducted using a commercial atomic force microscopy (AFM) system (Bruker Dimension Icon). For measurements under ambient conditions, Pt/Ir-coated Si probes (Bruker SCM-PIT) with a spring constant of 1–5 N m^{-1} and a resonance frequency of 60–100 kHz were used. For in situ AFM measurements, experiments were performed in a custom-built quartz liquid cell containing 10 mM KCl solution. Force curves in liquid were acquired using PeakForce Quantitative Nanomechanical Mapping mode with a negatively charged Si probe (Scanasyst Fluid) at a ramp rate of 1 Hz. For simultaneous sensing of surface and solution potentials, a conductive Au probe (Opus 160AC-GG) was operated in direct-contact mode. The potential relative to a reference electrode was measured using a high-impedance differential amplifier integrated with a bipotentiostat (CHI 760E).

Kelvin probe force microscopy (KPFM). KPFM measurements were performed in the amplitude-modulated mode (AM-KPFM) to map the contact potential difference (CPD). The CPD is defined as the potential of the sample surface relative to that of the tip. A low AC voltage (0.5 V) was applied to the tip, with negligible influence on the measured surface potential. Lift-by-Sensor interleave mode was employed with a lift height of 20 nm to minimize crosstalk artifacts arising from tip–cantilever compensation while maintaining high-quality imaging.

Surface photovoltage microscopy (SPVM). Surface photovoltage (SPV) is defined as the photo-induced change in surface potential [4] and can be quantified by extracting the photo-induced differences in CPD from KPFM measurements, i.e., SPV = $CPD_{light} - CPD_{dark}$, where CPD_{light} and CPD_{dark} denote the CPD values measured under illuminated and dark conditions, respectively. Steady-state SPVM images were obtained by continuously mapping KPFM images under dark and illuminated conditions at the same location and extracting the differences between these images. Light illumination was provided by a 450-nm laser with an initial intensity of 80 mW cm^{-2}, attenuated using a neutral density filter, and SPVM images were typically acquired at an intensity of 10 mW cm^{-2}. For wavelength-dependent SPV measurements, the light source was replaced with a 300-W xenon arc lamp (PLS-SXE300, Beijing Perfect Light) coupled to a Zolix Omni-λ 500 monochromator to enable tunable excitation.

The experimental setup for modulated SPVM is schematically illustrated in Supplementary Fig. 28a. In the modulated regime, a chopper generated frequency-

modulated light, which was then split by a beam splitter. One portion of the light was directed to a photodetector, converting the modulated light into square-wave reference signals for SPV measurement. The other portion was focused on the Cu_2O particles for modulated photoexcitation. AM-KPFM recorded the fluctuations of CPD signals under modulated illumination in real time. The modulated CPD signals and the square-wave reference signals were synchronously fed into a lock-in amplifier (SR830 DSP), which outputs the amplitude and phase of the modulated SPV signals. To map the modulated SPV signals (e.g., the phase image shown in Fig. 4d), the resulting amplitude and phase signals were input into the AFM image channels during tip scanning. A more detailed experimental setup for SPVM is described in our recent publication [5].

Photoluminescence (PL) imaging microscopy. PL imaging was performed using a scanning confocal microscope (IX73, Olympus) coupled with time-correlated single-photon counting (TCSPC) and a 100× air objective lens (NA 0.95, Olympus PLFLN 100X) in wide-field illumination mode at room temperature. A 405-nm pulsed laser (PIXEA-405, Aurea Technology) with a 45-ps pulse width and a 120-MHz repetition rate was used for excitation. The laser beam was focused to a spot of ~20 μm in diameter to ensure uniform illumination across the entire Cu_2O particle. Emission was filtered through a 430–490 nm or 550–600 nm bandpass filter, after which the PL signal was collected in a dark-field imaging configuration using a high-speed detector (HPM-100-40, Hamamatsu). PL images were obtained by rapid scanning of the galvanometer mirror, with each image consisting of 256 × 256 pixels and a dwell time of ~1 ms per pixel, allowing simultaneous acquisition of PL decay kinetics. The PL spectra were

collected using a monochromator (SpectraPro-HRS-300, Princeton Instruments) coupled to a CCD camera (PIXIS 100, Princeton Instruments). To establish the relationship between excitation intensity and PL intensity, the laser power density was adjusted using a neutral density filter and measured with a power meter (PM100D S130VC, Thorlabs).

Optical simulations. Optical simulations were performed using COMSOL Multiphysics 5.6. Electric-field distributions around the cavity structures were calculated in the frequency domain using the refractive index (n = 3.385) and extinction coefficient (k = 1.0195) of Cu_2O at an excitation wavelength of 450 nm [6]. The simulation model is shown in Supplementary Fig. 18a. Scattering boundary conditions were applied at the outer boundaries of the computational domain to approximate an open system, allowing outgoing electromagnetic waves to exit the simulation region with minimal artificial reflection. This approach enables accurate modeling of the optical response at the air/Cu_2O interface, including reflection, transmission, and absorption processes, while minimizing non-physical boundary effects. Q values were calculated from the eigenfrequency analyses (Supplementary Fig. 19), in which the finite element method was used to determine eigenmodes with associated eigenfrequencies around the frequency where the real part of the permittivity is near zero [7]. The numerical solver computed the Q as the ratio of the energies stored and dissipated via the integration of the electromagnetic field intensities.

Time-resolved photoemission electron microscopy (TR-PEEM). TRPEEM combines femtosecond pump–probe technique with photoemission electron

microscopy (PEEM) to map charge dynamics on ultrafast and ultrasmall scales [8]. The femtosecond laser system was based on an oscillator (FLINT, Light Conversion) delivering 6 W pulses with <100 fs pulse width, a center wavelength of 1030 nm, and a repetition rate of 76 MHz. The pulses were focused into a lithium triborate (LBO) crystal to generate second-harmonic 515-nm (2.4 eV) pulses, which served as the pump for photoexcitation. The 2.4-eV pulses were subsequently frequency-doubled to 4.8 eV using a beta barium borate (BBO) crystal, providing the time-delayed probe pulses. The 4.8-eV probe selectively emitted electrons from the conduction band at the Γ point or from defect states near the conduction band of Cu_2O, based on its band structure [9]. Pump and probe beams were collimated using a customized dichroic mirror and focused onto the sample in the PEEM chamber at a grazing angle of ~16°, producing laser spots of ~50 × 100 μm^2 for the pump and ~100 × 125 μm^2 for the probe. The pump fluence was 15 $\mu J\ cm^{-2}$, corresponding to an excitation density of $2.6 \times 10^{17}\ cm^{-3}$, calculated using an absorption coefficient of 13,000 cm^{-1} for Cu_2O [6]. Photoemitted electrons were mapped with an aberration-corrected spectroscopic photoemission and low-energy electron microscopy system (SPELEEM III, ELMITEC). TR-PEEM images were acquired in the imaging plane, and time-resolved photoemission spectroscopy (TR-PES) was taken from the dispersive plane at different pump–probe delay times. Prior to TR-PES measurements, an area-selected aperture was used to limit the detection area to a circular region of 1.5 μm diameter, enabling selective collection from cavity or {001} surface regions of a single Cu_2O particle.

Spatially resolved X-ray spectroscopy. Spatially resolved X-ray spectroscopy was

performed by combining synchrotron radiation X-rays with PEEM. Soft X-rays were delivered from beamline BL09U at the Shanghai Synchrotron Radiation Facility (SSRF). Photoemission was carried out using X-rays with a photon energy of 1050 eV, and the emitted electrons were mapped by PEEM with an inserted energy slit. By scanning the kinetic energy of the photoemitted electrons, energy-resolved XPEEM images were recorded as a stack, from which X-ray photoelectron spectroscopy (XPS) spectra for cavity and {001} surface regions were extracted. X-ray absorption imaging was performed by varying the incident photon energy across the absorption edges of interest (Cu L edge) and detecting secondary electrons with a kinetic energy of 1.5 eV. Spatially resolved X-ray absorption spectra were then obtained from the stacked X-ray absorption images.

High-resolution transmission electron microscopy (HRTEM) and electron energy loss spectroscopy (EELS). HRTEM and EELS were carried out on a JEOL JEM-ARM300F microscope operated at 300 kV. High-resolution images were acquired in scanning mode at a magnification of 600000×. EELS spectra were collected with a 41.7 mrad collection semi-angle and a 5 mm entrance aperture. The exposure time was 0.1 ms per frame, with 15 frames summed per spectrum, yielding a total acquisition time of 1.5 ms. All spectra were acquired after gain normalization and high-quality dark correction.

Scanning electron microscopy (SEM). SEM images were taken using a Quanta 200 FEG scanning electron microscope at an operating voltage of 30 kV.

Transient SPV (TPV) spectroscopy. TPV measurements were performed in a fixed-

capacitor arrangement [10,11], in which the time evolution of the photovoltage in an RC circuit was recorded via a high-impedance buffer with a measurement resistance of 50 GΩ. SPV transients were excited by pulses from a tunable neodymium-doped yttrium aluminium garnet (Nd:YAG) laser (EKSPLA NT342/1/UVE) with a pulse duration of 4 ns and a repetition rate of 1 Hz. The transients were detected using a sampling oscilloscope (GAGE CS14200) with a temporal resolution of 5 ns.

Scanning electrochemical microscopy (SECM). SECM was used to evaluate charge-driven reactions at the single-particle level. Au microelectrodes were fabricated by sealing 25-µm Au wires in borosilicate glass capillaries using a laser-based micropipette puller (P-2000, Sutter Instrument) and polishing the tip under microscopic control. The tip diameter (~1.3 µm) was estimated from the diffusion-limiting current (i_d) of 1 mM ferrocenemethanol (FcMeOH) in 0.1 M KCl using cyclic voltammetry (Supplementary Fig. 32a) and the relation [12]:

$$i_d = 4nFD_0C_0\text{a}, \quad (1)$$

where n = 1, F is the Faraday constant, $D_0 = 7.8\times10^{-6}$ cm^2 s^{-1} [13], and C_0 = 1 mM. This spatial resolution allows distinction between cavity and {001} surface regions of Cu_2O particles.

SECM measurements were carried out in 0.1 M KCl solution containing 1 mM FcMeOH redox mediator using a CHI-920D bipotentiostat (CH Instrument) and a three-electrode cell configuration, where the fabricated Au microelectrode, a Pt wire, and an Ag/AgCl electrode served as a working electrode, counter electrode, and reference electrode, respectively. Cu_2O single particles on FTO served as the unbiased

substrate, and a 450-nm laser was used for illumination during measurements. The tip was biased at 0.45 V versus Ag/AgCl to trigger FcMeOH oxidation. SECM images were acquired at a constant tip–substrate height, with fast scanning used to locate single particles, followed by fine scanning to resolve the spatial distribution of redox activity.

Photocatalytic hydrogen production. Photocatalytic reactions were carried out in a Pyrex top-irradiation reaction vessel connected to a closed gas-circulation system. Cu_2O powders (50 mg) were dispersed in 100 mL of 0.5 M Na_2SO_3 aqueous solution, which served as an effective hole scavenger for photocatalytic hydrogen evolution. Prior to irradiation, the system was evacuated to remove air completely and then irradiated from the top using a 300-W Xe lamp (Ushio-CERMAX LX300). Cooling water was circulated to maintain the reaction suspension at 288 K. The evolved H_2 was analyzed online by gas chromatography (Shimadzu GC-8A) equipped with a thermal conductivity detector (TCD) using Ar as the carrier gas.

Supplementary Notes

Supplementary Note 1. Determining the cavity quality factor from spatially resolved PL lifetime measurements. Time-resolved PL reveals that the PL lifetime of QDs on the {001} surface (τ_0) is ~3.4 ns, whereas that within the cavity (τ_{cav}) is ~1.5 ns. This yields the Purcell factor (F_P):

$$F_{\mathrm{P}} = \frac{\tau_0}{\tau_{\mathrm{cav}}} \approx 2.3. \tag{2}$$

Under the weak-coupling regime and for a dielectric resonator, the Purcell factor is related to the cavity quality factor (Q) and the effective optical mode volume V_{eff} as:

$$F_{\mathrm{P}} = \frac{3}{4\pi^2}\left(\frac{\lambda}{n}\right)^3 \frac{Q}{V_{\mathrm{eff}}}, \tag{3}$$

where λ is the free-space wavelength of the emitter, and n is the refractive index of the cavity medium. V_{eff} is defined as:

$$V_{eff} = \frac{\int \varepsilon(\boldsymbol{r})|E(\boldsymbol{r})|^2\, d\boldsymbol{r}}{max[\varepsilon(\boldsymbol{r})|E(\boldsymbol{r})|^2]}, \tag{4}$$

where $\varepsilon(\mathbf{r})$ is the spatially dependent dielectric function and $\mathrm{E}(\mathbf{r})$ is the electric field distribution of the cavity mode. This definition describes the effective spatial extent over which the electromagnetic energy is localized within the cavity. Physically, V_{eff} characterizes the degree of optical field confinement: a smaller V_{eff} indicates stronger field localization and higher energy density, thereby enhancing light–matter interactions. Using λ = 450 nm, n = 3.38 [6], F_{P} = 2.3, and V_{eff} obtained from COMSOL simulations (Supplementary Fig. 17), the cavity quality factor was estimated to be Q ≈ 27.

Supplementary Note 2. Fitting the time evolution of diffusive SPV. The temporal evolution of diffusive SPV resulting from lateral charge separation between cavity and {001} surface regions was modeled using a one-dimensional framework based on coupled Poisson and continuity equations. Integration of the Poisson equation yields the following expression for the time-dependent SPV [14]:

$$SPV(t) = \frac{e}{\varepsilon\varepsilon_0}\int_0^L dx \int_0^x n(y,t)dy, \tag{5}$$

where ε_0 is the vacuum dielectric constant and ε = 7.2 is the relative dielectric constant of Cu_2O [15]. The time-dependent charge distribution n(x, t) is described by the

continuity equation:

$$\frac{\partial n(x,t)}{\partial t} = D\frac{\partial^2 n(x,t)}{\partial x^2}, \tag{6}$$

where drift and recombination processes are neglected. The boundary conditions were set according to the morphology of the cavity-structured Cu_2O particle (particle size ~6 μm, cavity size ~2 μm). The data were fitted using a unipolar diffusion regime, in which hot electrons and holes diffuse from the cavity to the {001} surface regions on timescales of several picoseconds and hundreds of picoseconds, respectively. For hot-electron diffusion, the diffusivity (D) decays exponentially due to hot electron relaxation and is expressed as [16]:

$$D(t) = D^{*}\mathrm{e}^{-\frac{t}{\tau}} + D_0, \tag{7}$$

where D* is the diffusivity of the initial non-equilibrium hot electrons, D_0 is the diffusivity of electrons at equilibrium, and τ is the time constant of hot electron relaxation. For hole diffusion, the hole diffusivity (D_h) is treated as a constant. The fitting results are shown in Supplementary Fig. 27.

Supplementary Note 3. Quantitative relationship between steady-state diffusive SPV and diffusivity asymmetry. The electron and hole currents can be expressed by the drift-diffusion equation:

$$J_n = e\mu_n nE(x) + eD_n\left(\frac{\mathrm{d}n}{\mathrm{d}x}\right), \tag{8}$$

$$J_p = e\mu_p pE(x) - eD_p\left(\frac{\mathrm{d}p}{\mathrm{d}x}\right), \tag{9}$$

where n and p denote the electron and hole concentrations, μ_n and μ_p are the corresponding mobilities, D_n and D_p are the diffusion coefficients, E(x) is the electric field, and e is the elementary charge. Under steady-state illumination conditions, the

total current vanishes, and the quasi-neutrality approximation is adopted, i.e.,

$$J_n + J_p = 0; \frac{\mathrm{d}\delta n}{\mathrm{d}x} \approx \frac{\mathrm{d}\delta p}{\mathrm{d}x}; \delta n \approx \delta p, \tag{10}$$

where δn and δp denote the photogenerated carrier densities. The electric field arising from asymmetric diffusion is then given by:

$$E(x) = -\left(\frac{D_n - D_p}{\mu_n n + \mu_p p}\right) \cdot \frac{\mathrm{d}\delta n}{\mathrm{d}x}. \tag{11}$$

Integrating Eq. (11) under semi-infinite boundary conditions and using the Einstein relation, the SPV arising from diffusivity asymmetry can be obtained:

$$SPV = \frac{kT}{e}\frac{D_n - D_p}{D_n + D_p} In\left[1 + \frac{(D_n + D_p)\delta n(0)}{(n_0 D_n + p_0 D_p)}\right], \tag{12}$$

where $\delta n(0)$ is the photogenerated carrier density at the surface, n_0 and p_0 are the intrinsic carrier densities in the dark.

Supplementary Note 4. Functional relationship between phase and time constant of SPV in the modulated SPV regime. The generation and decay of the SPV, resulting from charge separation and recombination, can be described by exponential functions:

$$SPV(t) = \begin{cases} SPV_0\left(1 - \mathrm{e}^{-\frac{t}{\tau}}\right), & SPV\ generation \\ SPV_0 \cdot \mathrm{e}^{-\frac{t}{\tau}}, & SPV\ decay \end{cases} \tag{13}$$

where SPV_0 is the SPV value in equilibrium and τ is the time constant of SPV dynamics (Supplementary Fig. 28b). Under chopped light illumination, the SPV evolution over time is shown in Supplementary Fig. 28c and is given by:

$$SPV_n = SPV_0\left(1 - \mathrm{e}^{-\frac{t_0}{\tau}}\right), \tag{14}$$

where SPV_n is the SPV value after n pulses, and t_0 is the corresponding time. After n + 1 pulses, the SPV signals become:

$$SPV_{n+1} = SPV_0\left(1 - \mathrm{e}^{-\frac{t_0+0.5T}{\tau}}\right)\mathrm{e}^{-0.5\frac{T}{\tau}} = \mathrm{e}^{-\frac{T}{\tau}} \cdot SPV_n + \left(\mathrm{e}^{-0.5\frac{T}{\tau}} - \mathrm{e}^{-\frac{T}{\tau}}\right) \cdot SPV_0, \quad (15)$$

where T is the light modulation period. The first-order linear recurrence relation gives:

$$SPV_n = SPV_0 \cdot \frac{\mathrm{e}^{-0.5\frac{T}{\tau}} - \mathrm{e}^{-\frac{T}{\tau}}}{\mathrm{e}^{-\frac{T}{\tau}} - 1} \cdot \left(\mathrm{e}^{-n\frac{T}{\tau}} - 1\right). \quad (16)$$

When the SPV amplitude stabilizes under pulsed light (nT >> τ), the SPV evolution over a single modulation cycle is:

$$SPV(t) = \begin{cases} SPV_0\left(1 - \dfrac{\mathrm{e}^{-\frac{T}{2\tau}} - 1}{\mathrm{e}^{-\frac{T}{\tau}} - 1} \cdot \mathrm{e}^{-\frac{t}{\tau}}\right), 0 < t < \dfrac{T}{2} \\ SPV_0 \cdot \dfrac{\mathrm{e}^{-\frac{T}{2\tau}} - 1}{\mathrm{e}^{-\frac{T}{\tau}} - 1} \cdot \mathrm{e}^{\frac{T}{2\tau}} \cdot \mathrm{e}^{-\frac{t}{\tau}}, \dfrac{T}{2} < t < T \end{cases}. \quad (17)$$

This expression can also be represented as a Fourier series:

$$SPV(t) = \sum_{i=0}^{\infty}\left[X_i \sin\left(\frac{2\pi i}{T}t\right) + Y_i \,\mathrm{con}\left(\frac{2\pi i}{T}t\right)\right] = \sum_{i=0}^{\infty} R_i \sin\left(\frac{2\pi i}{T}t + \theta_i\right), \quad (18)$$

where X_i, Y_i, R_i, and θ_i represent the real parts, imaginary parts, amplitude, and phase of the modulated SPV, respectively, and i is the Fourier series index. After inputting SPV(t) into a Lock-in amplifier, the Fourier components are obtained as:

$$X_i = \frac{2}{T}\int_0^T SPV(t)\sin\left(\frac{2\pi i}{T}t\right) \cdot \mathrm{d}t\,; \quad (19)$$

$$Y_i = \frac{2}{T}\int_0^T SPV(t)\cos\left(\frac{2\pi i}{T}t\right) \cdot \mathrm{d}t. \quad (20)$$

Combining these equations, the lock-in output signals are:

$$X_i = -\,2SPV_0 \cdot \frac{(\cos i\pi - 1)}{(2\pi i)^2 + \left(\frac{T}{\tau}\right)^2} \cdot \frac{\left(\frac{T}{\tau}\right)^2}{2\pi i}; \quad (21)$$

$$Y_i = 2SPV_0 \cdot \frac{(\cos i\pi - 1)}{(2\pi i)^2 + \left(\frac{T}{\tau}\right)^2} \cdot \frac{T}{\tau}; \quad (22)$$

$$\theta_i = \arctan\left(\frac{2\pi i}{\frac{T}{\tau}}\right); \tag{23}$$

$$R_i = \sqrt{X_i^2 + Y_i^2} = -2SPV_0 \cdot \frac{(\cos i\pi - 1)}{(2\pi i)^2 + \left(\frac{T}{\tau}\right)^2} \cdot \frac{T}{\tau} \sqrt{1 + \left(\frac{\frac{T}{\tau}}{2\pi i}\right)^2}. \tag{24}$$

For multiple independent charge separation processes, the SPV signals are expressed as:

$$SPV(t) = \begin{cases} \sum_j SPV_j \left(1 - \frac{e^{-\frac{T}{2\tau_j}} - 1}{e^{-\frac{T}{\tau_j}} - 1} \cdot e^{-\frac{t}{\tau_j}}\right), 0 < t < \frac{T}{2} \\ \sum_j SPV_j \cdot \frac{e^{-\frac{T}{2\tau_j}} - 1}{e^{-\frac{T}{\tau_j}} - 1} \cdot e^{\frac{T}{2\tau_j}} \cdot e^{-\frac{t}{\tau_j}}, \frac{T}{2} < t < T \end{cases}, \tag{25}$$

where j indexes the number of independent processes. The corresponding phase signals are given by:

$$\theta_i = \arctan\left(\frac{\sum_j \left[\frac{SPV_j}{(2\pi i)^2 + \left(\frac{T}{\tau_j}\right)^2} \cdot \frac{T}{\tau_j}\right]}{\sum_j \left[\frac{SPV_j}{(2\pi i)^2 + \left(\frac{T}{\tau_j}\right)^2} \cdot \frac{\left(\frac{T}{\tau_j}\right)^2}{2\pi i}\right]}\right). \tag{26}$$

The multiple processes can be decoupled by measuring the phase at different modulation periods. In our measurements, we used i = 1, which offers the highest sensitivity. In this case, the time constant of SPV for a given process is:

$$\tau = \frac{T \cdot tan\theta}{2\pi}. \tag{27}$$

The time resolution of conventional SPVM is limited to ~1 ms owing to the KPFM feedback loop. With the lock-in amplifier (SR830) phase resolution of 0.01°, the detected SPV time precision improves to:

$$\tau = \frac{10^{-3} \times \tan 0.01^{\circ}}{2\pi} s = 2.8 \times 10^{-8}\ s. \qquad (28)$$

Thus, the phase-based time function enhances the temporal resolution of SPVM by ~5 orders of magnitude, representing a significant advancement toward time-resolved scanning probe-based techniques.

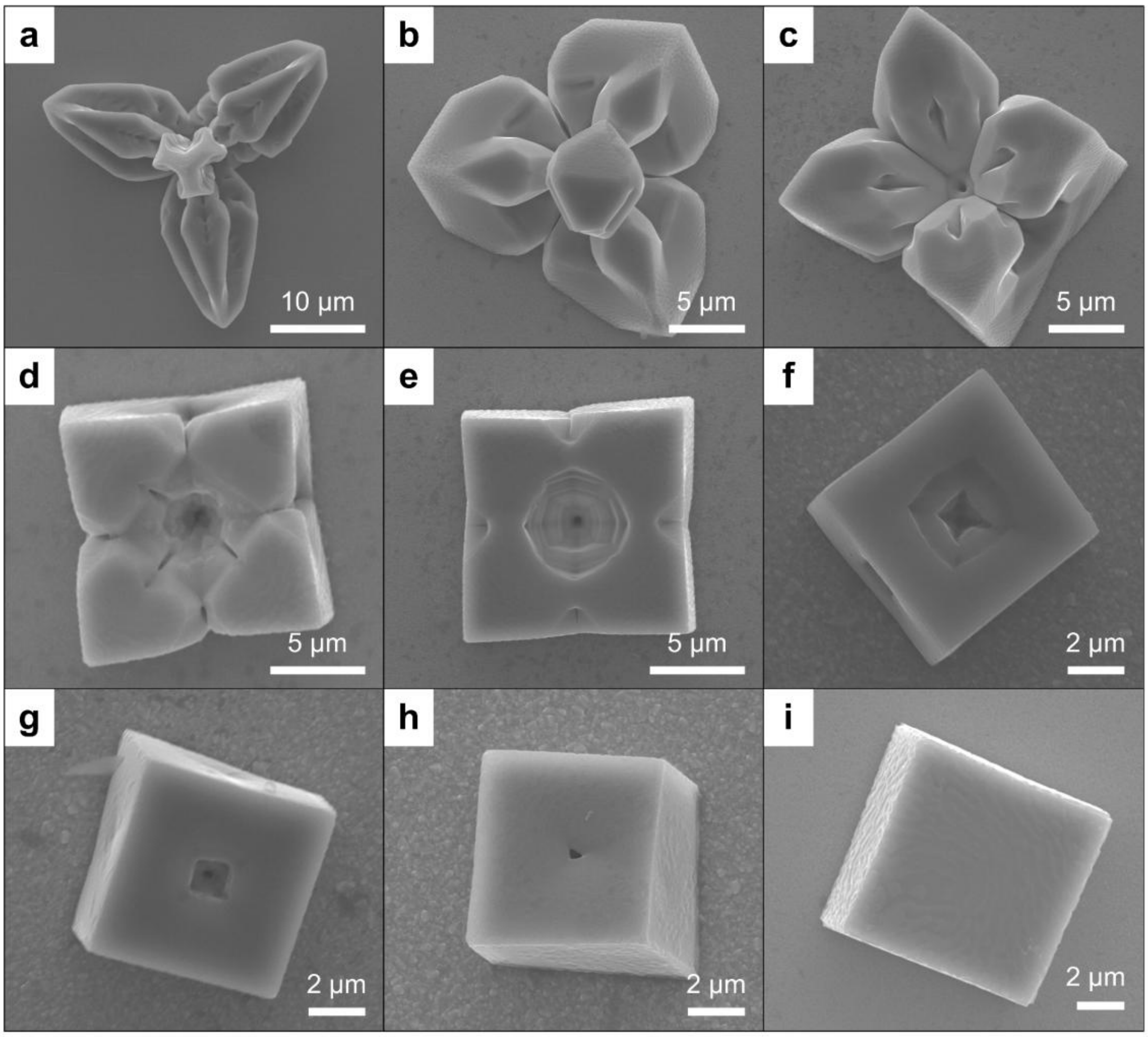

Supplementary Figure 1. SEM images showing the morphological evolution of single Cu_2O particles from branched to faceted crystals. Cu_2O particles in **a–i** were synthesized using galvanostatic electrodeposition, with the deposition current density gradually increasing from 0.04 to 0.1 mA cm^{-2}. The degree of branching decreases with increasing current, due to a more pronounced filling-in process that seals the gaps between branches. This phenomenon and its origin have been discussed previously [1,2]. The well-controlled structures establish Cu_2O as a model system for investigating cavity-induced charge separation processes.

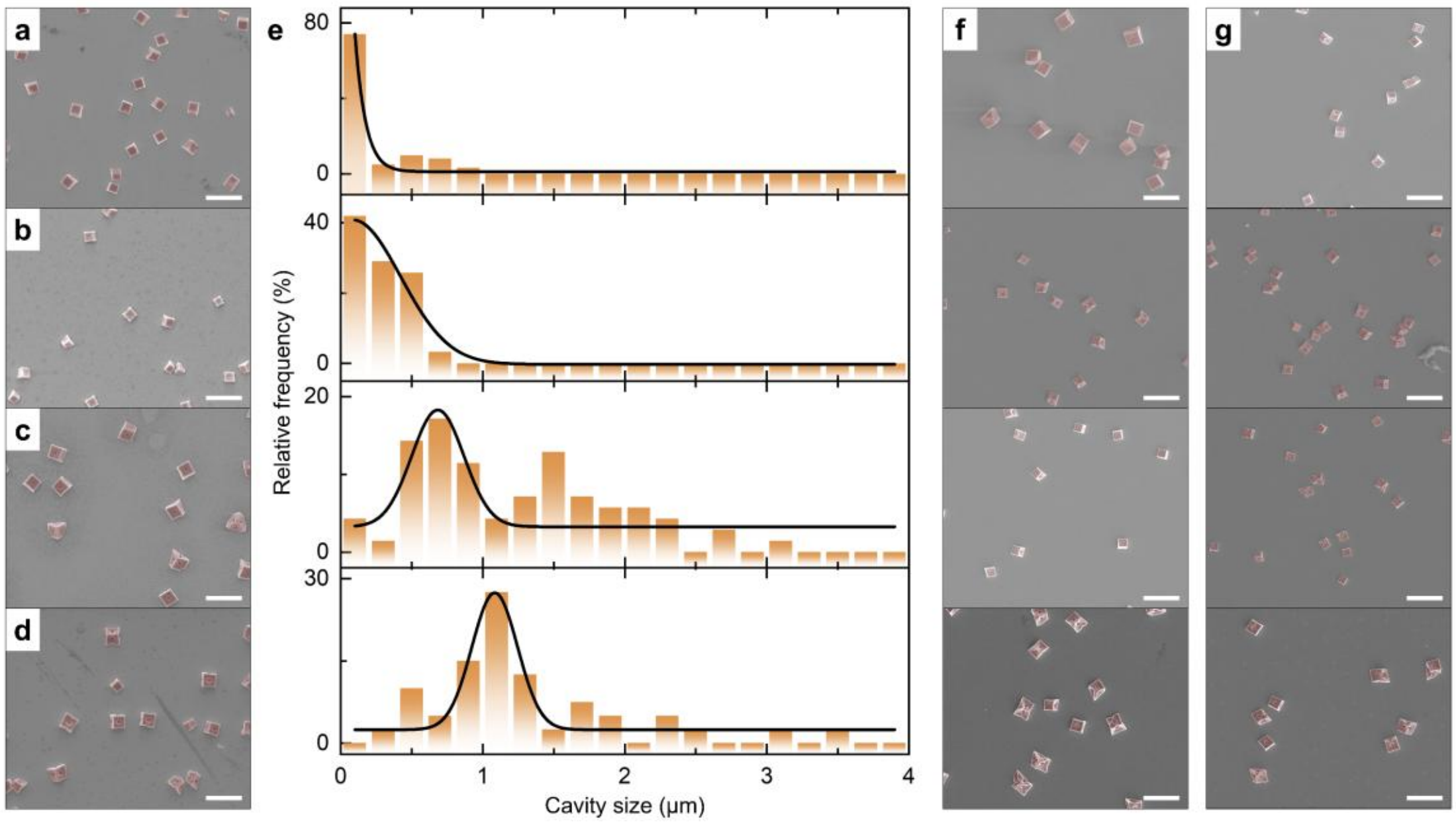

Supplementary Figure 2. Tuning of cavity size. a–d, SEM images showing cavity-structured Cu_2O particles with increasing cavity sizes from **a** to **d**. Scale bars, 20 μm. The increase of cavity size in **a–d** corresponds to the decrease of deposition current density from 0.09 to 0.06 mA cm^{-2}. **e**, Statistical analysis of cavity sizes from SEM images. The controllable tuning of cavity size allows investigation of its effect on, and control over, cavity-induced charge separation processes. **f**,**g**, SEM images from two additional independent batches of cavity-structured Cu_2O particles synthesized at current densities of 0.06–0.09 mA cm^{-2}, showing consistent morphology evolution with deposition current density. Scale bars, 20 μm. The morphological consistency under identical growth conditions confirms the good reproducibility of the cavity formation process and the reliable control of cavity size.

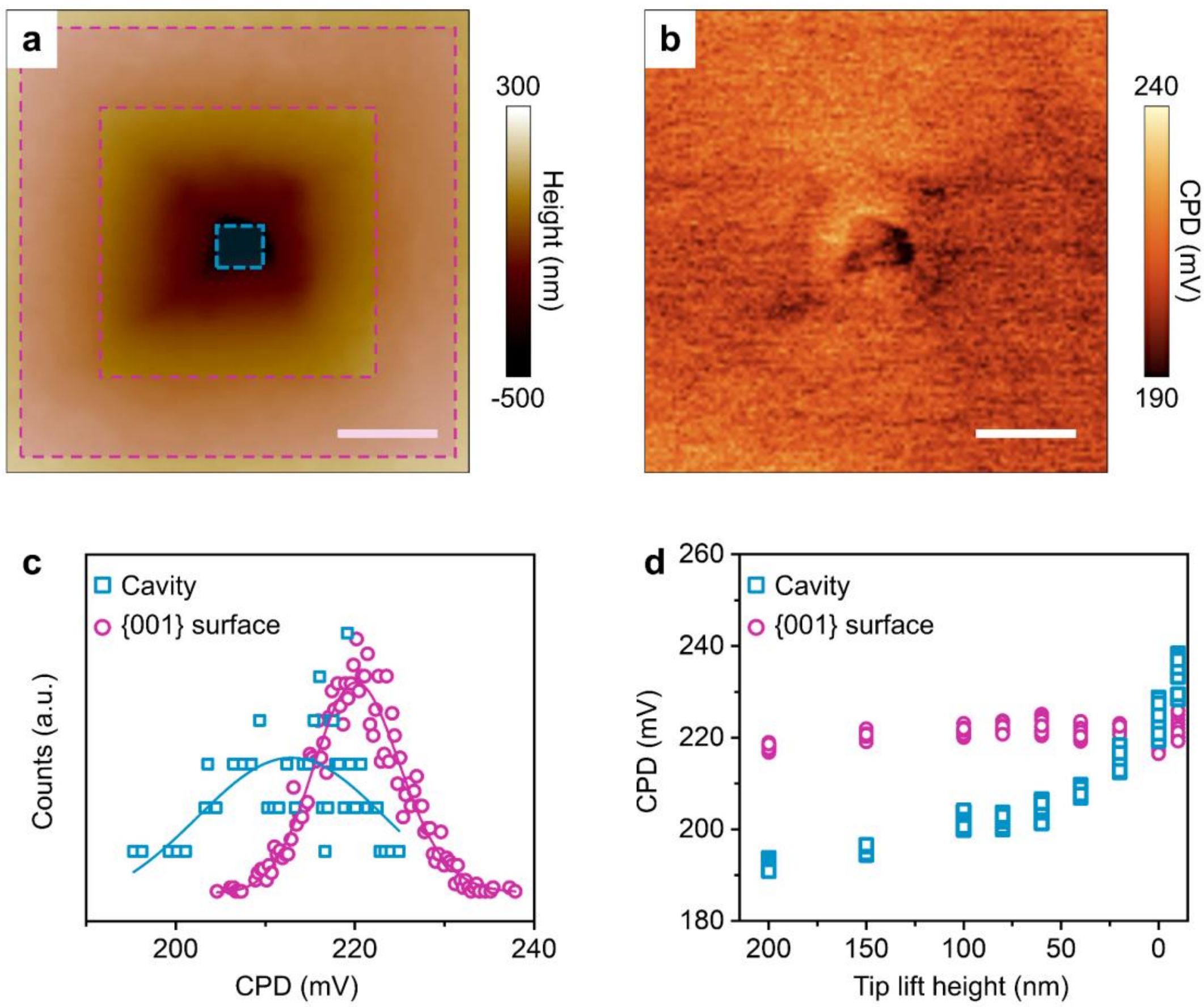

Supplementary Figure 3. KPFM measurements at finer scale and lower tip lift height. **a**,**b**, AFM (**a**) and KPFM (**b**) images of a local cavity region surrounded by {001} surface regions, recorded at a tip lift height of 20 nm. Scale bars, 1 µm. The cyan square in **a** indicates the cavity region, and the surrounding magenta area corresponds to the {001} surface. **c**, Statistic CPD signals extracted from pixels in the cavity and surrounding {001} surface regions, as marked in **a**. **d**, Statistically averaged CPD signals from the cavity and {001} surface regions plotted as a function of tip lift height.

KPFM measurements were performed in a smaller region and at low tip lift height to minimize errors arising from morphological cross-talk [17], which originates from tip and cantilever compensation and becomes negligible at reduced lift heights. The dependence of CPD on tip lift height (Supplementary Fig. 3d) shows that the CPD on

the {001} surface is largely insensitive to morphological crosstalk due to the perpendicular tip–surface geometry. In contrast, the CPD in the cavity region increases by ~20 mV as the tip lift height decreases from 100 nm to ~0 nm, attributable to topographic crosstalk. There is a trade-off between topographic crosstalk and signal-to-noise ratio when selecting the tip lift height for CPD imaging: lower tip lift heights reduce crosstalk but may increase noise. To balance these factors, CPD mapping was performed at a tip lift height of 20 nm, where high image quality is achieved while artifacts arising from crosstalk are limited to less than ~5 mV (Supplementary Fig. 3c). At a tip lift height of ~0 nm, where morphological crosstalk is minimized, the CPD values of the cavity and {001} regions are nearly identical. This observation rules out any significant built-in potential between the lateral edges or facets within the cavity and the flat {001} surface. Since the maximum steady-state SPV driven by a built-in electric field is limited by the magnitude of the built-in potential, the negligible intrinsic potential difference between the cavity and {001} regions cannot account for the observed ~60 mV SPV arising from charge separation between these regions.

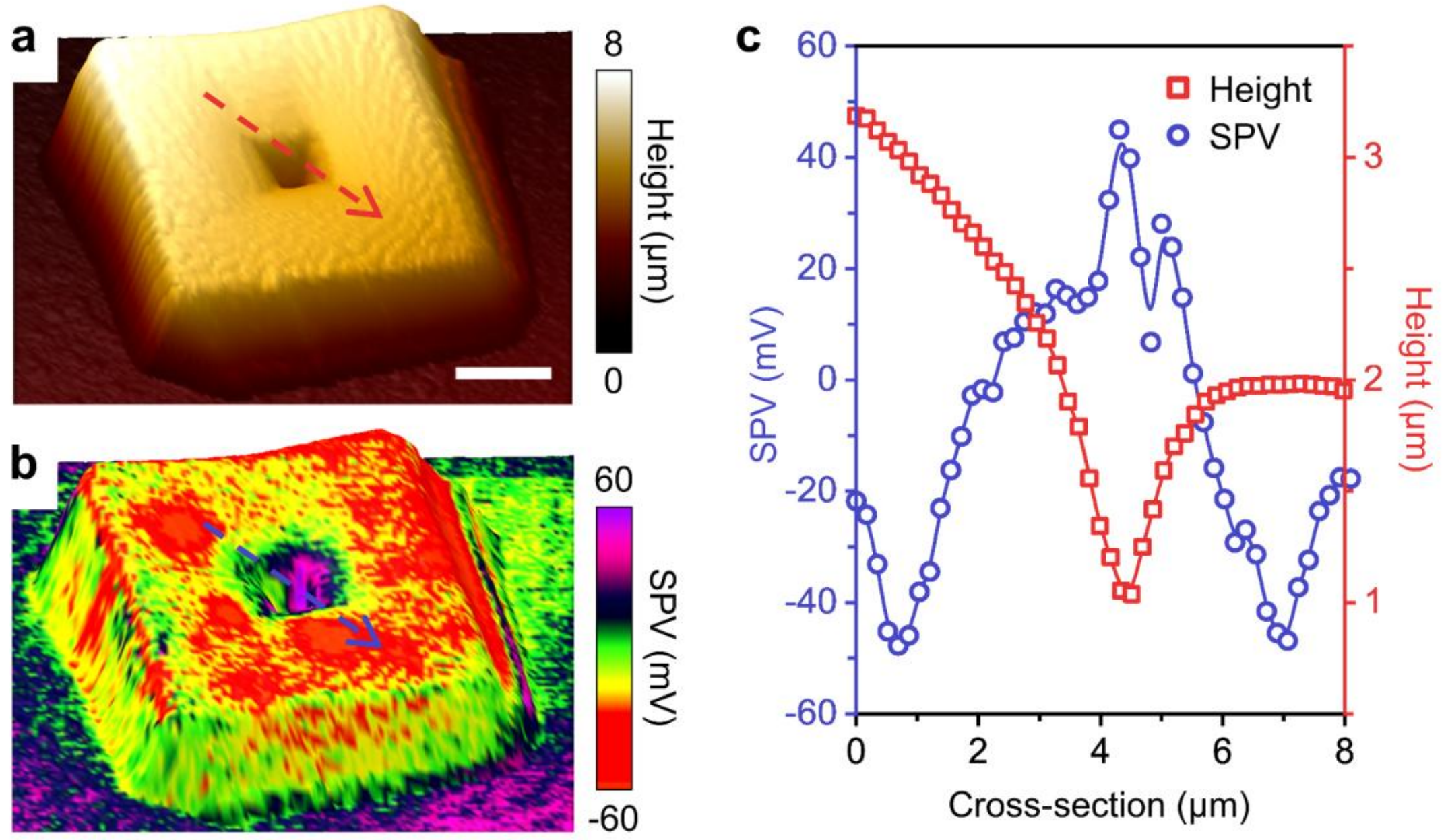

Supplementary Figure 4. Reproduction of cavity-induced charge separation. a,**b**, AFM (**a**) and SPVM (**b**) images of another cavity-structured Cu_2O particle. Scale bar, 2 μm. **c**, Line profiles extracted along the dashed lines in **a** and **b**. These data reproduce the cavity-induced charge separation observed in Fig. 1.

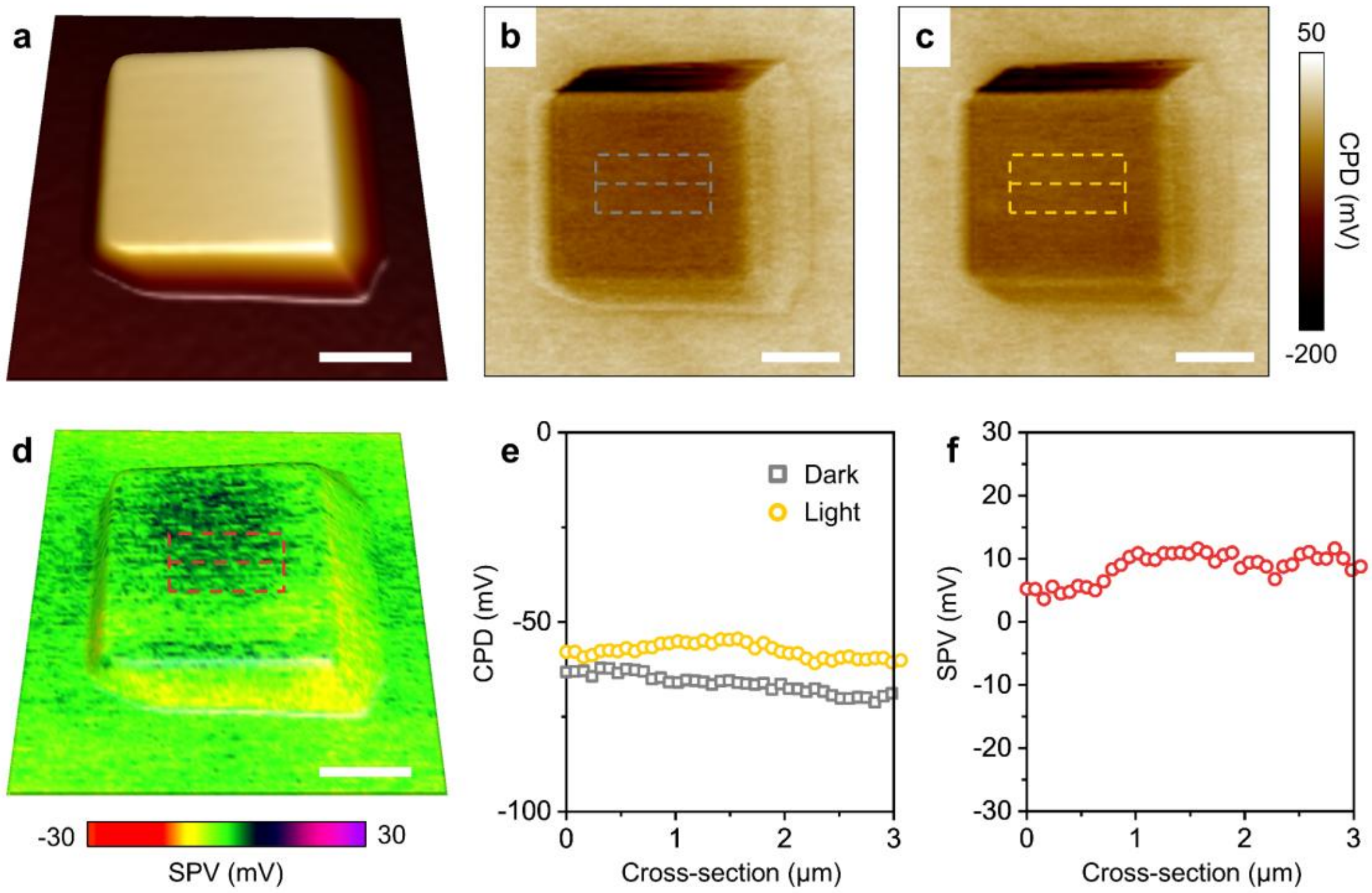

Supplementary Figure 5. KPFM and SPVM data of cubic Cu_2O. a–d, AFM image (**a**), KPFM images in dark (**b**) and under 450-nm light illumination (**c**), and SPVM image (**d**) of a cubic Cu_2O particle. Scale bars, 2 μm. **e**, CPD values extracted across dashed boxes in **b** and **c**. **f**, SPV values extracted across dashed box in **d**.

The data show that both CPD and SPV distributions are approximately uniform across the surface of cubic Cu_2O, with no observable charge separation between the center and surrounding regions. Comparison between cubic and cavity-structured Cu_2O indicates that the lateral charge separation between the cavity and {001} surface regions is induced by the cavity structures.

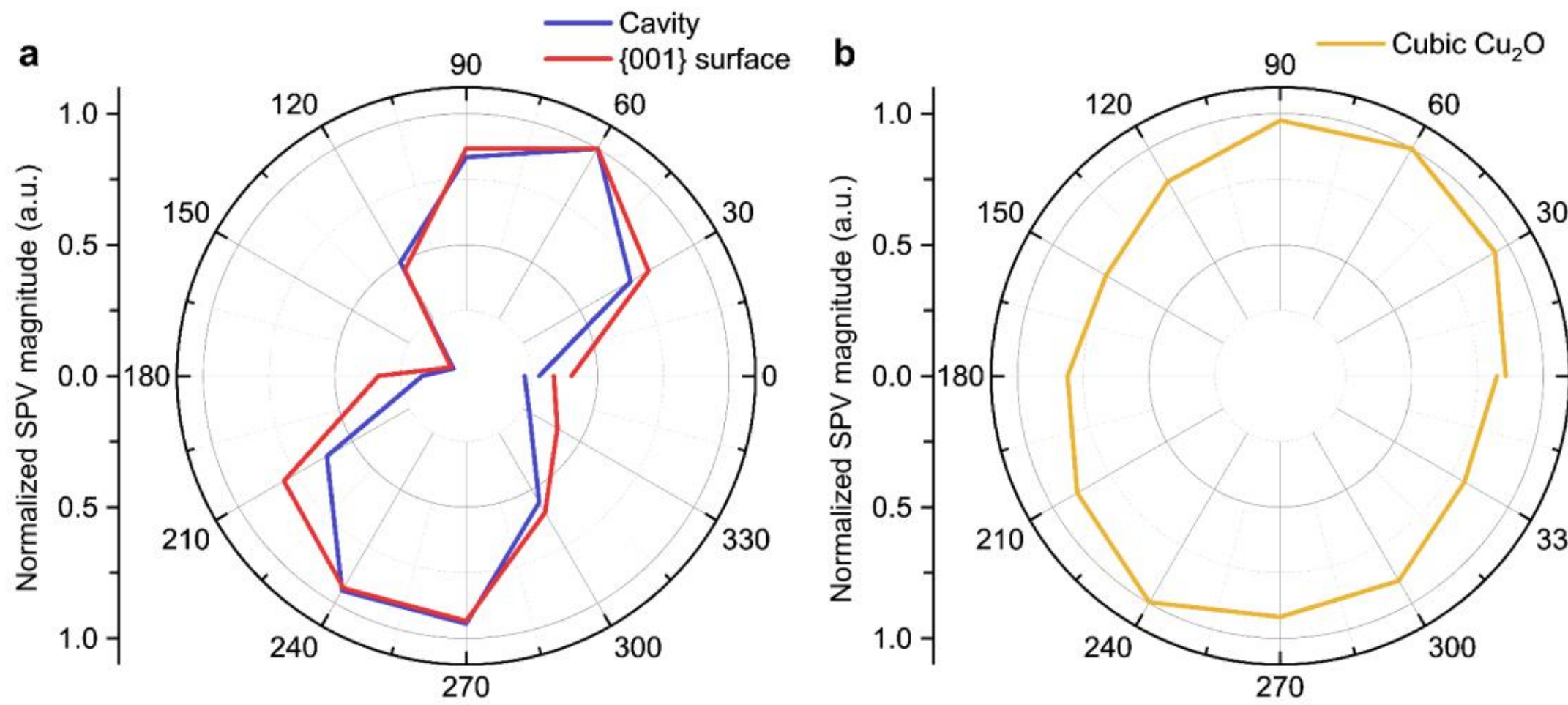

Supplementary Figure 6. Effect of light polarization on SPV signals. a,**b**, Polar plot of normalized SPV signals at different light polarization angles recorded on cavity-structured Cu_2O (**a**) and cubic Cu_2O (**b**) particles. The polarized light was generated using a rotatable linear polarizer placed in the path of a 450-nm laser beam.

The comparative results show that charge separation between the cavity and {001} surface regions exhibits a strong dependence on the light polarization angle, in clear contrast to the polarization-insensitive charge separation behavior observed in cubic Cu_2O particles. Given the vectorial nature of the optical field [18], these results indicate that the cavity-induced charge separation predominantly originates from optical resonant effects. Specifically, in the large cavity structures studied here, the optical resonant enhancement is dominated by whispering-gallery-like modes that confine light primarily in the lateral (x–y) plane, with the electric field predominantly oriented along the z direction. During SPV measurements, the illumination impinges on the sample at an oblique angle; therefore, the projection of the incident electric field along the z direction varies with the polarization angle. Only when the polarization maximizes the field component along the z direction is the resonant enhancement strongest, resulting

in a pronounced polarization dependence of the SPV. In contrast, for cubic Cu_2O particles, the SPV is mainly governed by charge separation within the space-charge region rather than optical resonant effects. In this case, the SPV primarily depends on light intensity and is largely insensitive to light polarization.

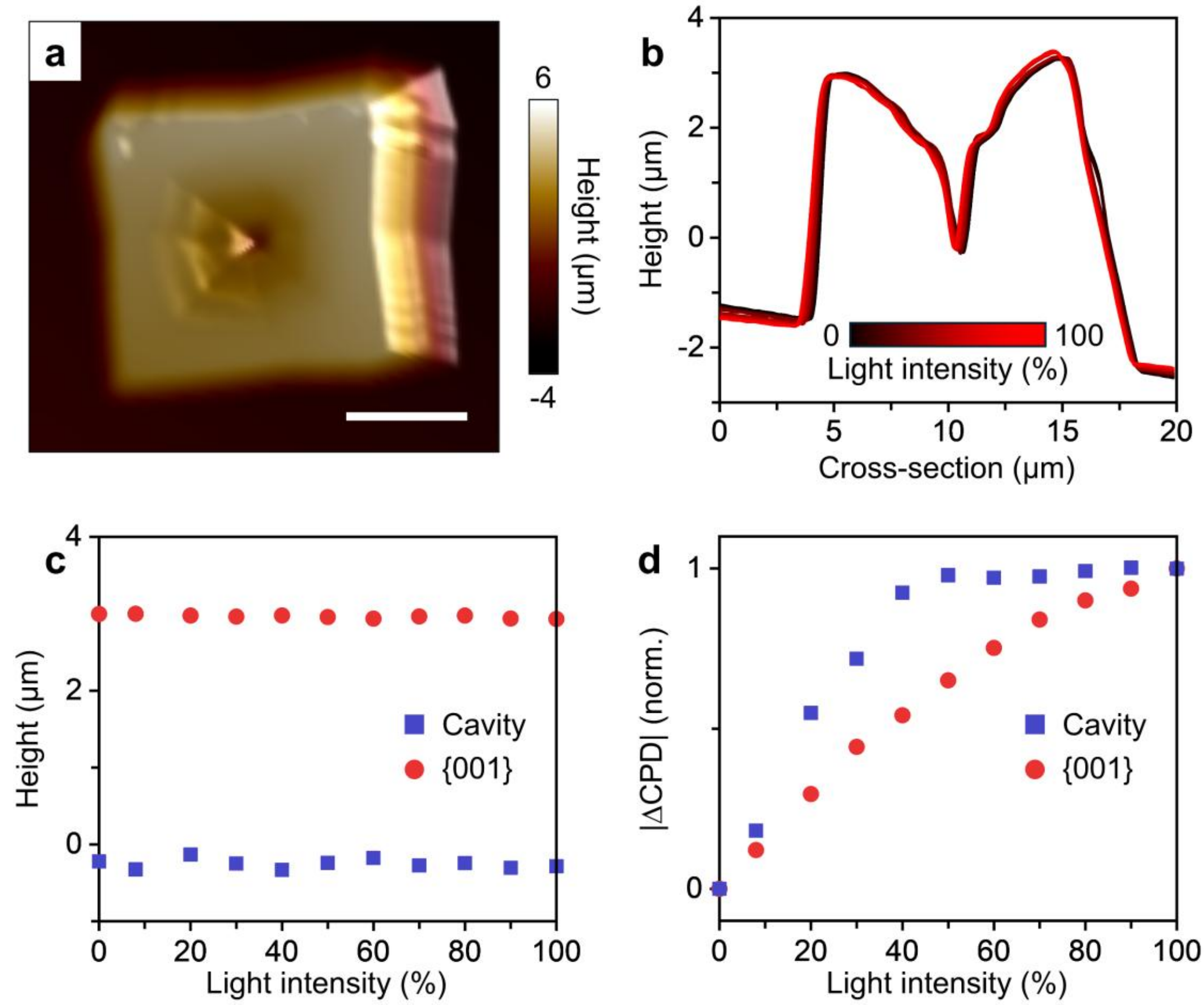

Supplementary Figure 7. Effect of excitation illumination intensity on AFM detection and photo-induced CPD variations (SPV). a, AFM image of a cavity-structured Cu_2O particle used for this investigation. Scale bar, 5 μm. **b**, AFM height profiles across the particle in **a**, collected under different excitation illumination intensities (100% intensity corresponds to 80 mW cm^{-2}). **c**,**d**, Height (**c**) and SPV magnitude (|ΔCPD|, **d**) values measured in the cavity and {001} regions as a function of illumination intensity. The SPV magnitudes were normalized to the value at 100% light intensity to directly compare the evolution of SPV signals with light intensity at the cavity and {001} surface regions.

The morphology and height values measured by AFM show no dependence on illumination intensity, with only minor and illumination-independent fluctuations

observed. These results indicate that the influence of excitation illumination on AFM detection is negligible in our measurements. The dependence of SPV on illumination intensity reveals that the SPV signals in the cavity region saturate at ~50% of the maximum light intensity, whereas the SPV signals on the {001} surface do not saturate even at 100% intensity. This behavior suggests that the effective light intensity within the cavity is significantly higher than that on the {001} surface.

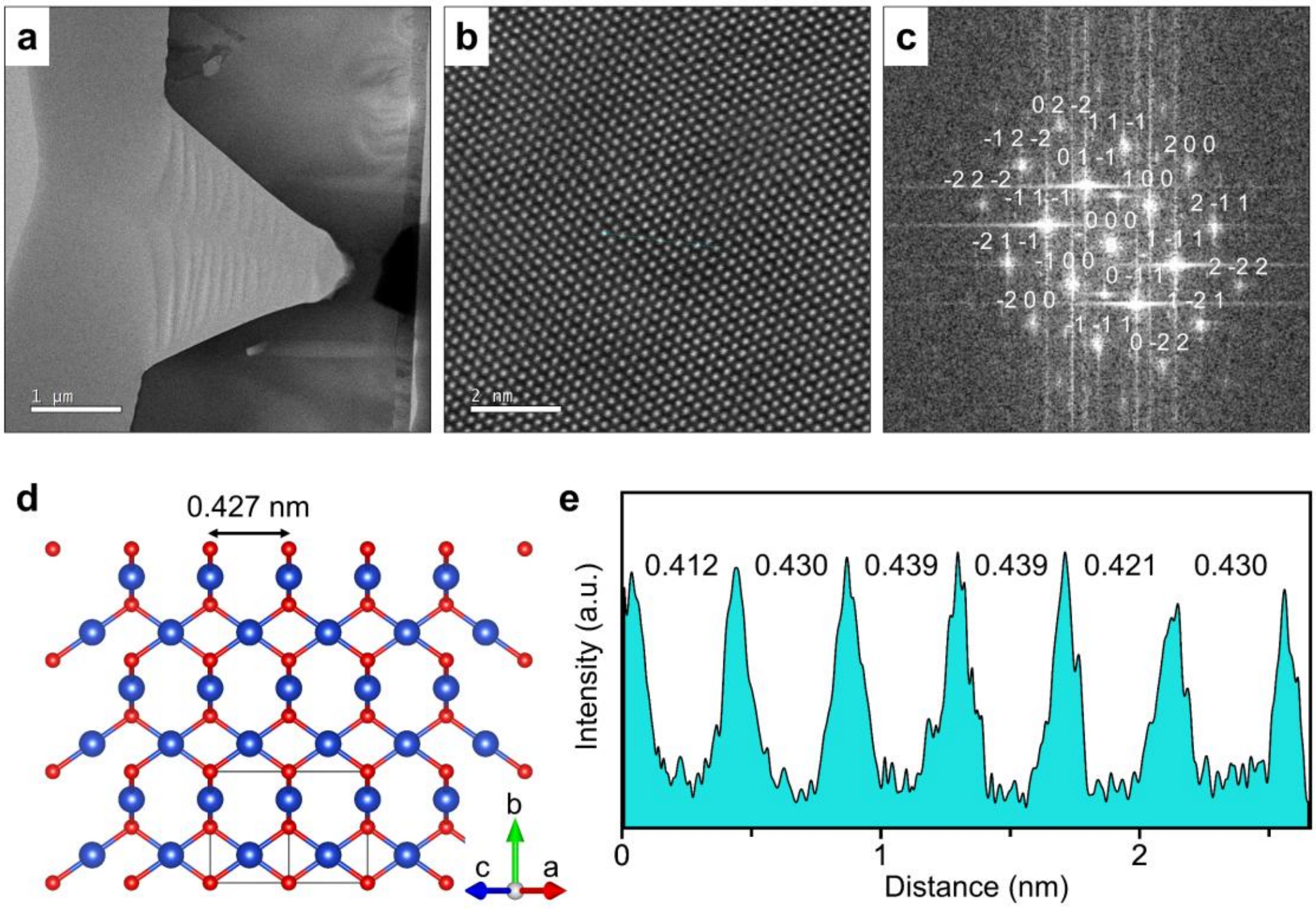

Supplementary Figure 8. Atomic structure of the Cu_2O cavity region. a, Cross-sectional electron microscopy image showing the preparation of the TEM sample by focused ion beam (FIB) milling along the diagonal of the {001} facet. **b**, Atomic-resolution annular dark-field (ADF) STEM image taken beneath the cavity surface. Bright spots represent Cu atomic columns. **c**, Fast Fourier transform (FFT) of the ADF-STEM image in **b**, showing a pattern consistent with the [011] orientation. **d**, Schematic illustration of the atomic structural model of Cu_2O viewed along the {011} facet. Blue and red spheres represent Cu and O atoms, respectively. The theoretical Cu–Cu distance is 0.427 nm. **e**, Line profile of ADF intensity extracted along the line in **b**, showing Cu–Cu distances consistent with the [011] orientation.

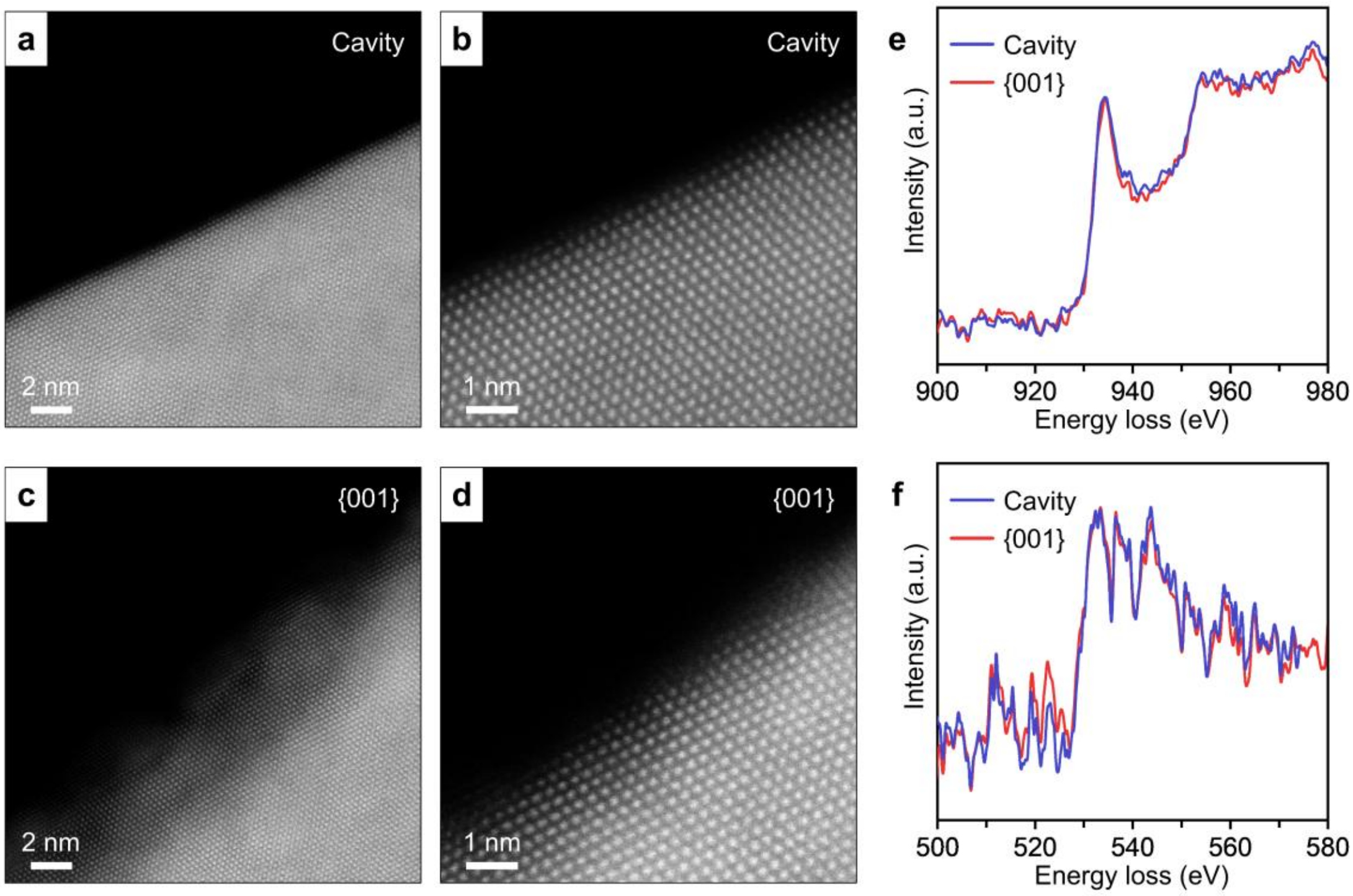

Supplementary Figure 9. Structural comparison between cavity and {001} surface regions at the atomic scale. a–d, Low- (**a**,**c**) and high-magnification (**b**,**d**) ADF-STEM images acquired from the cavity (**a**,**b**) and {001} surface regions (**c**,**d**). **e**,**f**, EELS spectra of the Cu L-edge (**e**) and O K-edge (**f**) collected from the cavity and {001} surface regions.

ADF-STEM images show that vacancy defects are predominantly distributed within the outermost atomic layers of both the cavity and {001} surface regions, whereas the subsurface regions exhibit well-ordered atomic arrangements with a low density of vacancy defects. The Cu L-edge and O K-edge EELS spectra exhibit nearly identical intensities and peak positions in both regions, indicating comparable defect densities, as variations in defect concentration would result in discernible changes in spectral intensity.

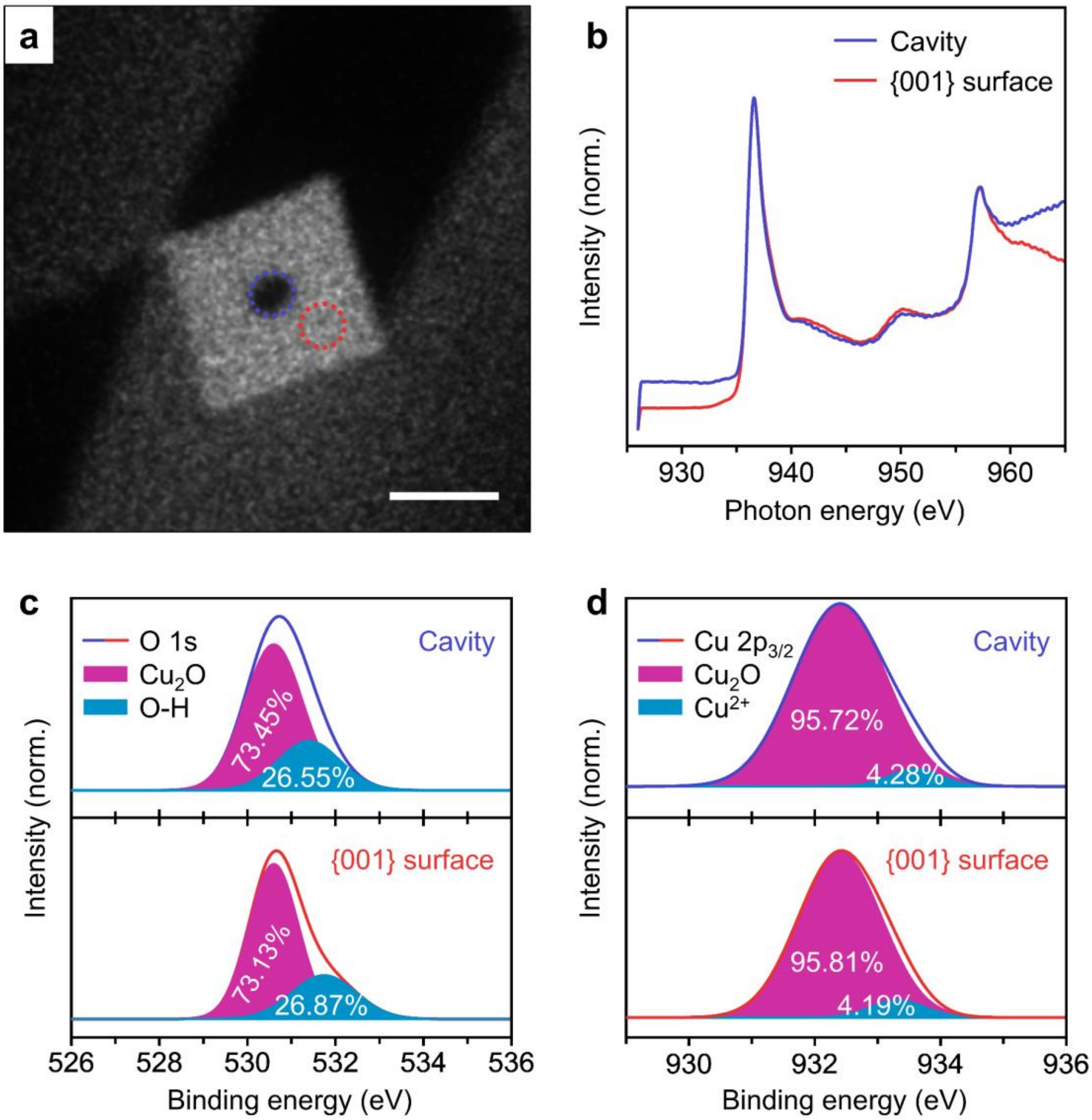

Supplementary Figure 10. Spatially resolved X-ray spectroscopy of cavity-structured Cu_2O. a, XPEEM image of a cavity-structured Cu_2O particle. Scale bar, 5 µm. The photoemission image was acquired using synchrotron-based X-ray illumination with photoelectrons collected in an integrated mode. Blue and red circles indicate the cavity and {001} surface regions, respectively, from which local spectra were extracted. **b**, Cu $L_{2,3}$-edge X-ray absorption spectra recorded from the cavity and {001} surface regions indicated in **a**. The spectra were normalized to the same intensity at 958 eV. The identical white-line intensity at ~933.5 eV for the two regions indicates

the same Cu oxidation state [19]. **c**, O 1s X-ray photoelectron spectroscopy (XPS) spectra from the cavity and {001} surface regions. The O 1s spectra were fitted with two components: a main peak at 530.6 eV corresponding to lattice oxygen in Cu_2O, and a higher-binding-energy peak at 531.4 eV attributed to surface hydroxyl species [20]. The identical O 1s chemical states suggest similar oxygen-related defect characteristics in the two regions. **d**, Cu $2p_{3/2}$ XPS spectra recorded from the cavity and {001} surface regions. The spectra were fitted with two components centered at approximately 932.4 eV and 933.6 eV, corresponding to Cu^+ in Cu_2O and Cu^{2+} species, respectively [21]. The Cu^{2+} component is commonly associated with copper-vacancy–related defects formed during crystal growth, where Cu^{2+} species from solution occupy vacant Cu sites. The similar Cu^{2+} proportions observed in the cavity and {001} surface regions indicate comparable defect densities in the two regions. Together, the spatially resolved X-ray spectroscopic results provide direct evidence that the defect distribution is uniform across the cavity and {001} surface regions, thereby excluding heterogeneous defect trapping as the origin of the observed charge separation.

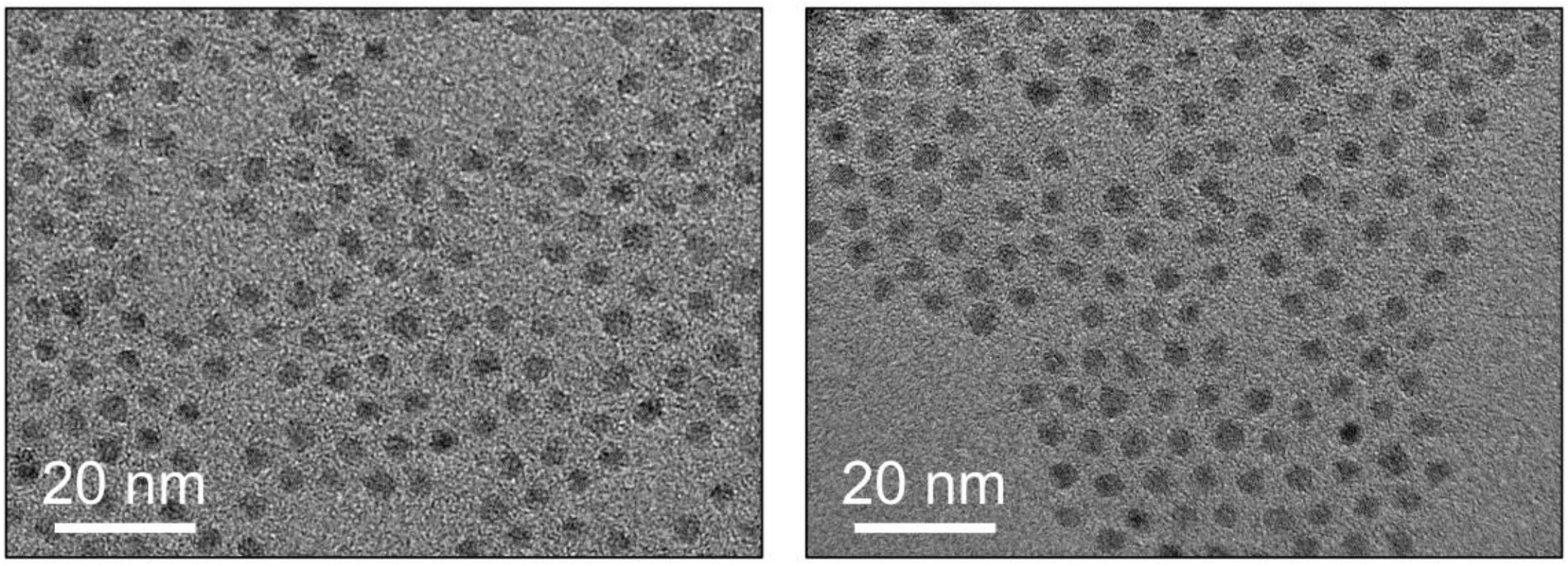

Supplementary Figure 11. TEM images of CdS quantum dots used as fluorescent probes. The images show uniform morphology and size distribution (3–4 nm) of the as-synthesized CdS quantum dots.

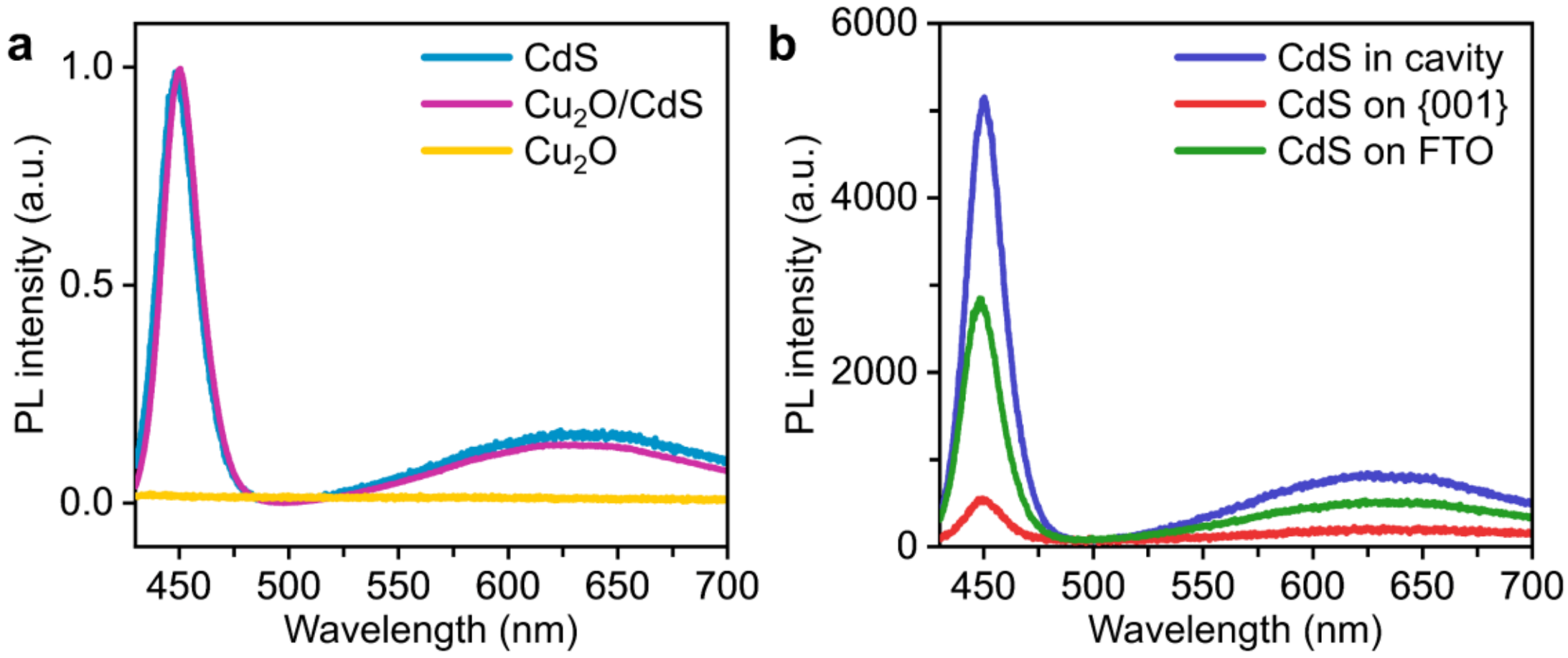

Supplementary Figure 12. PL spectra. a, Ensemble-averaged PL spectra of CdS QDs, Cu_2O, and Cu_2O loaded with CdS QDs (denoted as Cu_2O/CdS). The PL spectra of CdS QDs and CdS-loaded Cu_2O particles exhibit two emission bands. The narrow peak at ~450 nm is assigned to band-edge emission, while the broader band spanning 550–750 nm is attributed to surface trap–related emission [22]. In contrast, bare Cu_2O particles show no detectable PL signal within the measured spectral range, enabling CdS QDs to serve as effective fluorescent labels. **b**, Spatially resolved PL spectra collected from the Cu_2O cavity, {001} surface, and fluorine-doped tin oxide (FTO) substrate regions of CdS QDs-decorated Cu_2O samples. The PL peak intensity in the cavity region is approximately 9.5 times higher than that of the {001} surface, consistent with the 8–10-fold enhancement observed in PL imaging.

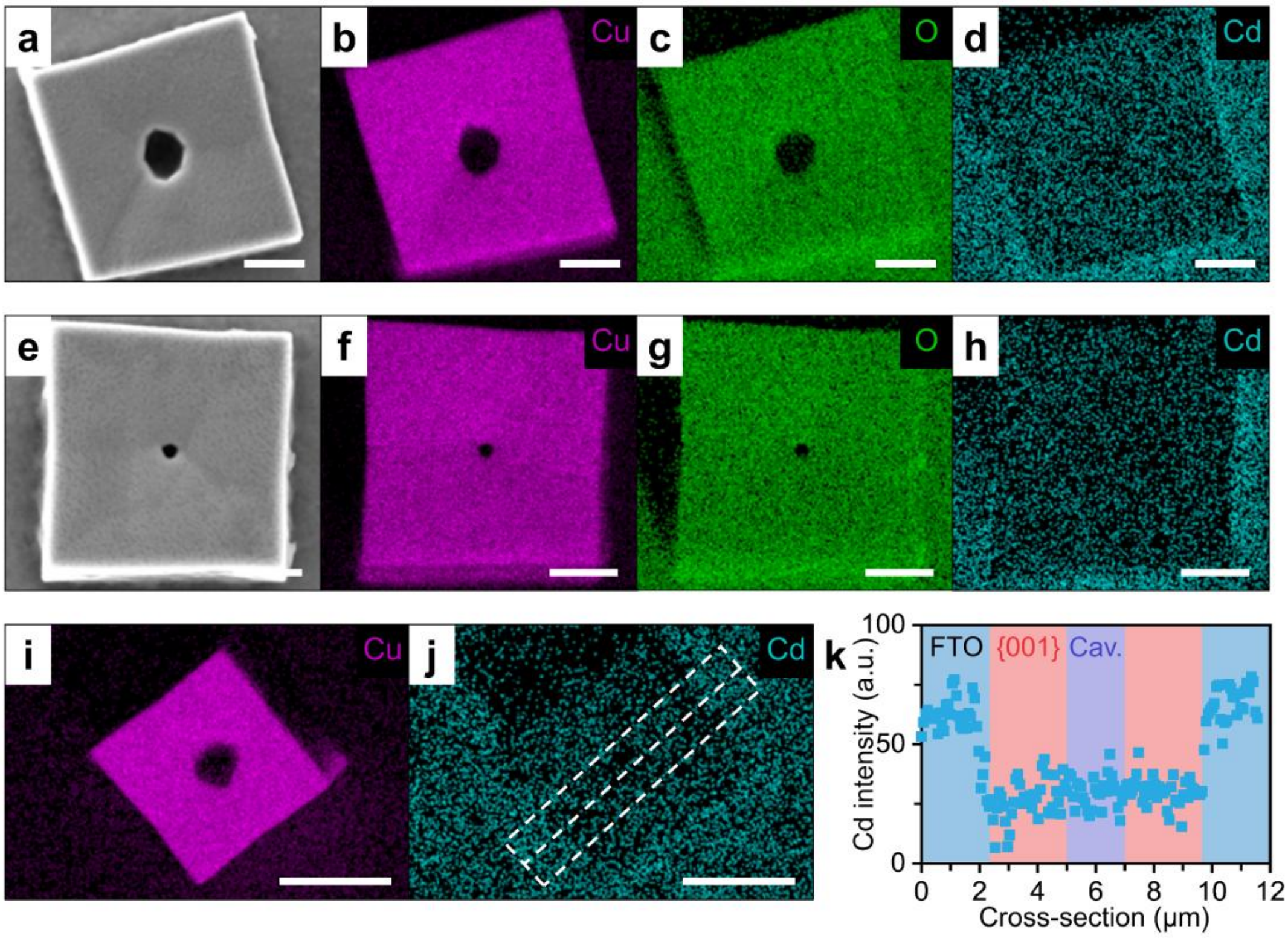

Supplementary Figure 13. SEM-EDS characterization of cavity-structured Cu_2O particles loaded with CdS QDs. a–h, SEM images (**a**,**e**) and the corresponding EDS elemental maps showing the spatial distributions of Cu (**b**,**f**), O (**c**,**g**), and Cd (**d**,**h**). Scale bars in **a–h**, 2 μm. **i–k**, EDS elemental maps of Cu (**i**) and Cd (**j**), and the corresponding Cd intensity distribution profiles across the fluorine-doped tin oxide (FTO) substrate, Cu_2O {001} facet, and cavity regions (**k**). Scale bars in **i** and **j**, 5 μm. The EDS maps and Cd intensity profiles reveal a uniform Cd distribution across the cavity and {001} surface regions, indicating homogeneous loading of CdS QDs on the Cu_2O particles. Such uniform loading ensures the reliability of the labeling strategy, in which the PL intensity of CdS QDs reflects the local light-field intensity. Moreover, QDs preferentially adhere to the FTO substrate over Cu_2O, resulting in bright PL signals on FTO observed in Fig. 2a.

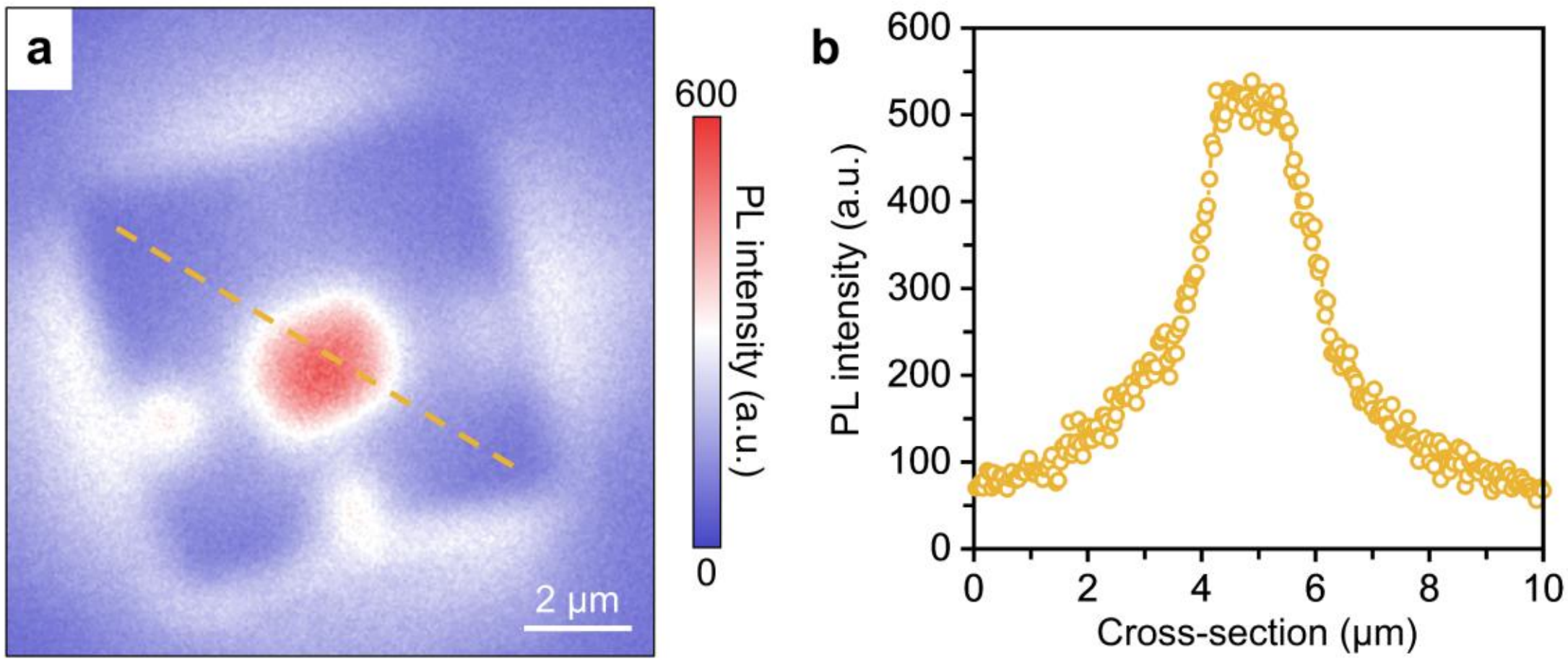

Supplementary Figure 14. PL imaging collected in the 550–600 nm wavelength range. **a**, PL image of a cavity-structured Cu_2O particle obtained by collecting emission in the 550–600 nm range, showing a significant light-intensity enhancement within the cavity region. **b**, PL intensity profile along the dashed yellow line in **a**, showing an approximately 7.5-fold enhancement within the cavity region. This enhancement is slightly lower than that obtained using the 450 nm emission (8–10 fold), likely due to the weaker emission intensity of CdS in the 550–600 nm range, which increases background contributions and thereby reduces spatial contrast under comparable signal levels.

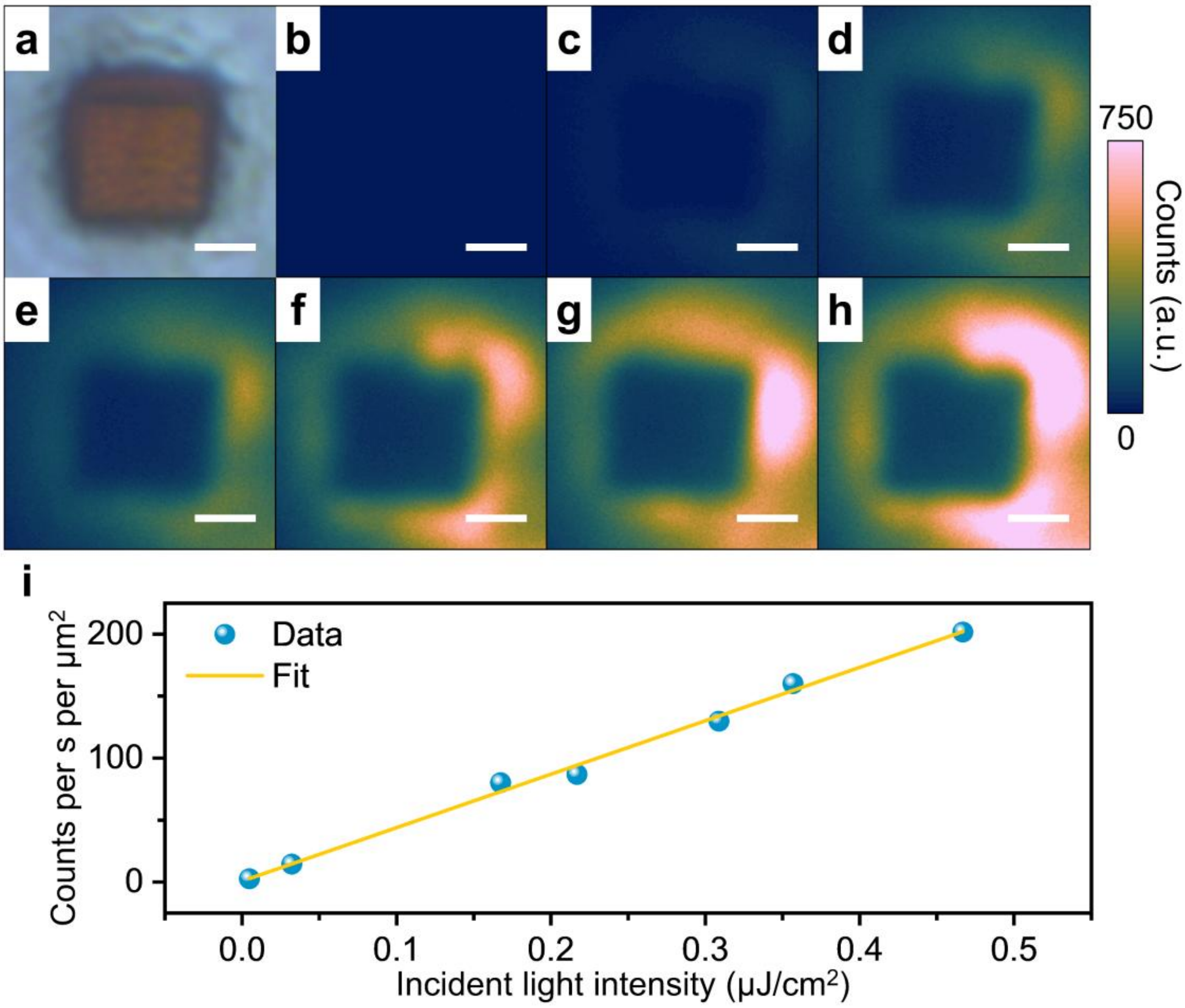

Supplementary Figure 15. Relationship between PL intensity and light-field intensity. a–h, Optical image (**a**) and the corresponding PL images (**b**–**h**) of a cubic Cu_2O particle recorded under progressively increasing laser powers. Scale bars in **a**–**h**, 2 μm. **i**, Normalized PL counts plotted as a function of incident light intensity. The data extracted from **b**–**h** were fitted using a power-law function. The fitted exponent is approximately 1, indicating a linear proportionality between the PL intensity and the light-field intensity. The resulting fitting curve is used as a calibration curve to convert PL images into quantitative light-field maps.

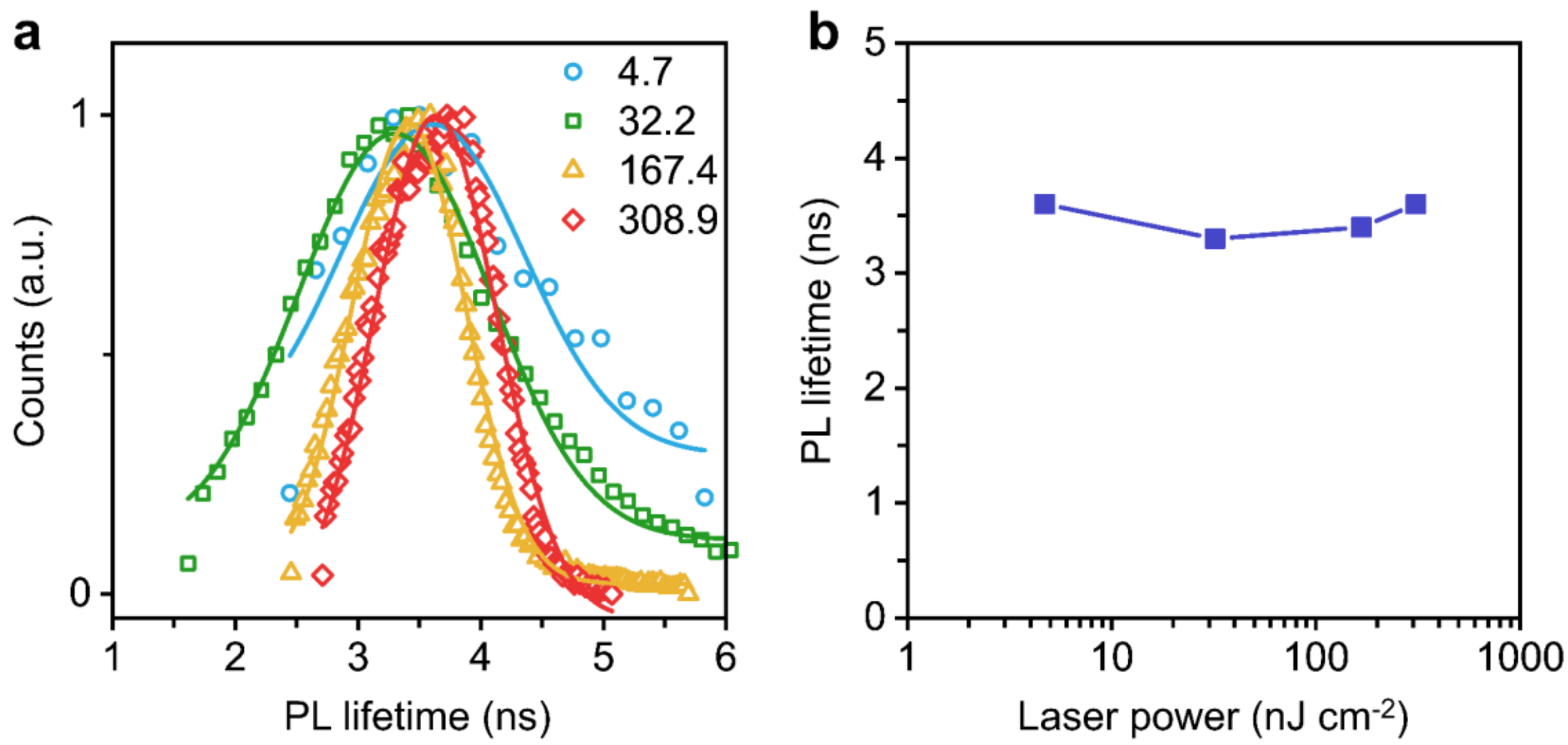

Supplementary Figure 16. Dependence of PL lifetime on excitation intensity measured on the {001} surface of a cubic Cu_2O particle. a, Statistical distributions of PL lifetimes extracted from pixel-by-pixel time-resolved PL lifetime maps recorded at different excitation intensities (cyan, 4.7 nJ cm^{-2}; green, 32.2 nJ cm^{-2}; yellow, 167.4 nJ cm^{-2}; red, 308.9 nJ cm^{-2}). The distributions were fitted with Gaussian functions (solid lines) to determine the average PL lifetime. **b**, Average PL lifetime plotted as a function of excitation intensity, with data extracted from **a**. The PL lifetime remains essentially unchanged as the excitation intensity increases by nearly one order of magnitude within the measured range, indicating that the pronounced lifetime decrease observed in the cavity region does not originate from the increased local light intensity.

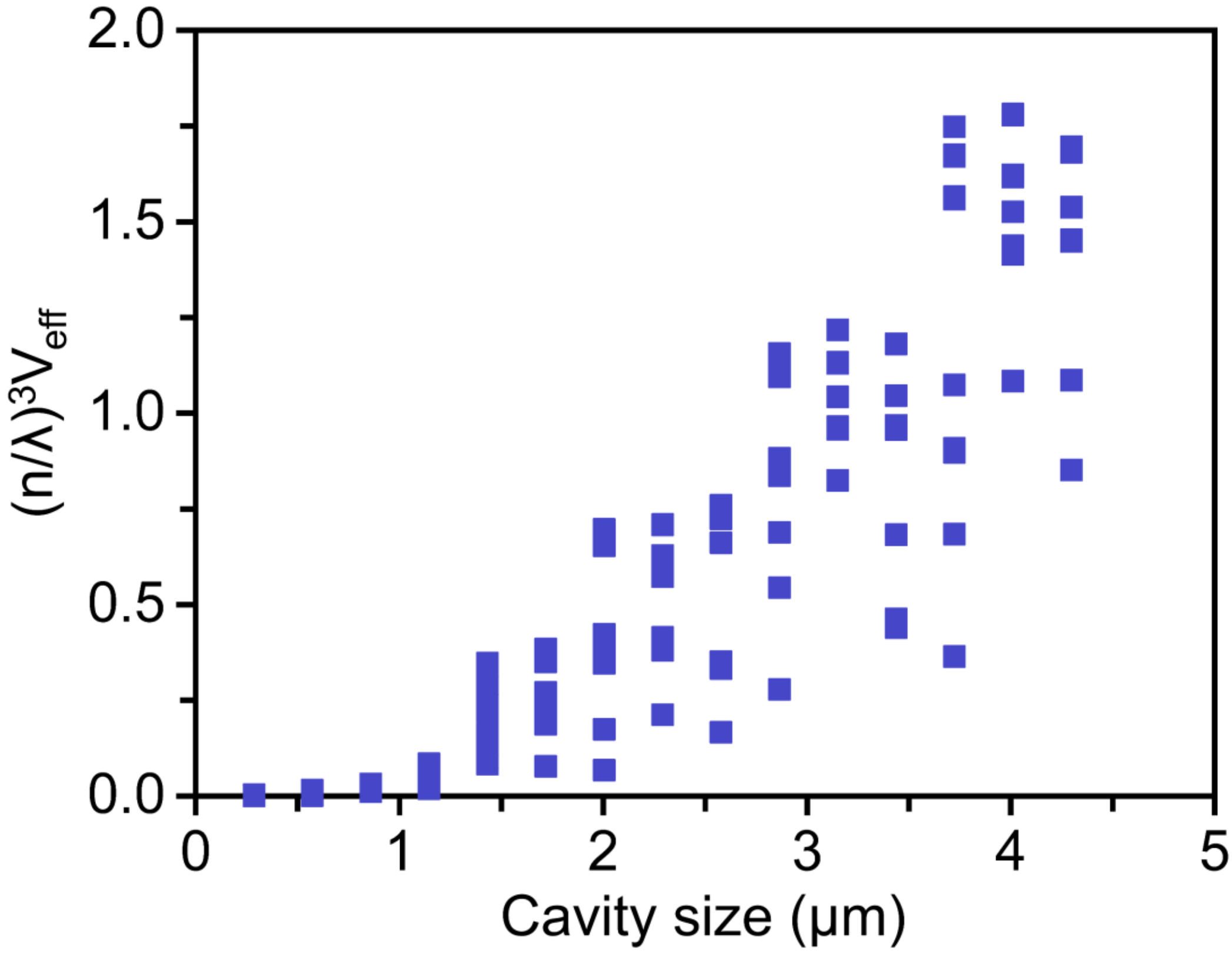

Supplementary Figure 17. Simulated effective mode volume for cavities of different sizes. The effective mode volume (V_{eff}) of cavity modes was obtained from finite-element simulations (COMSOL) for cavities with varying sizes. The calculated V_{eff} values are normalized by $(\lambda/n)^3$, where λ is the incident wavelength and n is the refractive index of the material. These results provide the effective mode volumes required for estimating the cavity quality factor based on the experimentally measured Purcell factor (see details in Supplementary Note 1).

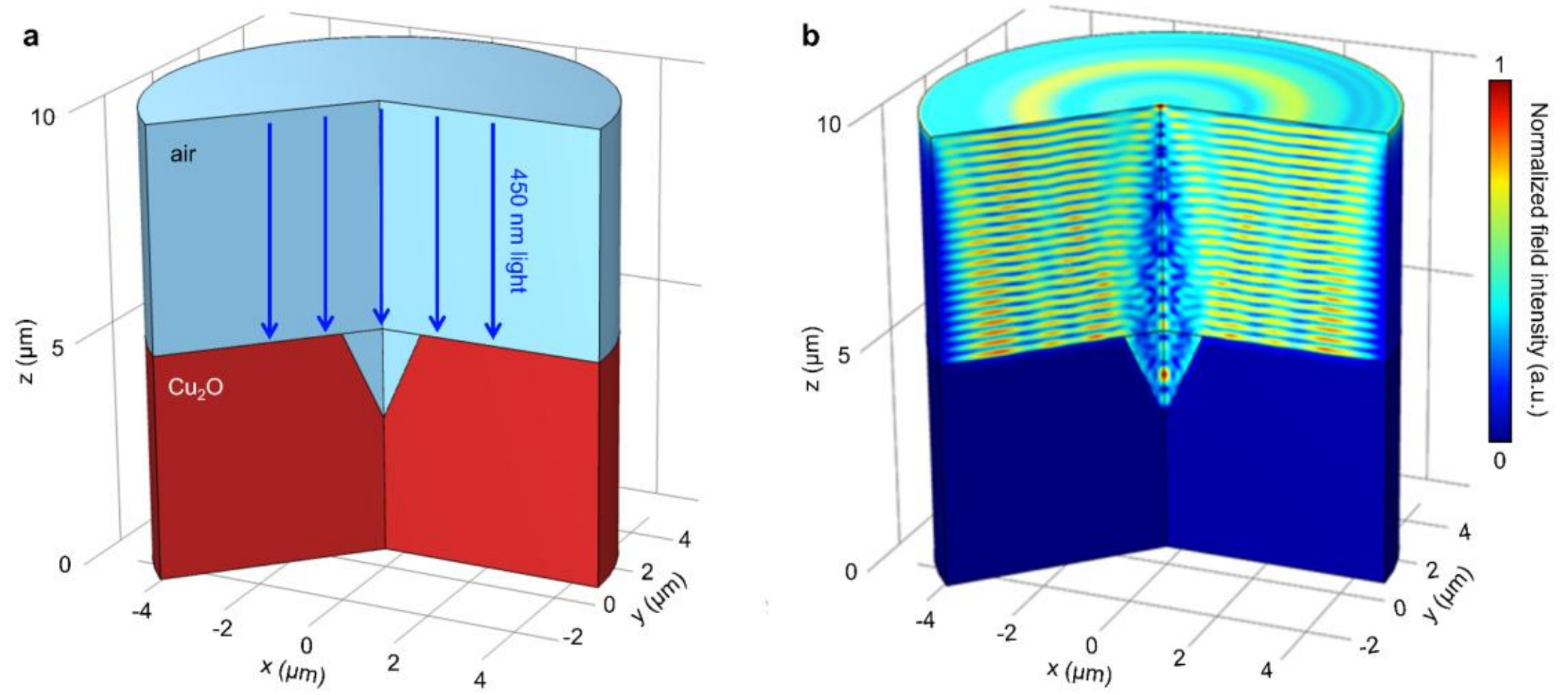

Supplementary Figure 18. Three-dimensional model used for optical simulations. **a**, Cross-sectional view of the established three-dimensional simulation model. The model consists of an air region above, a Cu_2O region below, a circular cavity structure, and a normally incident 450-nm plane wave illumination. **b**, Three-dimensional visualization of the electromagnetic field distributions simulated using the model shown in **a**.

The simulated field enhancement in **b**, predominantly confined within the air region, reflects the fundamental mechanism of optical whispering-gallery-mode resonances in enhancing light absorption. For a flat Cu_2O {001} surface, incident photons interact with the semiconductor only once, leading to a limited absorption probability, with a substantial fraction lost via single-pass reflection and vertical (out-of-plane) scattering into the air. In contrast, within a cavity structure, optical resonance leads to repeated reflection of photons along the cavity boundary, giving rise to the field enhancement observed in the simulations (Fig. 2e). As these photons are confined within the cavity air, they undergo multiple interactions with the surrounding Cu_2O, thereby significantly increasing the overall probability of light absorption compared to

the single-pass process on a flat surface. Therefore, although the enhanced field is primarily localized in the air region, it directly reflects efficient photon confinement within the cavity. This confinement governs the cumulative light absorption in the adjacent semiconductor through repeated photon–matter interactions. This behavior is governed by the cavity quality factor (Q), which quantifies the number of photon recirculations before dissipation.

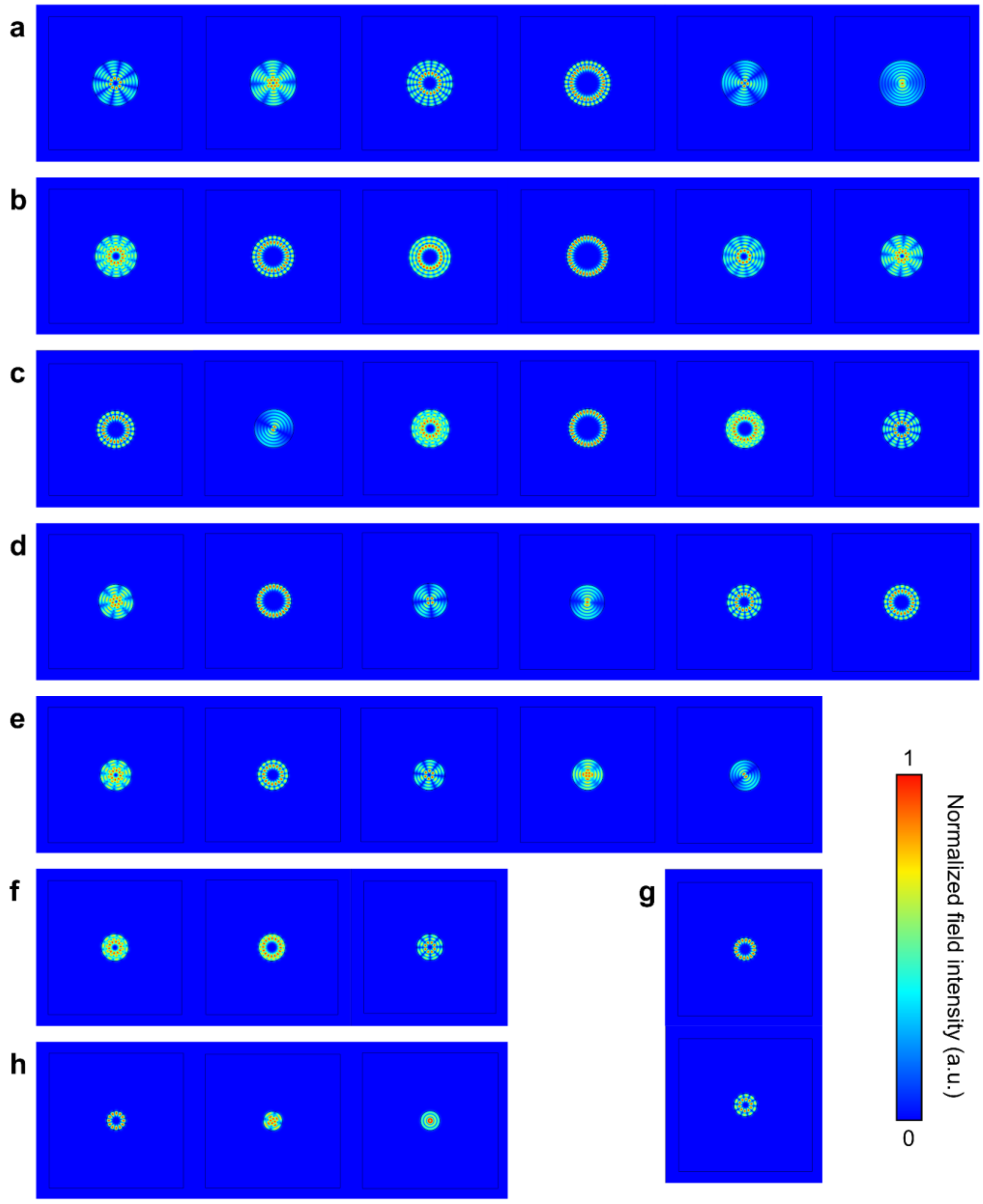

Supplementary Figure 19. Optical simulations of field-intensity enhancement at eigenfrequencies for cavities of different sizes arising from whispering-gallery resonances. a–h, Simulated electromagnetic field intensity distributions for cavities with diameters decreasing from 3 μm to 1 μm. For each cavity size, multiple eigenmodes are supported, and a quality factor (Q) was calculated for each eigenmode. For a given cavity size, the Q values of all supported eigenmodes were averaged to obtain the data shown in Fig. 2f.

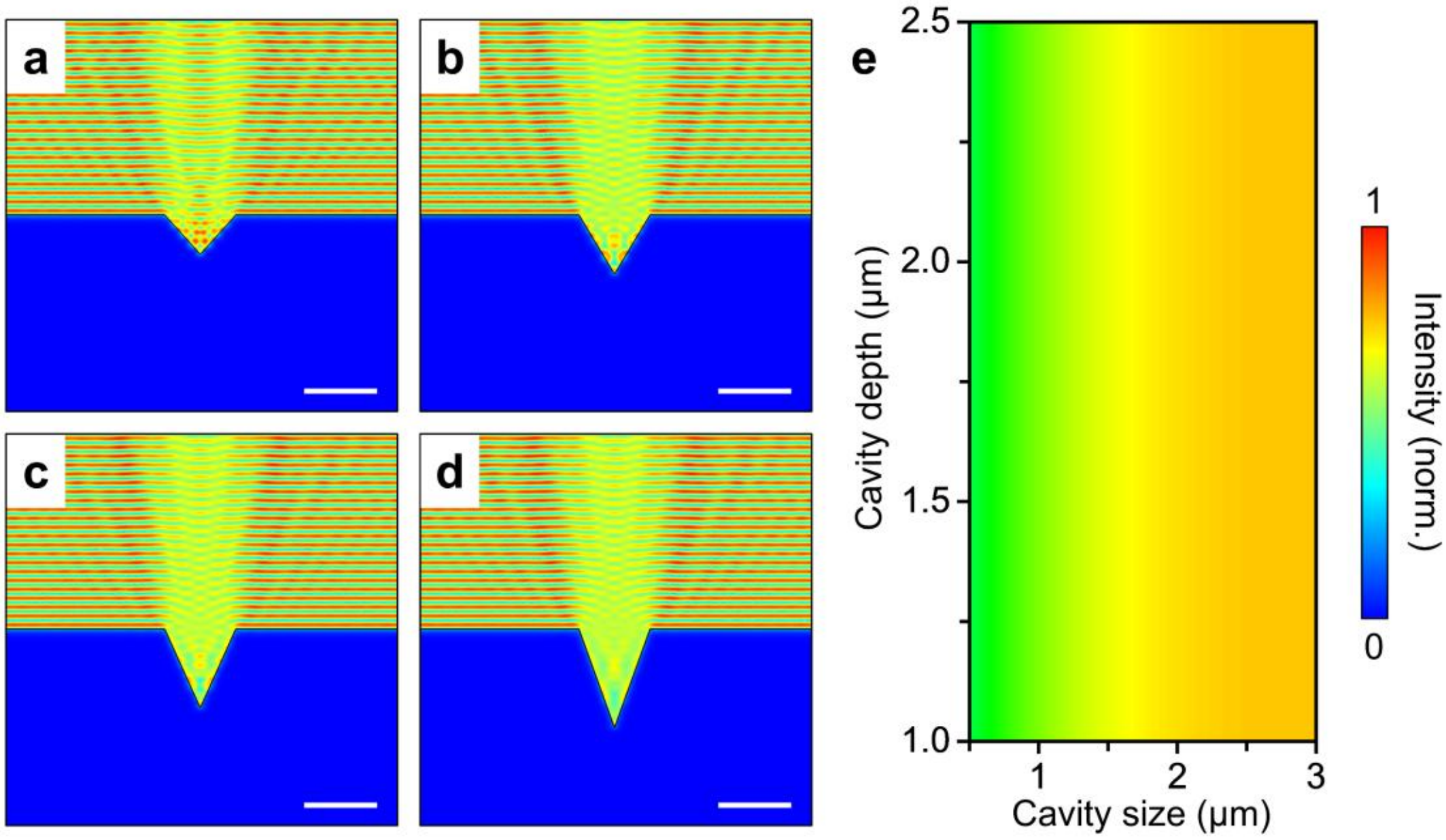

Supplementary Figure 20. Optical simulations of cavity depth–dependent light-field distributions. **a–d**, Simulated electromagnetic field distributions in the x–z plane for Cu_2O particles with a fixed cavity size of 2 μm and varying cavity depths from 1 μm to 2.5 μm (**a**–**d**). Scale bars, 2 μm. **e**, Electromagnetic field intensity distributions as a function of cavity size and depth. The simulations show that cavity-induced electromagnetic field enhancement is primarily governed by cavity size and is largely independent of cavity depth at a fixed size, consistent with its origin in laterally confined whispering-gallery modes.

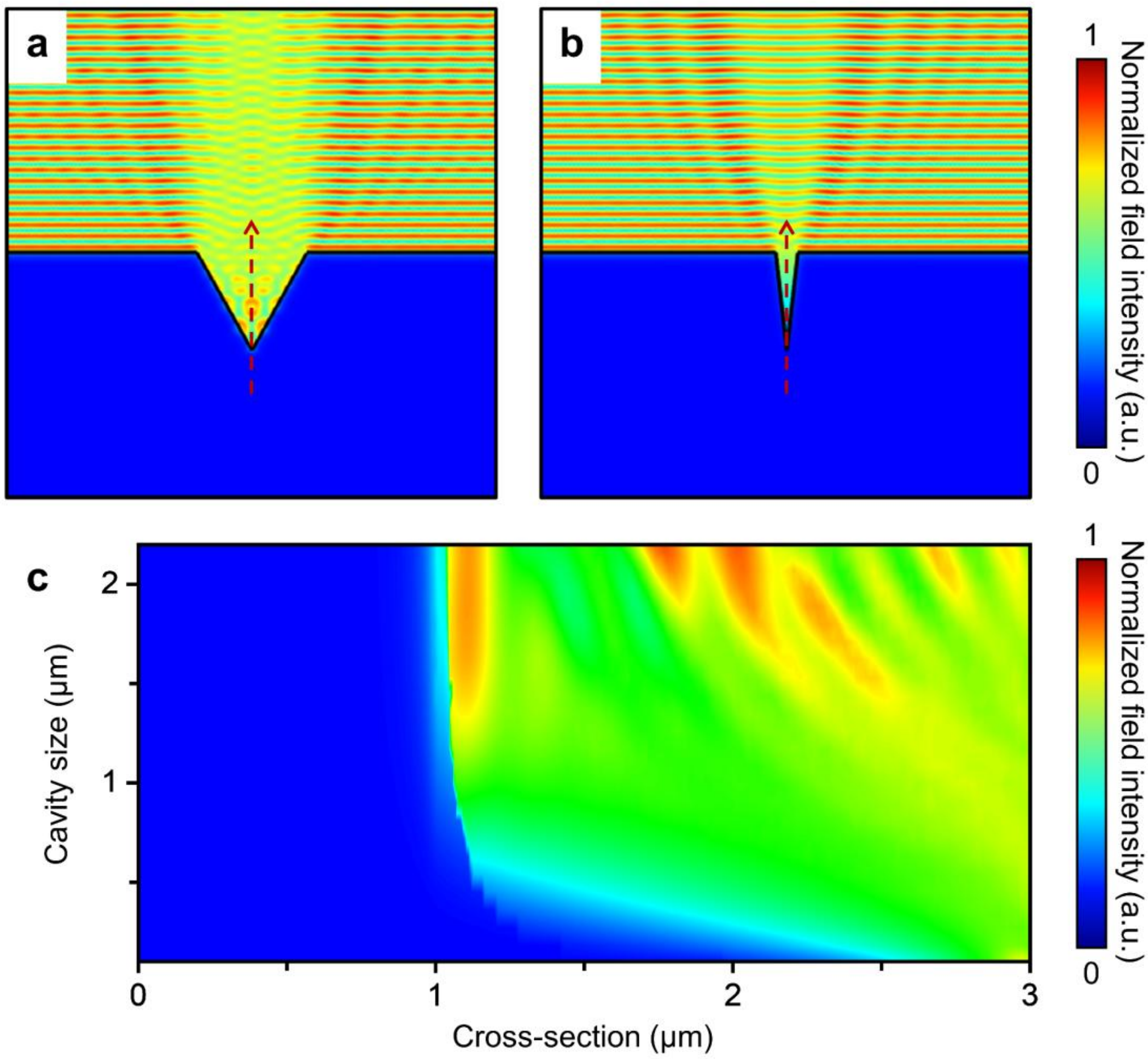

Supplementary Figure 21. Optical simulations of cavity size-dependent light-field distributions. **a**,**b**, Simulated electromagnetic field distributions in the x–z plane for Cu_2O particles with large (**a**) and small (**b**) cavity sizes. **c**, Field intensity profiles extracted along the cross-sections (dashed lines in **a** and **b**) for different cavity sizes. Cross-sections at <1 μm correspond to regions inside the Cu_2O particle, whereas cross-sections at 1–3 μm correspond to regions outside the Cu_2O particle.

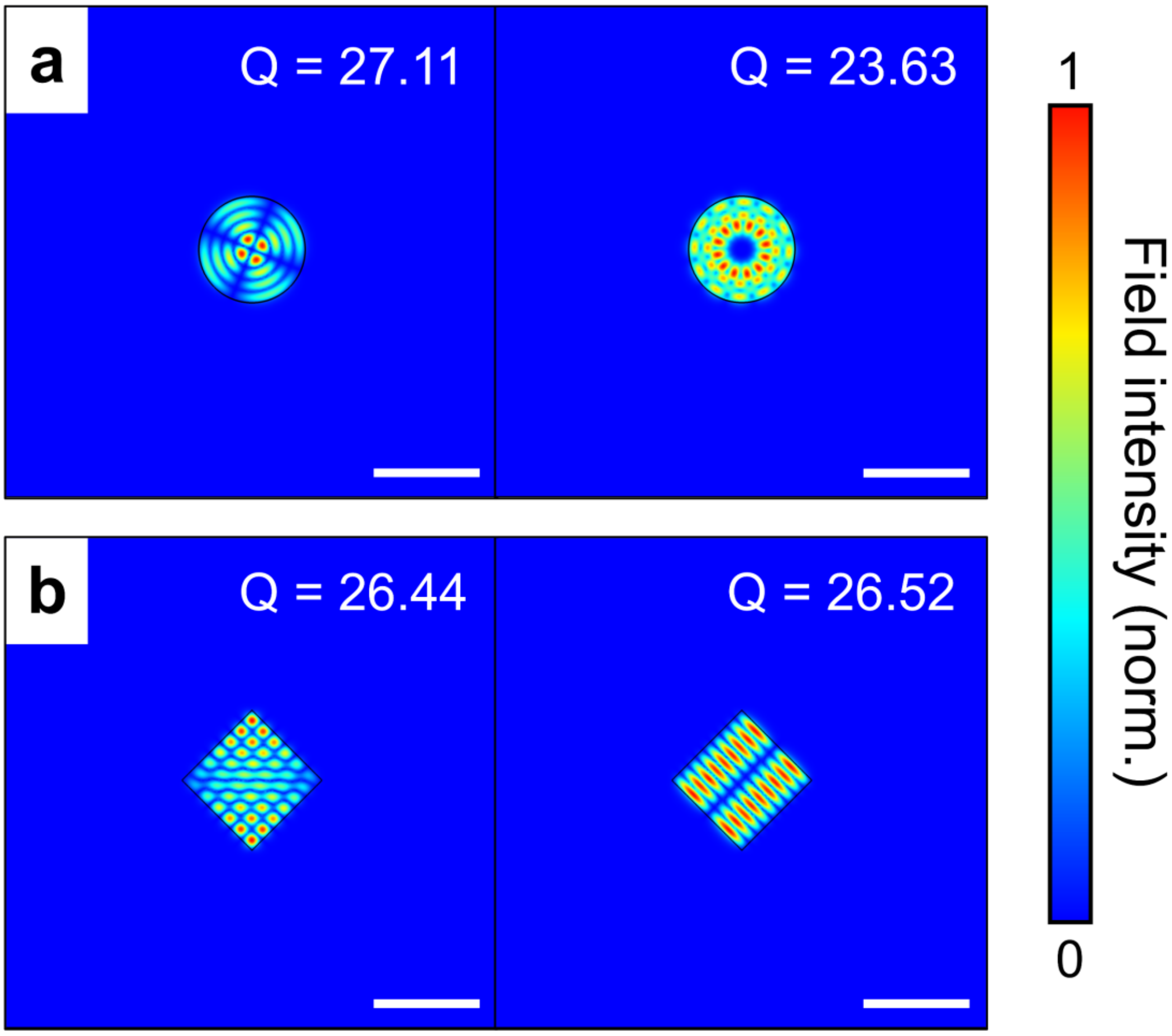

Supplementary Figure 22. Optical simulations of field-intensity enhancement for round (a) and square (b) cavities. Scale bars, 2 μm. The Q factors of each eigenmode are indicated. The simulations show that, for a cavity size of 2 μm, both round and square cavities exhibit similar Q factors of 20–30, demonstrating that the cavity shape (round vs. square) has a negligible effect on optical resonant enhancement. The Q values are also in good agreement with those estimated from experiments.

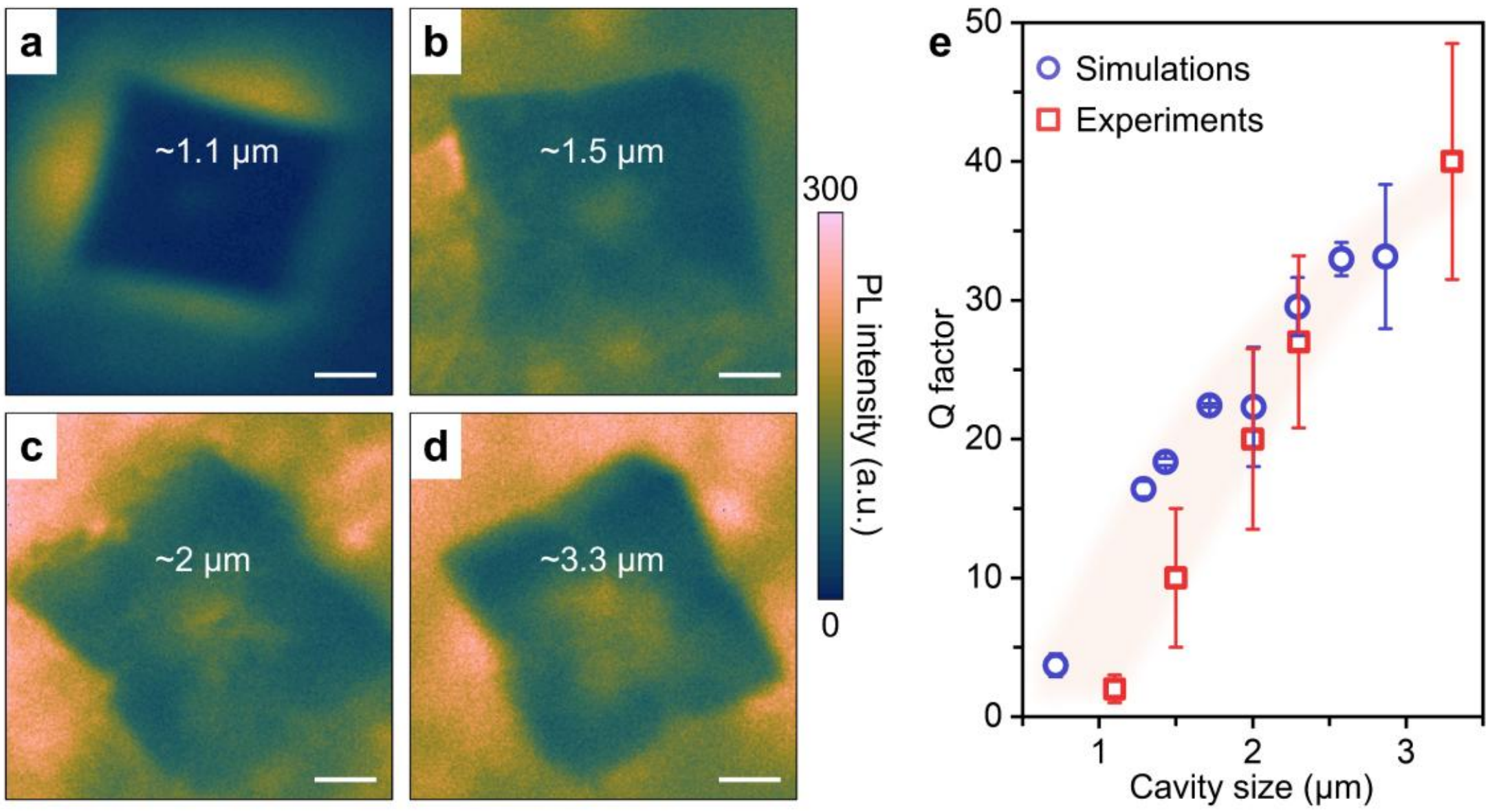

Supplementary Figure 23. Experimental determination of cavity size-dependent optical resonant enhancement. **a–d**, PL intensity images of cavity-structured Cu_2O particles with increasing cavity size from **a** to **d**. Scale bars, 2 µm. **e**, Comparison of Q factors obtained from simulations and experimental determinations for cavities of different sizes. The experimental Q values were extracted by calculating the Purcell factor from spatially resolved lifetime measurements on particles with varying cavity sizes (e.g., those shown in **a–d**), combined with simulated effective mode volumes (V_{eff}) for cavities of different sizes (see Supplementary Fig. 17). The relationship used for this extraction is described in Supplementary Note 1. Error bars reflect variations in V_{eff} across different optical resonant modes. The cavity size-dependent data show that both the optical resonance enhancement and the experimentally determined Q factors increase with cavity size, consistent with the simulations in Fig. 2f.

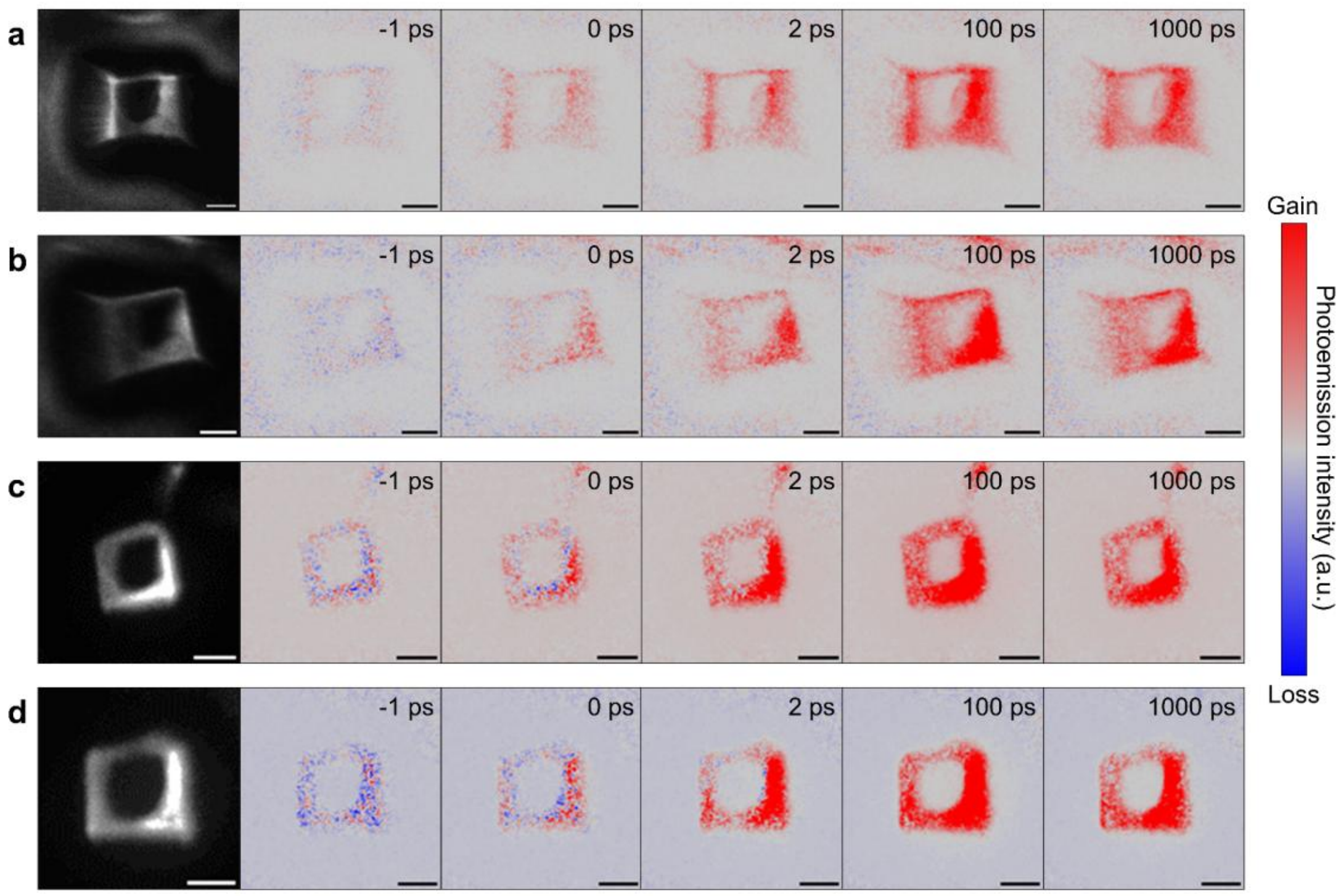

Supplementary Figure 24. PEEM and TRP-EEM images of additional cavity-structured Cu_2O particles. TR-PEEM images were recorded at different pump–probe delay times, as labeled. All scale bars, 2 µm. The results consistently show similar spatiotemporal charge dynamics, confirming the reproducibility of the ultrafast charge transfer processes.

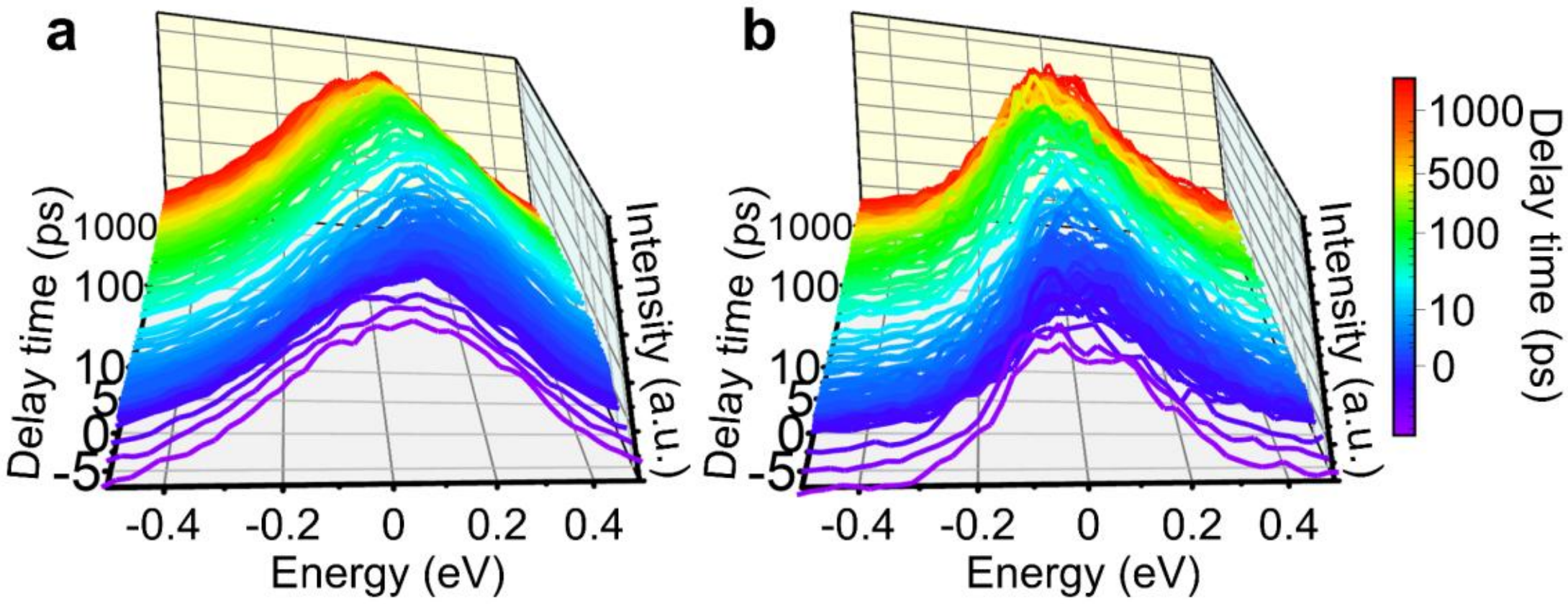

Supplementary Figure 25. Spatially resolved TRPES spectra of cavity-structured Cu_2O particles. a,**b**, TR-PES spectra collected from the {001} surface (**a**) and cavity (**b**) regions of cavity-structured Cu_2O particles. The energy at 0 eV corresponds to the conduction band minimum at negative decay times. Energy shifts observed at different pump–probe delay times were extracted as time-resolved SPV signals.

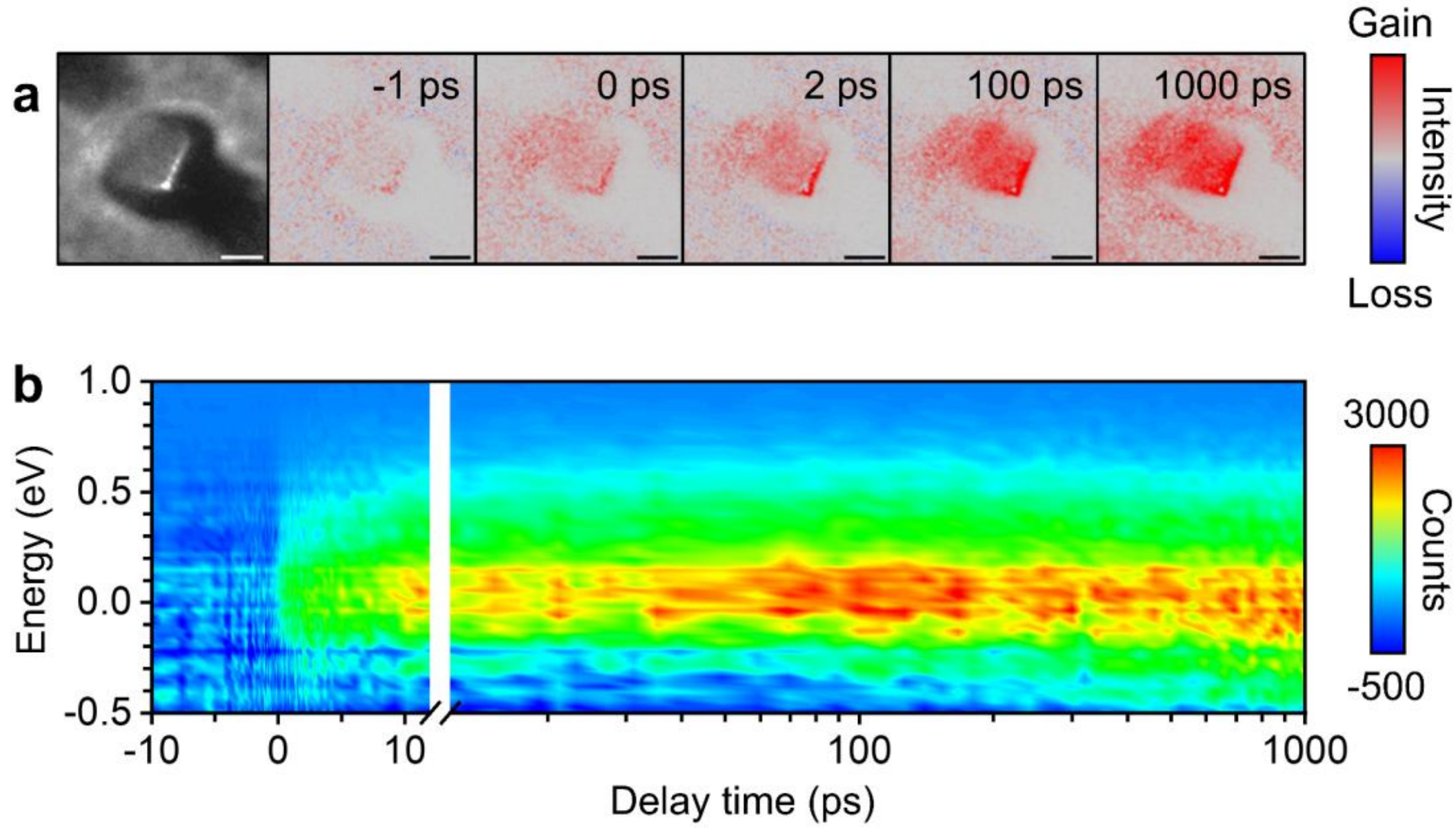

Supplementary Figure 26. TR-PEEM data of cubic Cu_2O. a, PEEM and TR-PEEM images of a cubic Cu_2O particle acquired at the pump–probe delay times indicated. Scale bars, 2 µm. **b**, TR-PES spectra collected from the {001} surface of the cubic Cu_2O particle in **a** at different delay times.

Data in **a** show that the photoelectron distribution on the surface of cubic Cu_2O is uniform, indicating the lack of lateral charge separation. Data in **b** were used to extract SPV signals that arise from bulk-to-surface charge transfer. Therefore, subtracting the SPV signals of cubic Cu_2O from those of cavity-structured Cu_2O decouples the contribution of bulk-to-surface charge transfer and isolates the SPV signals associated with lateral charge separation between the cavity and {001} surface regions.

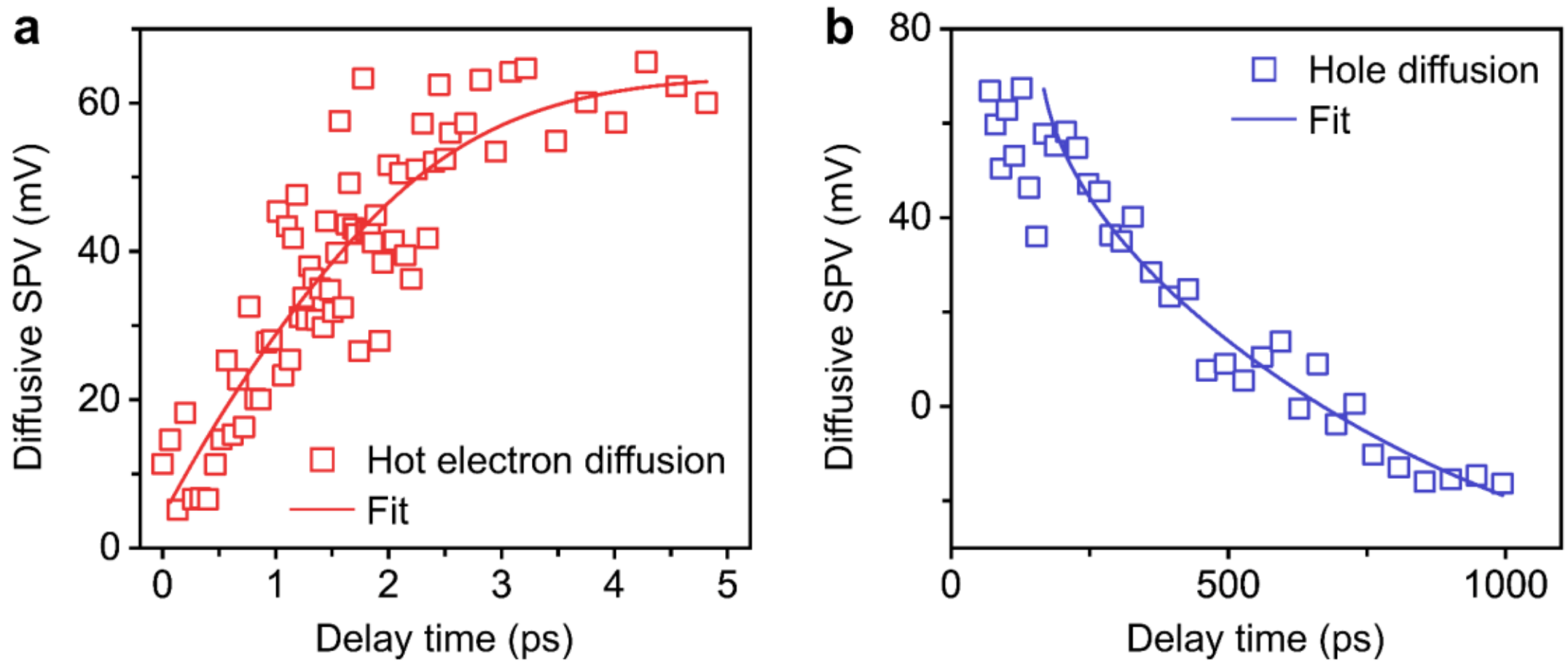

Supplementary Figure 27. Fitting of diffusive SPV. a,**b**, Fitting curves for hot electron diffusion (**a**) and hole diffusion (**b**). The experimental data used for fitting were obtained from Fig. 3d by extracting the diffusive SPV difference between the cavity and {001} surface regions. Details of the fitting procedure are provided in Supplementary Note 2.

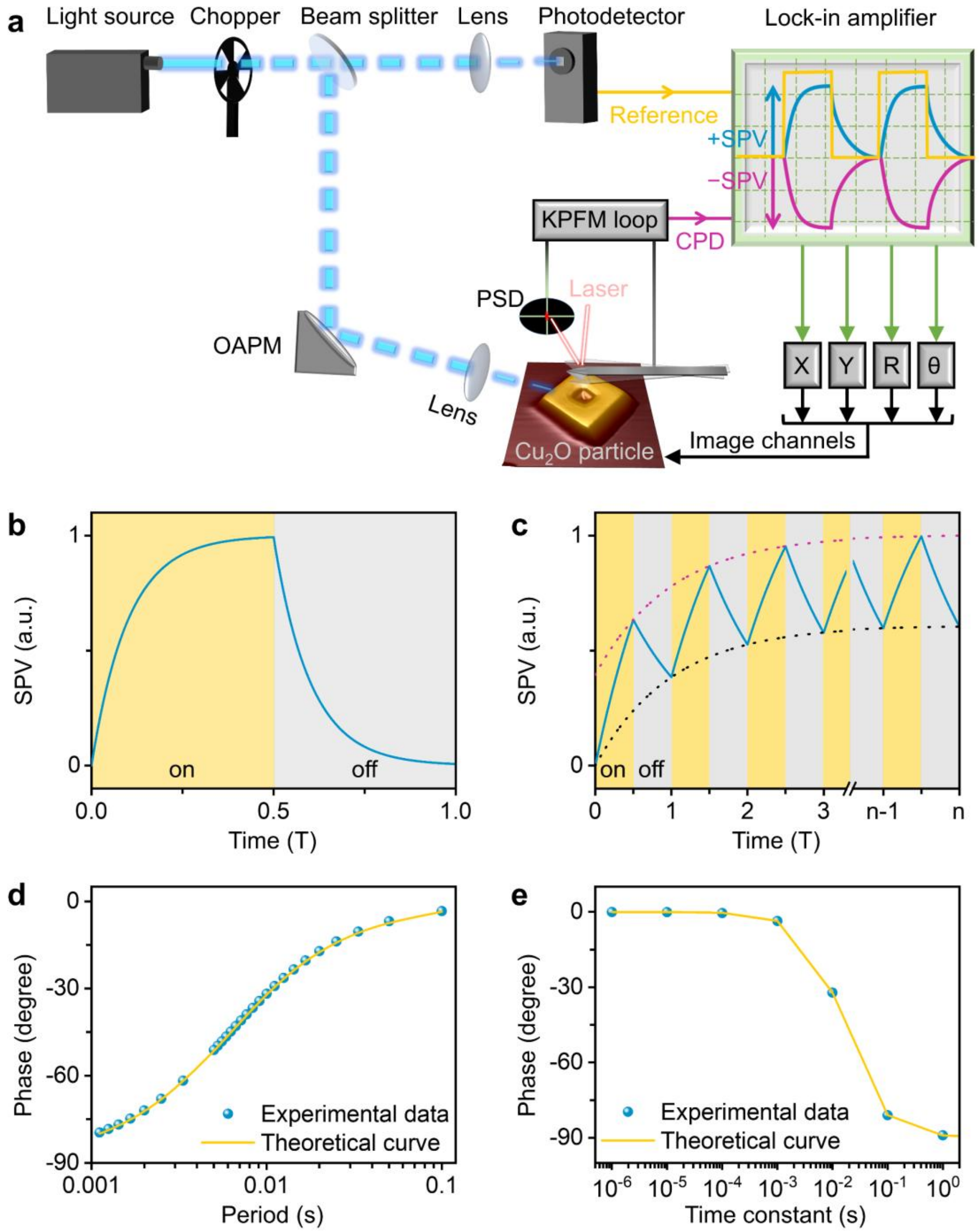

Supplementary Figure 28. Modulated SPVM. a, Experimental setup for modulated SPVM. **b**, SPV evolution within one modulation period (T) under chopped illumination for a charge-separation process with a timescale much shorter than T. **c**, SPV evolution under chopped illumination for charge-separation processes containing components with timescales longer than T. **d**, Phase signal plotted as a function of modulation period,

measured by inputting a signal with a time constant of 1 ms generated by a signal generator (cyan points). **e**, Phase signal plotted as a function of the time constant of input signals generated by a signal generator (cyan points) at a fixed modulation frequency of 10 Hz. The theoretical curves (yellow lines) in **d** and **e** were obtained from analytical derivations of the functional relationships between phase and modulation period or SPV time constant, as detailed in Supplementary Note 4. The measured data (cyan points) show excellent agreement with theory, validating the developed modulated SPVM method.

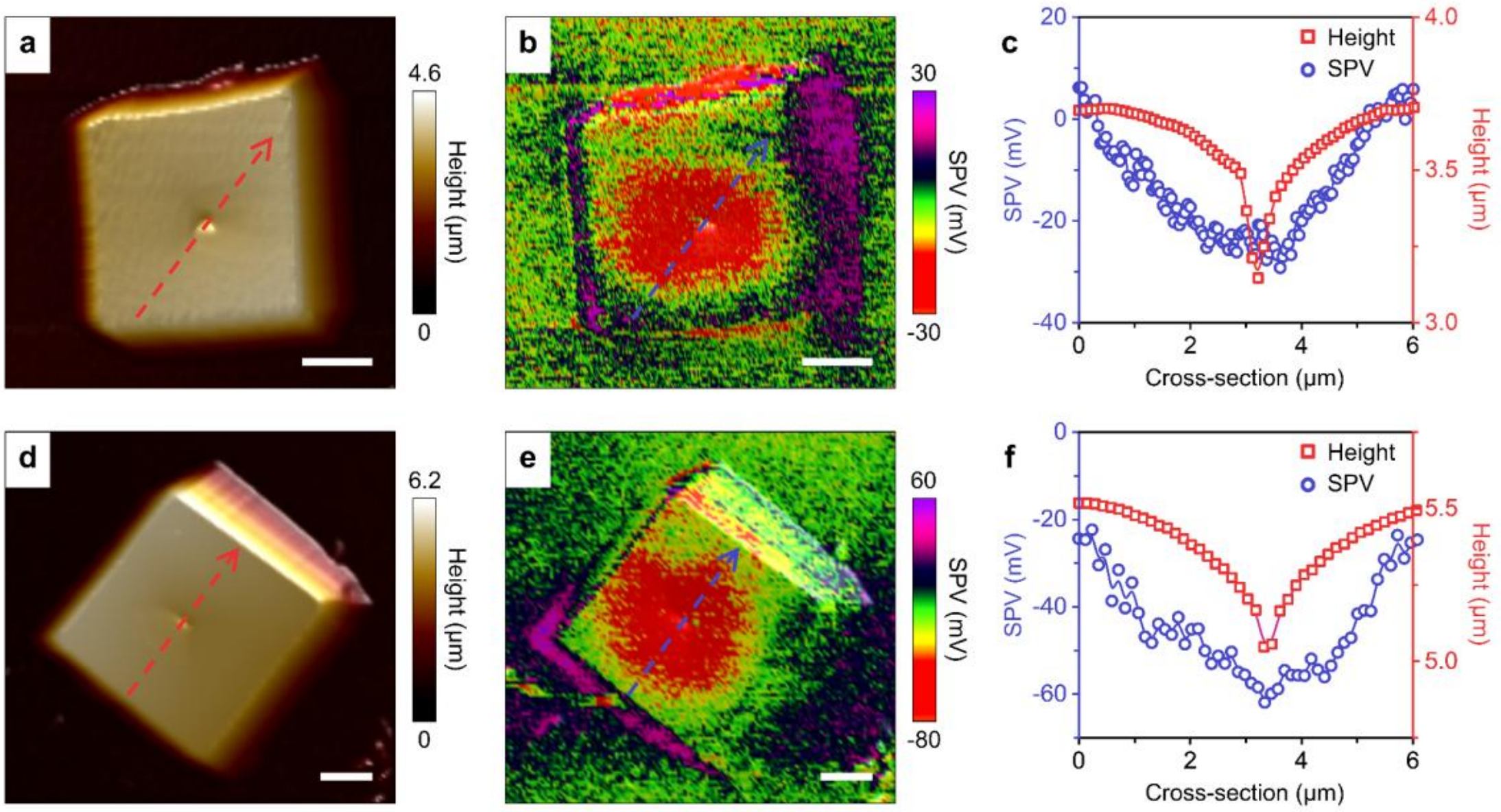

Supplementary Figure 29. Reproducibility of SPVM data on Cu_2O particles with small cavity sizes. a–**f**, AFM images (**a**,**d**), SPVM images (**b**,**e**), and the corresponding extracted (blue) and height (red) profiles across the cavity and {001} surface regions (**c**,**f**) for Cu_2O particles with small cavity sizes. All scale bars, 2 μm. Data in **c** were extracted along the dashed lines in **a** and **b**, and data in **f** were extracted along the dashed lines in **d** and **e**. These repeated SPVM measurements confirm that photogenerated electrons preferentially accumulate in the small cavity regions.

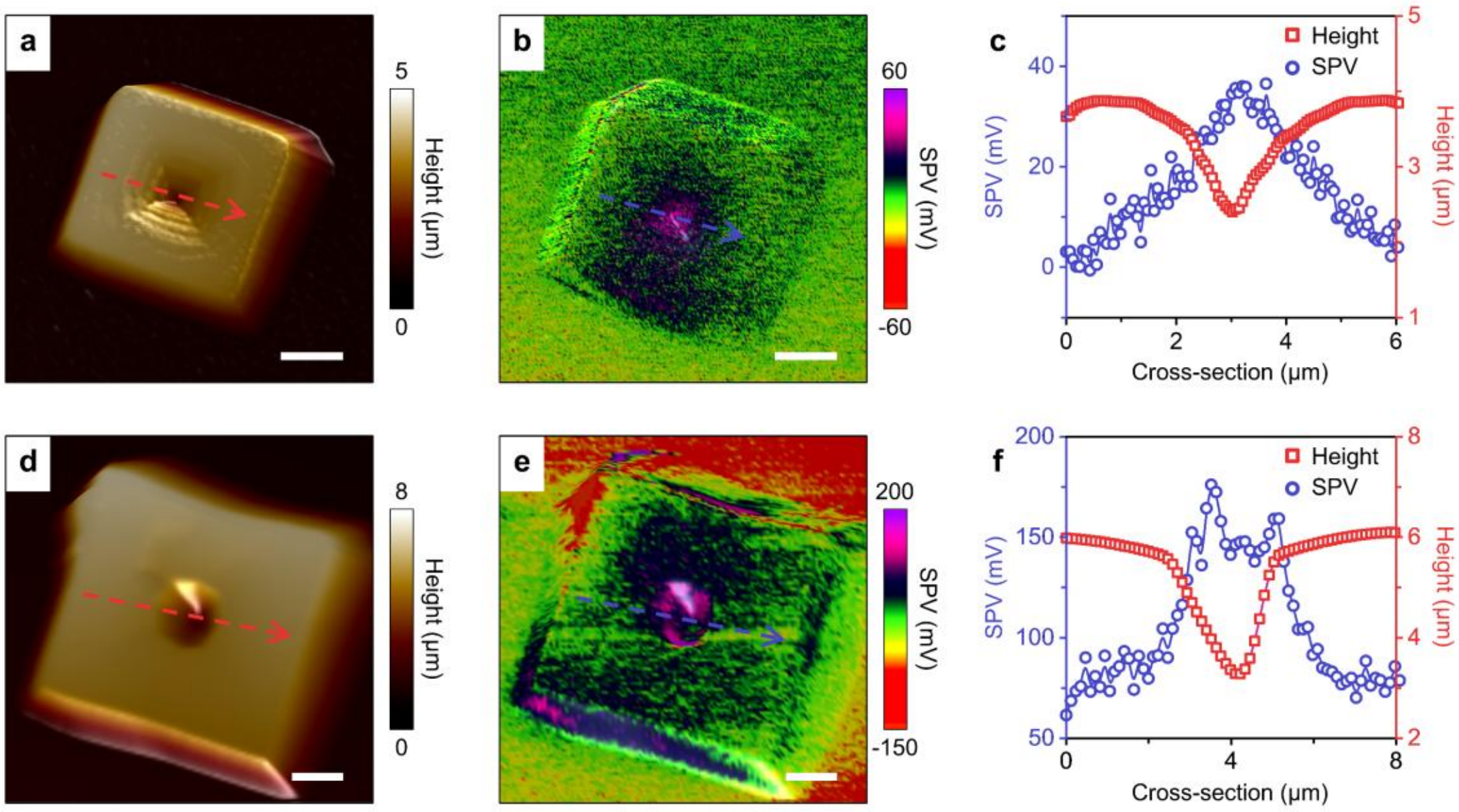

Supplementary Figure 30. Reproducibility of SPVM data on Cu_2O particles with large cavity sizes. a–**f**, AFM images (**a**,**d**), SPVM images (**b**,**e**), and the corresponding extracted (blue) and height (red) profiles across the cavity and {001} surface regions (**c**,**f**) for Cu_2O particles with large cavity sizes. All scale bars, 2 μm. Data in **c** were extracted along the dashed lines in **a** and **b**, and data in **f** were extracted along the dashed lines in **d** and **e**. These repeated SPVM measurements confirm that photogenerated holes preferentially accumulate in the large cavity regions.

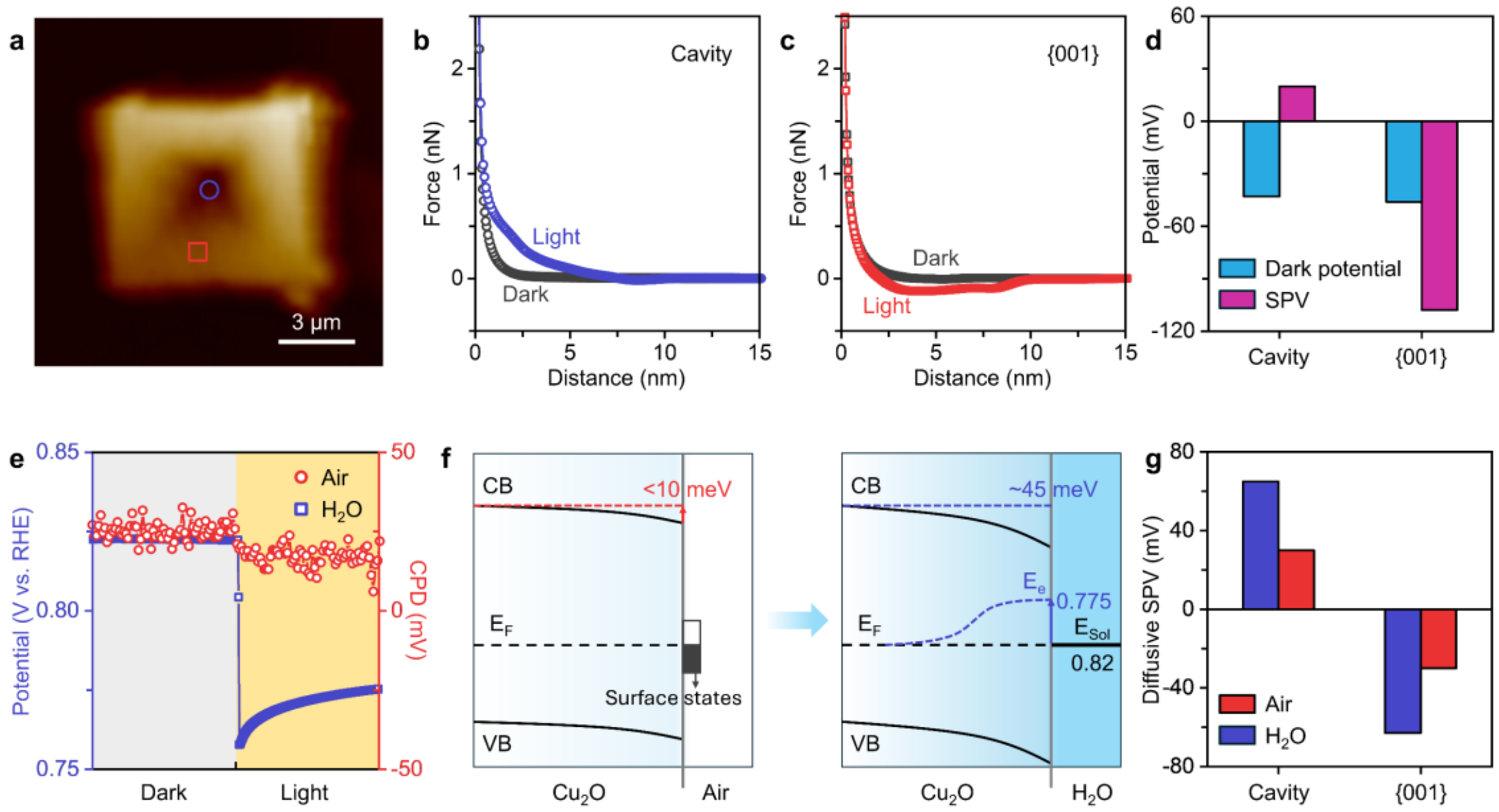

Supplementary Figure 31. Surface potential and photovoltage measurements in solution. a, In situ AFM image of a cavity-structured Cu_2O particle mapped in aqueous solution. **b**,**c**, Force–distance curves acquired from the cavity (**b**) and {001} surface (**c**) regions (as indicated in **a**) under dark and illuminated (450 nm) conditions. **d**, Surface potential and photo-induced potential differences (SPV) obtained by fitting the force–distance curves in **b** and **c** based on Derjaguin–Landau–Verwey–Overbeek (DLVO) theory [23]. **e**, Ensemble-averaged SPV measurements in air and in solution using a Kelvin probe and an in situ surface potential sensing method [24], respectively. Such laterally averaged measurements reflect charge separation arising from bulk-to-surface band bending. **f**, Surface band bending when the Cu_2O bulk Fermi level equilibrates with surface states in air or with the solution potential (E_{Sol}). CB, E_F, VB, and E_e denote the conduction band, Fermi level, valence band, and electron quasi-Fermi level, respectively. The band bending is derived from the SPV measurements in **e**. **g**, Comparison of diffusive charge separation between the cavity and {001} surface regions under air and solution conditions. The diffusive SPV in solution was obtained

by subtracting the bulk-to-surface SPV measured in **e**.

Supplementary Figs. 31a–c show that light illumination induces positive and negative electrostatic forces between the tip and sample in the cavity and {001} surface regions, respectively, indicating that photogenerated holes and electrons accumulate in the corresponding regions in aqueous solution. These results are consistent with SPVM measurements in air, indicating that the charge separation between the cavity and {001} surface regions is maintained under solution conditions. Supplementary Fig. 31d quantifies the surface potential and the photo-induced potential change based on DLVO theory. In the dark, the surface potentials at the cavity and {001} regions were both determined to be ~−46 mV, indicating that no lateral built-in electric field exists between the two regions in solution, consistent with the behavior observed in air. Under illumination, the SPV values for the cavity and {001} regions are approximately 20 mV and −110 mV, respectively, both of which are overall more negative than those measured in air. Supplementary Fig. 31e show that the negative SPV arising from downward surface band bending is significantly enhanced in solution compared to air (~−45 mV vs. ~−5 mV), rationalizing the observed overall more negative SPV in solution. The difference in band bending arises from the fact that, in air, it is determined by the equilibrium between the bulk Fermi level and surface states [4], whereas in solution it is governed by the equilibrium with the solution potential (Supplementary Fig. 31f). After subtracting the vertical bulk-to-surface SPV signals arising from surface band bending, we obtained lateral diffusive SPV signals of ~65 mV in solution, approximately twice those measured in air (Supplementary Fig. 31g). These results

indicate that the solution environment enhances surface band bending, suppresses recombination, and increases the carrier density available for lateral diffusion, thereby leading to enhanced diffusive SPV signals. The above surface potential and photovoltage measurements in solution demonstrate that the observed lateral charge separation persists and is further enhanced in aqueous environments owing to increased band bending, indicating its direct relevance for photocatalytic reactions in solution.

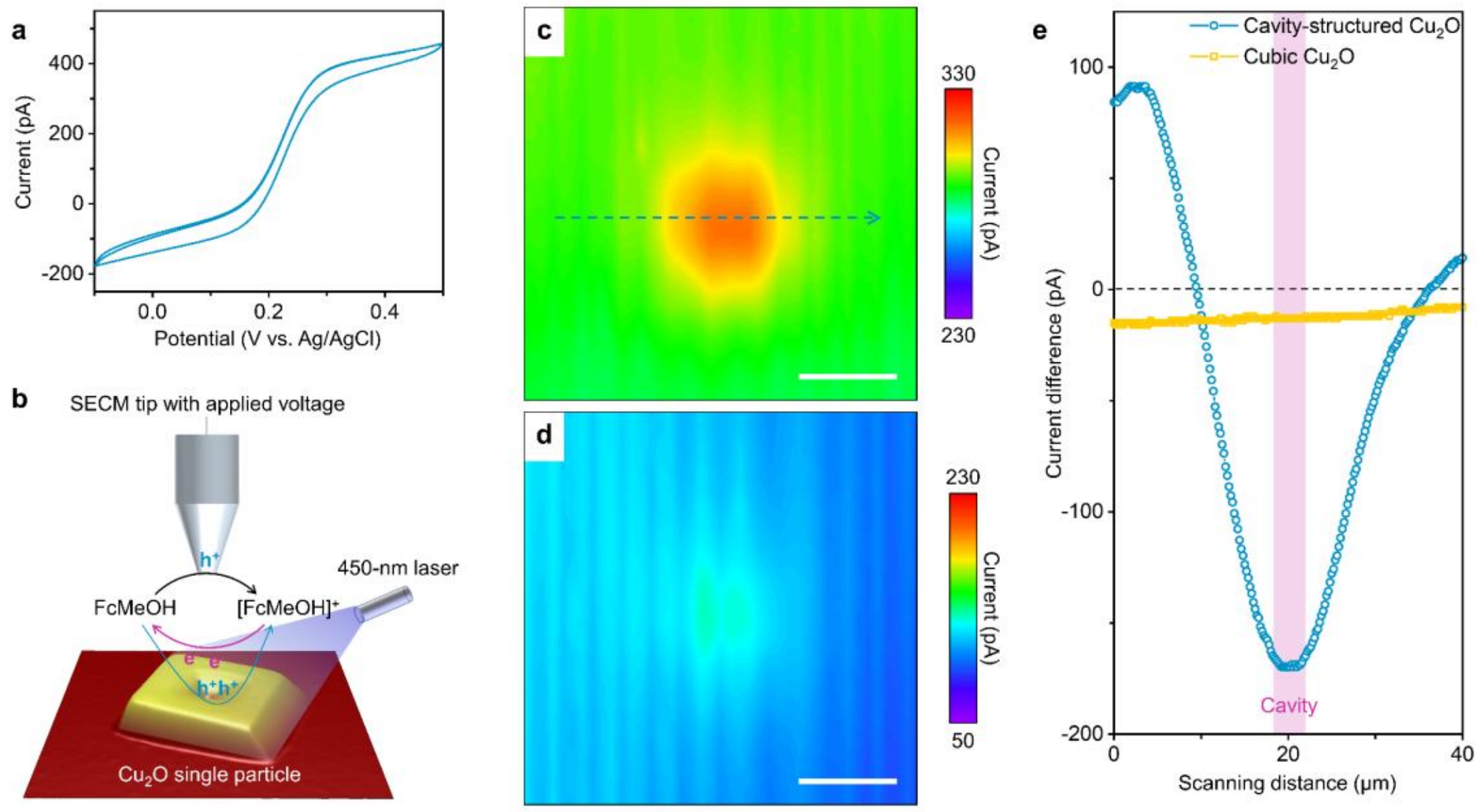

Supplementary Figure 32. SECM data. a, Cyclic voltammetry curves of the SECM tip measured in 0.1 M KCl solution containing 1 mM FcMeOH redox mediator. The voltammetry curves indicate a tip diameter of 1.3 μm (see Methods for details). **b**, Schematic illustration of SECM measurements on a cavity-structured Cu_2O particle. A 0.45-V bias voltage is applied to the SECM tip to oxidize FcMeOH to $[FcMeOH]^+$. When the tip approaches the particle, the oxidation current at the tip is enhanced in regions where photogenerated electrons are present, as these electrons reduce $[FcMeOH]^+$ to FcMeOH, providing positive feedback to the tip current. Conversely, the tip current decreases in regions where photogenerated holes accumulate, as the holes compete with the tip bias for driving FcMeOH oxidation. **c**,**d**, SECM images of a cavity-structured Cu_2O particle mapped in the dark (**c**) and under 450-nm light illumination (**d**). Scale bars, 5 μm. The images were obtained at a relatively fast scan rate to find the targeted particle. **e**, Fine scanning curves across the cavity-structured (dashed line in **c**) and cubic Cu_2O particles. The current difference was obtained by subtracting the dark current from the illuminated current, indicating local reaction

activity. Positive and negative current differences correspond to electron-mediated reduction and hole-mediated oxidation reactions, respectively. Compared with cubic Cu_2O, cavity-structured Cu_2O shows enhanced hole-mediated reactions in the cavity regions and electron-mediated reactions on the surrounding {001} surface regions. These results are consistent with the cavity-induced charge separation observed by SPVM and demonstrate that the cavity structure drives spatially separated redox reactions, improving photocatalytic performance. It should be noted that the measured spatial distributions of redox reactions in **e** are broader than the charge distributions obtained by SPVM due to diffusion of reactive and product species.

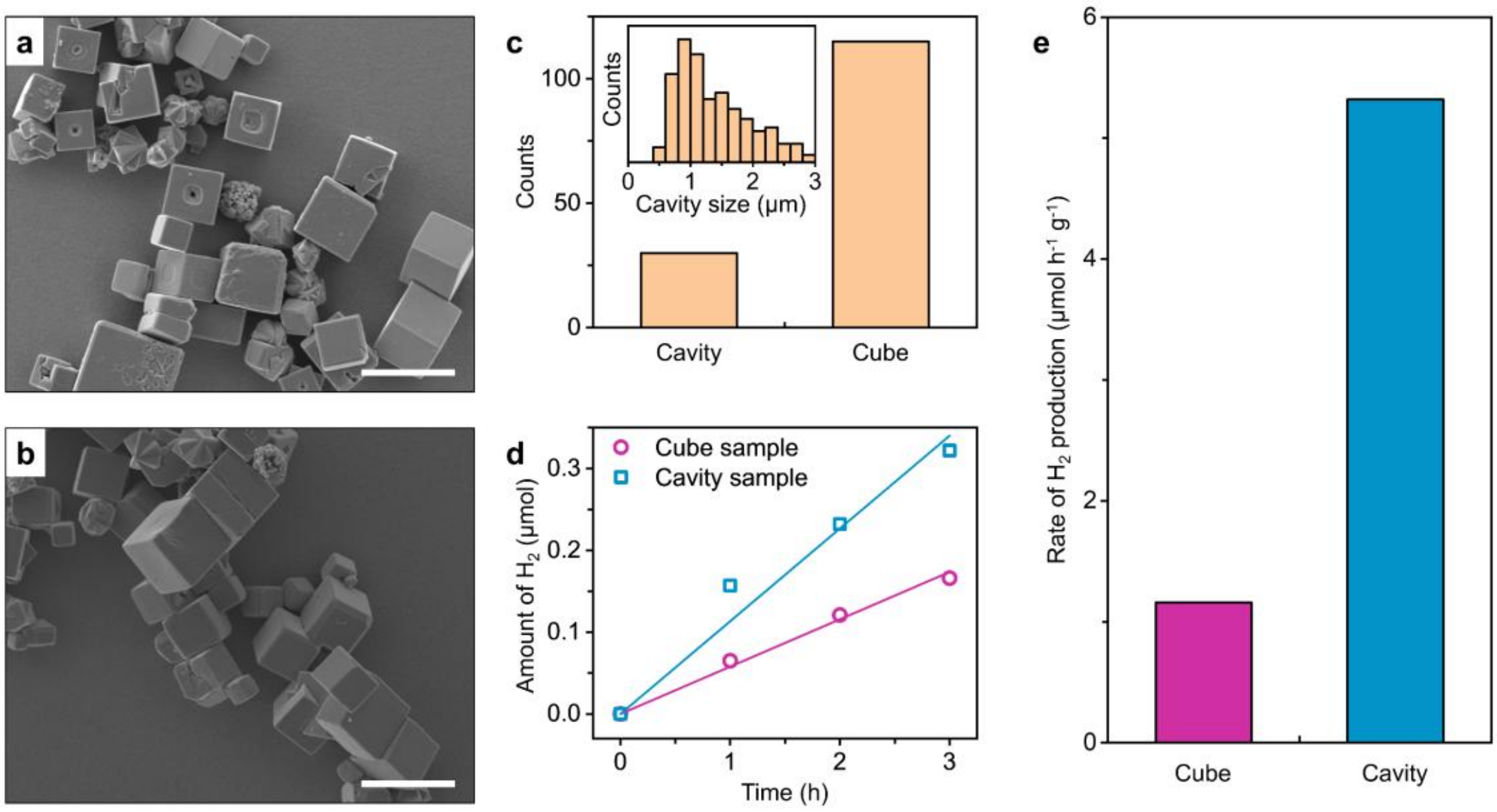

Supplementary Figure 33. Photocatalytic hydrogen production. a,**b**, SEM images of hydrothermally synthesized Cu_2O samples consisting of cavity-structured and cubic Cu_2O particles (**a**) and only cubic Cu_2O particles (**b**). Scale bars, 5 μm. The samples in **a** and **b** are denoted as cavity and cube samples, respectively. **c**, Statistics on the proportion of cavity-structured Cu_2O particles in the cavity sample, determined by counting particles in the SEM images. The proportion of cavity-structured Cu_2O particles is approximately 20%. Inset, Size distribution of the cavities, mainly ranging from 0.7 to 2.8 μm. **d**, Time course of photocatalytic H_2 evolution for cavity and cube samples. **e**, Comparison of H_2 production rates for cavity-structured and cubic Cu_2O particles. The activities were evaluated based on data in **c** and **d** after decoupling the contributions of cavity-structured and cubic Cu_2O particles. The results indicate that the photocatalytic activity of cavity-structured Cu_2O is approximately five times higher than that of cubic Cu_2O.

Note that the observed activity enhancement represents an average effect arising from cavities with a distribution of sizes. As charge separation efficiency increases with

cavity size within the 0.7–2.8 μm range, particles with smaller cavities likely contribute less effectively, while those with larger cavities contribute more significantly to the overall activity. Given that a substantial fraction of the cavities in the current sample remain below 1 μm, more precise control over the uniformity of cavity sizes, particularly by tuning them into the optimal micrometer-scale optical resonance range, could further enhance photocatalytic performance.

The enhancement of photocatalytic H_2 evolution induced by the cavity-structure strategy is compared with reported Cu_2O-based systems employing conventional strategies such as Schottky junction engineering [25], shape control [26], anisotropic facet engineering [9], and heterojunction construction [27]. These comparisons demonstrate that cavity engineering provides a fundamentally distinct yet comparably effective route for optimizing photocatalytic performance.

References

1. Siegfried MJ and Choi KS. Directing the architecture of cuprous oxide crystals during electrochemical growth. *Angew Chem Int Ed* 2005; **44**: 3218-3223.
2. Siegfried MJ and Choi KS. Elucidation of an overpotential-limited branching phenomenon observed during the electrocrystallization of cuprous oxide. *Angew. Chem Int Ed* 2008; **47**: 368-372.
3. Yu WW and Peng XG. Formation of high-quality CdS and other II-VI semiconductor nanocrystals in noncoordinating solvents: Tunable reactivity of monomers. *Angew Chem Int Ed* 2002; **41**: 2368-2371.
4. Kronik L and Shapira Y. Surface photovoltage phenomena: theory, experiment, and applications. *Surf Sci Rep* 1999; **37**: 1-206.
5. Chen R, Ni C and Zhu J *et al.* Surface photovoltage microscopy for mapping charge separation on photocatalyst particles. *Nat Protoc* 2024; **19**: 2250-2282.
6. Malerba C, Biccari F and Leonor Azanza Ricardo C *et al.* Absorption coefficient of bulk and thin film Cu_2O. *Sol Energy Mater Sol Cells* 2011; **95**: 2848-2854.
7. Liberal I, Mahmoud AM and Engheta N. Geometry-invariant resonant cavities. *Nat Commun* 2016; **7**: 10989.
8. Man MK, Margiolakis A and Deckoff-Jones S *et al.* Imaging the motion of electrons across semiconductor heterojunctions. *Nat Nanotechnol* 2017; **12**: 36-40.
9. Chen R, Ren Z and Liang Y *et al.* Spatiotemporal imaging of charge transfer in photocatalyst particles. *Nature* 2022; **610**: 296-301.
10. Dittrich T, Bonisch S and Zabel P *et al.* High precision differential measurement of

surface photovoltage transients on ultrathin CdS layers. *Rev Sci Instrum* 2008; **79**: 113903.

11. Dittrich T, Fengler S and Franke M. Transient surface photovoltage measurement over 12 orders of magnitude in time. *Rev Sci Instrum* 2017; **88**: 053904.
12. Bard AJ, Fan FRF and Pierce DT *et al.* Chemical Imaging of Surfaces with the Scanning Electrochemical Microscope. *Science* 1991; **254**: 68-74.
13. Ma W, Hu K and Chen Q *et al.* Electrochemical Size Measurement and Characterization of Electrodeposited Platinum Nanoparticles at Nanometer Resolution with Scanning Electrochemical Microscopy. *Nano Lett* 2017; **17**: 4354-4358.
14. Mora-Seró I, Dittrich T and Garcia-Belmonte G *et al.* Determination of spatial charge separation of diffusing electrons by transient photovoltage measurements. *J Appl Phys* 2006; **100**: 103705.
15. Chen R, Pang S and An H *et al.* Charge separation via asymmetric illumination in photocatalytic Cu_2O particles. *Nat Energy* 2018; **3**: 655-663.
16. Najafi E, Ivanov V and Zewail A *et al.* Super-diffusion of excited carriers in semiconductors. *Nat Commun* 2017; **8**: 15177.
17. Colchero J, Gil A and Baró AM. Resolution enhancement and improved data interpretation in electrostatic force microscopy. *Phys Rev B* 2001; **64**: 245403.
18. Lee KG, Kihm HW and Kihm JE *et al.* Vector field microscopic imaging of light. *Nat Photon* 2006; 1: 53-56.
19. Grioni M, van Acker JF and Czyzyk MT *et al.* Unoccupied electronic structure and

core-hole effects in the x-ray-absorption spectra of Cu_2O. *Phys Rev B* 1992; **45**: 3309-3318.

20. Önsten A, Weissenrieder J and Stoltz D *et al.* Role of Defects in Surface Chemistry on Cu_2O (111). *J Phys Chem C* 2013; **117**: 19357-19364.
21. Poulston S, Parlett PM and Stone P *et al.* Surface oxidation and reduction of CuO and Cu_2O studied using XPS and XAES. *Surf Interface Anal* 1996; **24**: 811-820.
22. Wei HH, Evans CM and Swartz BD *et al.* Colloidal semiconductor quantum dots with tunable surface composition. *Nano Lett* 2012; **12**: 4465-71.
23. Li Q, Ni C and Cui J *et al.* Impact of Reaction Environment on Photogenerated Charge Transfer Demonstrated by Sequential Imaging. *J Am Chem Soc* 2025; **147**: 9103-9110.
24. Chen R, Zhang D and Wang Z *et al.* Linking the Photoinduced Surface Potential Difference to Interfacial Charge Transfer in Photoelectrocatalytic Water Oxidation. *J Am Chem Soc* 2023; **145**: 4667-4674.
25. Wang L, Ge J and Wang A *et al.* Designing p-type semiconductor-metal hybrid structures for improved photocatalysis. *Angew Chem Int Ed* 2014; **53**: 5107-5111.
26. Kwon Y, Soon A and Han H *et al.* Shape effects of cuprous oxide particles on stability in water and photocatalytic water splitting. *J Mater Chem A* 2015; **3**: 156-162.
27. Yin W, Bai L and Zhu Y *et al.* Embedding Metal in the Interface of a p-n Heterojunction with a Stack Design for Superior Z-Scheme Photocatalytic Hydrogen Evolution. *ACS Appl Mater Interfaces* 2016; **8**: 23133-23142.
